# Supplementary material for: Practical iridium-catalyzed direct α-arylation of N-heteroarenes with (hetero)arylboronic acids by H2O-mediated H2 evolution
Source: Nat Commun. 2021 Jul 9;12:4206. doi: 10.1038/s41467-021-24468-z (PMC8270951; doi:10.1038/s41467-021-24468-z)
Supplement: Supplementary file 1 — Supplementary information [file 41467_2021_24468_MOESM1_ESM.pdf]

Supplementary Information

Practical iridium-catalyzed direct α-arylation of n-heteroarenes with (hetero)arylboronic acids by H_2O-mediated h_2 evolution

Liang Cao,[1] He Zhao,[1] Rongqing Guan,[1] Huanfeng Jiang,[1] Pierre. H. Dixneuf[2] and Min Zhang[1]✉

[1] Key Lab of Functional Molecular Engineering of Guangdong Province, School of Chemistry and Chemical Engineering, South China University of Technology, Guangzhou 510641, China.
[2] University of Rennes, ISCR, UMR CNRS 6226, 35000 Rennes, France.
✉Correspondence:
minzhang@scut.edu.cn (M.Z.).

Table of Contents

General information....[S2]

Typical procedure for the synthesis of product **C**....[S2]

Preparation of deuterated quinoline....[S4]

KIE experiment between quinoline and d_n-quinoline....[S4-S5]

Detection of $B(OH)_3$ by ^{11}B-NMR analysis....[S5]

Hydrogen detection....[S6-S7]

Synthesis of (Cp*)Ir(*L*-Pro)Cl....[S7]

Synthetic utility....[S7-S9]

Analytical data of the obtained compounds....[S10-S27]

NMR spectra of the obtained compounds....[S28-S94]

Supplementary references....[S95]

General information

All the obtained products were characterized by melting points (m.p.), ^{1}H-NMR, ^{13}C-NMR, and mass spectra (MS), the NMR spectra of the known compounds were found to be identical with the ones reported in the literatures. Additionally, all the new compounds were further characterized by high resolution mass spectra (HRMS). Melting points were measured on an Electrothemal SGW-X4 microscopy digital melting point apparatus and are uncorrected; ^{1}H-NMR, ^{13}C-NMR spectra were obtained on Bruker-500 or 400; Mass spectra were recorded on Trace DSQ GC/MS，High-resolution mass spectra (HRMS) were recorded on a JEOL JMS-600 spectrometer. Chemical shifts were reported in parts per million (ppm, δ) downfield from tetramethylsilane. Proton coupling patterns are described as singlet (s), doublet (d), triplet (t), multiplet (m); TLC was performed using commercially prepared 100-400 mesh silica gel plates (GF254), and visualization was affected at 254 nm; All the reagents were purchased from commercial sources (Energy Chemical, J&KChemic, TCI, Fluka, Acros, SCRC), and used without further purification.

Known compounds have been marked with CAS numbers, which can be compared with related references.[1-9]

Typical procedure I for the synthesis of α-arylation of N-heteroarenes

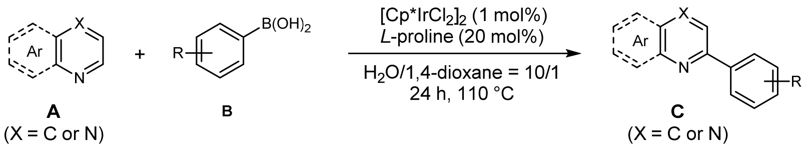

Under N_2 atmosphere, $[Cp^*IrCl_2]_2$ (1 mol%), *L*-proline (20 mol%), N-heteroarenes **A** (0.3 mmol), arylboronic acids **B** (0.36 mmol) and H_2O/1,4-dioxane (10/1, 1.5 mL) were introduced in a Schlenk tube (50 mL), successively. Then, the Schlenk tube was closed and the resulting mixture was stirred at 110 °C (oil bath temperature) for 24 h. After cooling down to room temperature, quenched with water, extracted with ethyl acetate (3×5 mL), and dried over anhydrous Na_2SO_4. The reaction mixture was concentrated by removing the solvent under vacuum, and the residue was purified by preparative TLC on silica, eluting with petroleum ether (60-90 °C) and ethyl acetate to give the desired product **C**.

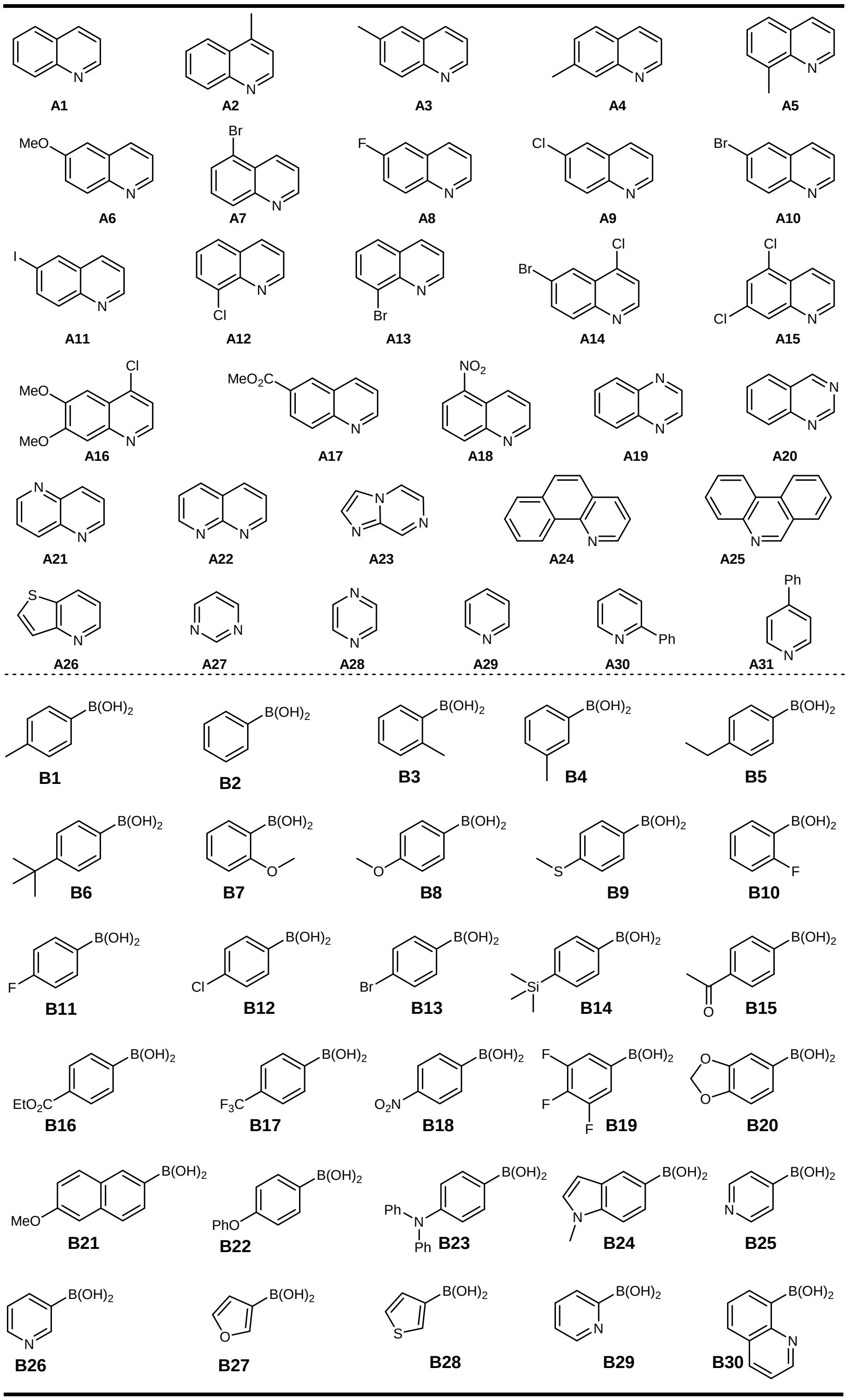

Supplementary Fig. 1. Substrates employed for the transformation. N-heteroarenes **A** and (hetero)arylboronic acids **B**.

Preparation of deuterated quinoline

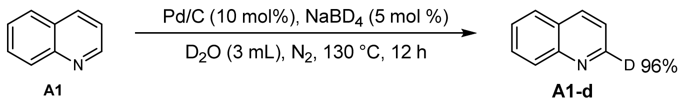

In a pressure tube, quinoline (1.0 mmol), palladium catalyst (10 %), 5 mol % $NaBD_4$ (98% D), and 3 mL D_2O (99% D) was introduced under the N_2 protection. After the reaction was stirred at 130 °C for 12 h, the mixture was cooled to room temperature and 3 mL acetonitrile were added, and the catalyst was removed by filtration. Finally, the residue was purified by preparative TLC on silica, eluting with ethyl acetate : petroleum ether = 10 : 1, to give the desired product **A1-d** with 96% deuterium ration at the α positions.

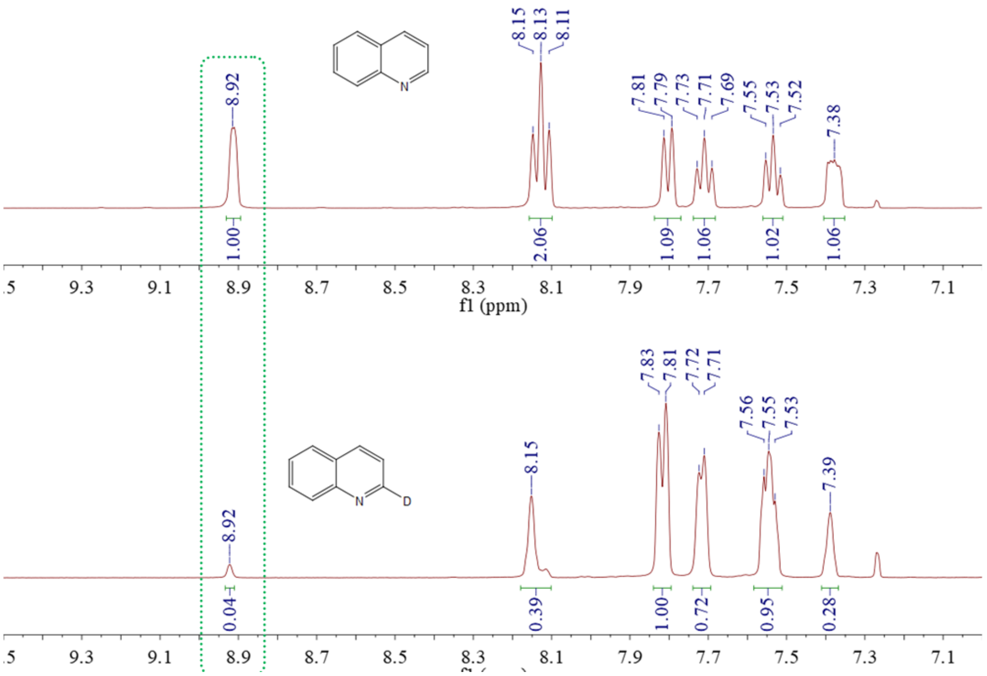

Supplementary Fig. 2. The ^{1}H-NMR (400 MHz, $CDCl_3$) of A1-d. The ^{1}H-NMR comparison of deuterated quinoline (**A1-d**) and quinoline (**A1**).

KIE experiment between quinoline and d_n-quinoline

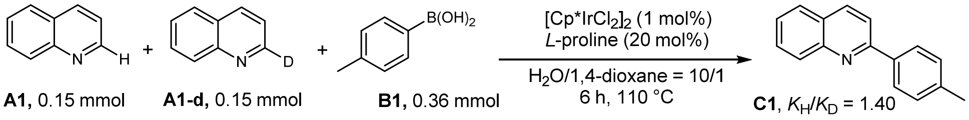

Under N_2 atmosphere, quinoline **A1** (0.15 mmol), **A1-d** (0.15 mmol), 4-tolylboronic acid **B1** (0.36 mmol), $[Cp^*IrCl_2]_2$ (1 mol%), *L*-proline (20 mol%) and H_2O/1,4-dioxane (10/1, 1.5 mL) were introduced in a Schlenk tube (50 mL), successively. Then, the Schlenk tube was closed and the resulting mixture was stirred at 110 °C (oil bath temperature) for 6 h, quenched with water, extracted with ethyl acetate (3×5 mL), and dried over anhydrous Na_2SO_4. Residual starting material (mixture of d_n-quinoline) was recovered by preparative TLC on silica, which was characterized by ^{1}H NMR spectra. Peak areas at 8.93 ppm were compared to give the ratio of d_n-quinoline **A1-d** to quinoline **A1** in residual material. The experiment

repeated three times and calculated KIE values are 1.50, 1.38 and 1.33. The average K_H/K_D = 1.40. Representative ^{1}H NMR spectra copy was shown below:

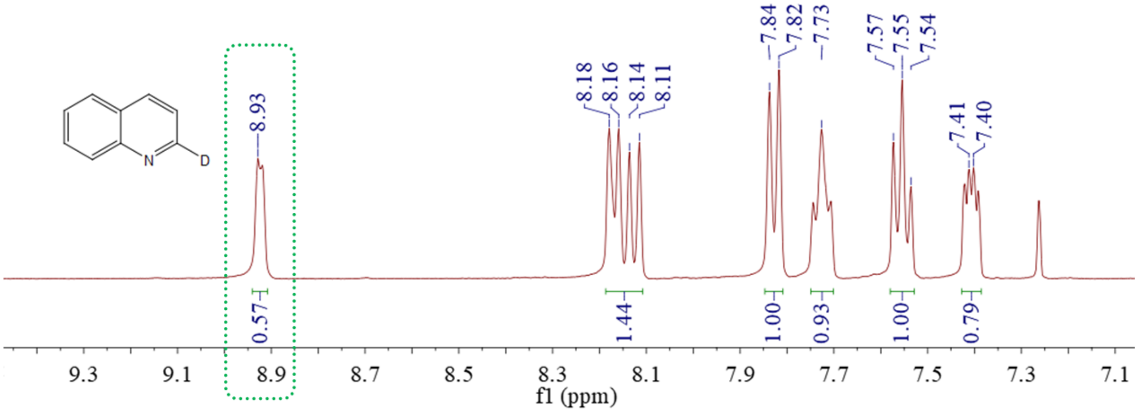

Supplementary Fig. 3. The ^{1}H-NMR (400 MHz, $CDCl_3$) of quinoline. KIE experiment between quinoline (**A1**) and d_n-quinoline (**A1-d**).

Detection of $B(OH)_3$ by ^{11}B-NMR analysis

Under N_2 atmosphere, [Cp*$IrCl_2$]$_2$ (1 mol%), *L*-proline (20 mol%), quinoline **A1** (0.3 mmol), 4-tolylboronic acid **B1** (0.36 mmol) and D_2O (1.5 mL) were introduced in a Schlenk tube (50 mL), successively. Then, the Schlenk tube was closed and the resulting mixture was stirred at 110 °C (oil bath temperature) for 24 h. After cooling down to room temperature, immediately test the boron spectrum (^{11}B-NMR) and compare with the standard boric acid ($B(OH)_3$) sample.

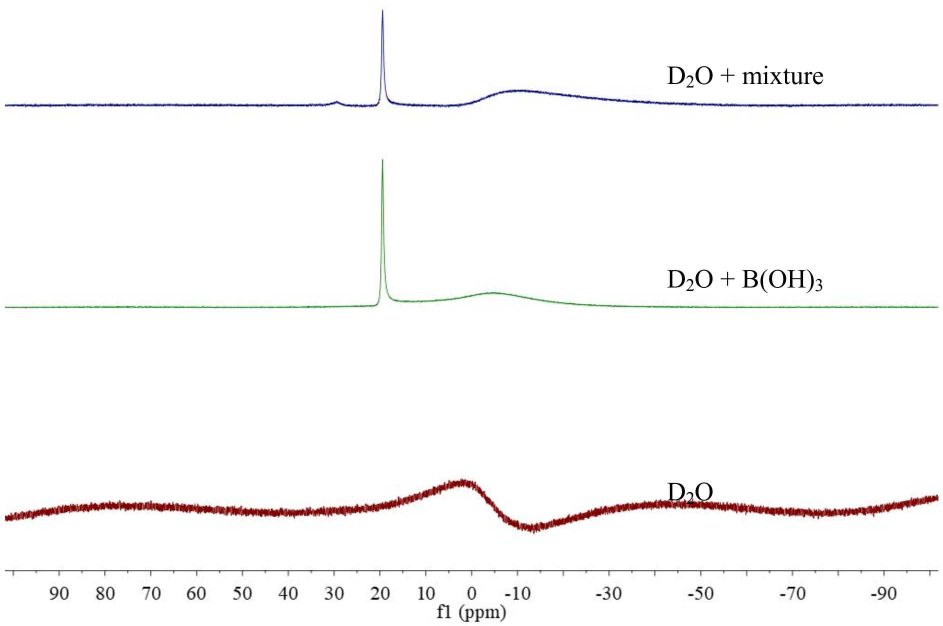

Supplementary Fig. 4. The ^{11}B NMR (160 MHz, D_2O) spectrum of reaction. Detection of $B(OH)_3$ by ^{11}B-NMR analysis.

Hydrogen detection

Under N_2 atmosphere, $[Cp^*IrCl_2]_2$ (1 mol%), *L*-proline (20 mol%), quinoline **A1** (0.3 mmol), 4-tolylboronic acid **B1** (0.36 mmol) and H_2O/1,4-dioxane (10/1, 1.5 mL) were introduced in a Schlenk tube (50 mL), successively. Then, the Schlenk tube was closed and the resulting mixture was stirred at 110 °C (oil bath temperature) for 12 h.The gas sample was collected from the above of the reaction solution by using methane as the internal standard, which was detected by gas chromatography (Agilent 7890A). According to the result shown in supplementary Fig. 5, it approves the generation of H_2 (1.37 mL) during the reaction.

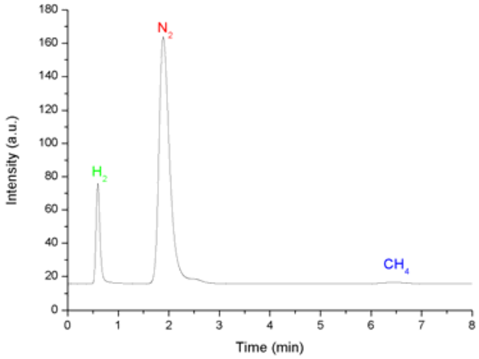

Supplementary Fig. 5. GC analysis of the sample taken from the reaction. CH_4 was used as the internal standard for calibration purpose.

Supplementary Table 1. Data from GC analysis of the sample from the reaction

Gas	H_2	CH_4
S%	354.0929	36.3599
V (mL)	0.50	**1.37**

$$V(H_2) = 36.3599/54.0929*0.5\ mL*K=1.37\ mL$$

Note：*K* is the calibration factor, which is calculated by mixing 1 mL of hydrogen and 1 mL of methane, and detected by gas chromatography (**Supplementary Fig. 6** and **Supplementary Table 2**).

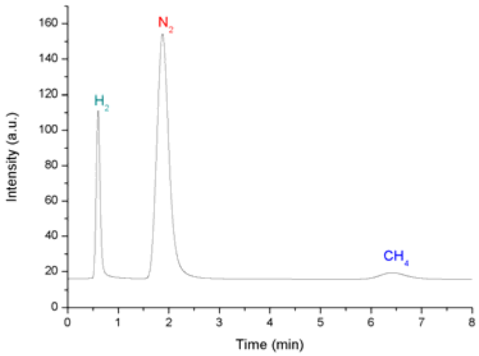

Supplementary Fig. 6. GC analysis of the standard sample. The results are used for the calculation of calibration factor *K*.

Supplementary Table 2. Data from GC analysis of the standard sample

Gas	H_2	CH_4
S%	564.7567	137.5764
V (mL)	1.00	1.00

K=564.7567/137.5764

Synthesis of (Cp*)Ir(*L*-Pro)Cl

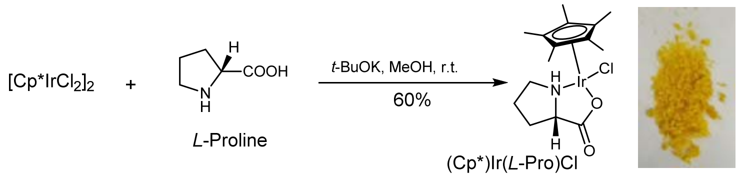

Under N_2 atmosphere, $[Cp^*IrCl_2]_2$ (50 μmol) and *L*-proline (100 μmol) were introduced in MeOH (5 mL), and *t*-BuOK (105 μmol) in MeOH (2 mL) was then slowly added. The orange suspension turned into a homogeneous yellow solution at room temperature upon stirring. The insoluble solid was then filtered with funnel, and the solvent was removed under vacuum to afford the yellow residue, which was further disolved in acetone, and the resulting suspension was filtered to remove the insoluable KCl. The filtrate was again concentrated to dryness and the residue was dissolved in CH_2Cl_2, then filtered over a pad of Celite, and the filter cake was washed with CH_2Cl_2 (2 mL). The combined filtrates were layered in pentane to precipitate (15 mL) for 2 days, the yellow crystal product (18 mg, 60%) was obtaied after removal of the solvent. ^{1}H NMR (500 MHz, $CDCl_3$) δ 4.85 (s, 1H), 4.06-4.01 (m, 1H), 3.64-3.58 (m, 1H), 2.98-2.90 (m, 1H), 2.29-2.22 (m, 1H), 2.06-1.99 (m, 1H), 1.94-1.92 (m, 1H), 1.71-1.70 (m, 1H), 1.68 (s, 15H); ^{13}C NMR (126 MHz, $CDCl_3$) δ 184.5, 84.2, 62.5, 54.8, 28.8, 27.1, 9.2. HRMS (ESI): Calcd. for $C_{15}H_{24}ClIrNO_2$: 478.1119; found: 478.1111.

Synthetic utility

(1) gram-scale synthesis of 2-(*p*-tolyl)quinoline (**C1**).

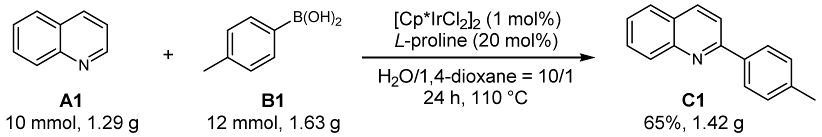

Under N_2 atmosphere, $[Cp^*IrCl_2]_2$ (1 mol%), *L*-proline (20 mol%), quinoline **A1** (10 mmol), 4-tolylboronic acid **B1** (12 mmol) and H_2O/1,4-dioxane (10/1, 10 mL) were introduced in a Schlenk tube (100 mL), successively. Then, the Schlenk tube was closed and the resulting mixture was stirred at 110 °C (oil bath temperature) for 24 h. After cooling down to room temperature, quenched with water, extracted with ethyl acetate (3×15 mL), and dried over anhydrous Na_2SO_4. The reaction mixture was concentrated by removing the solvent under vacuum, and the residue was purified by flash column chromatography, eluting with petroleum ether (60-90 °C): ethyl acetate (30 : 1) to give product **C1**.

(2) Product diversification transformation (**C1’**). **CAS**: 123612-59-9.

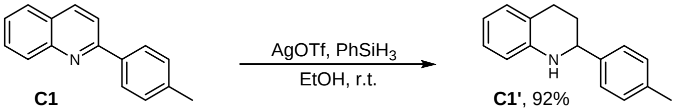

A Schlenk tube was charged with **C1** (0.2 mmol), AgOTf (5 mol%) and EtOH (1.5 mL). Then $PhSiH_3$ (4.0 equiv) was dropwise added by syringe over three minute under air and reacted at room temperature for 15 min. The reaction mixture was concentrated by removing the solvent under vacuum, and the residue was purified by flash column chromatography, eluting with petroleum ether (60-90 $^{\circ}$C): ethyl acetate (20 : 1) to give product **C1’**. Colorless oil liquid (41 mg, 92%); TCL (petroleum ether : ethyl acetate = 20 : 1): R_f = 0.68; ^{1}H NMR (400 MHz, $CDCl_3$) δ 7.39 (d, J = 8.0 Hz, 2H), 7.27 (d, J = 7.8 Hz, 2H), 7.14-7.09 (m, 2H), 6.76 (t, J = 7.3 Hz, 1H), 6.62 (d, J = 7.7 Hz, 1H), 4.51-4.47 (m, 1H), 4.08 (s, 1H), 3.07-2.98 (m, 1H), 2.88-2.81 (m, 1H), 2.47 (s, 3H), 2.24-2.17 (m, 1H), 2.13-2.04 (m, 1H); ^{13}C NMR (101 MHz, $CDCl_3$) δ 144.9, 142.0, 137.1, 129.4, 129.3, 127.0, 126.6, 121.0, 117.2, 114.1, 56.1, 31.1, 26.6, 21.2; MS (EI, m/z): 223.27 $[M]^+$.

(3) Product diversification transformation (**C37’**).

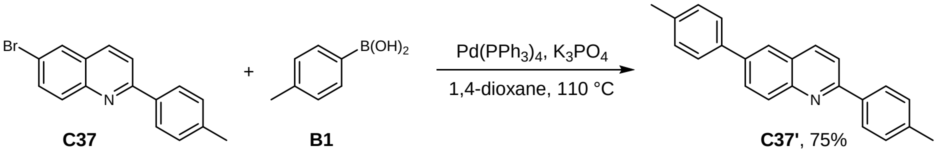

Under N_2 atmosphere, $Pd(PPh_3)_4$ (10 mol%), K_3PO_4 (1.5 equiv), **C37** (0.20 mmol), 4-tolylboronic acid **B1** (1.5 equiv) and 1,4-dioxane (1.5 mL) were introduced in a Schlenk tube (50 mL), successively. Then, the Schlenk tube was closed and the resulting mixture was stirred at 110 $^{\circ}$C (oil bath temperature) for 15 h. After cooling down to room temperature, the reaction mixture was concentrated by removing the solvent under vacuum, and the residue was purified by preparative TLC on silica, eluting with petroleum ether (60-90 $^{\circ}$C): ethyl acetate (15 : 1) to give product **C37’**. Brown solid (46 mg, 75%), m.p.: 119.2-120.9 °C; TCL (petroleum ether : ethyl acetate = 15 : 1): R_f = 0.52; ^{1}H NMR (500 MHz, $CDCl_3$) δ 8.23 (d, J = 8.6 Hz, 1H), 8.21 (d, J = 9.4 Hz, 1H), 8.09 (d, J = 8.0 Hz, 2H), 7.98 (s, 1H), 7.97 (d, J = 8.0 Hz, 1H), 7.88 (d, J = 8.6 Hz, 1H), 7.64 (d, J = 8.0 Hz, 2H), 7.34 (d, J = 7.9 Hz, 2H), 7.31 (d, J = 7.9 Hz, 2H), 2.44 (s, 3H), 2.43 (s, 3H); ^{13}C NMR (126 MHz, $CDCl_3$) δ 157.1, 139.4, 138.8, 137.6, 137.5, 136.9, 136.8, 134.9, 130.0, 129.7, 129.6, 129.3, 127.4, 127.3, 127.2, 124.8, 119.2, 21.4, 21.2. HRMS (ESI): Calcd. for $C_{23}H_{20}N$: 310.1590; found: 310.1584.

(4) Access to potentially biomedical molecules (***p*-tolyl-hydroquinidine hybrid**).

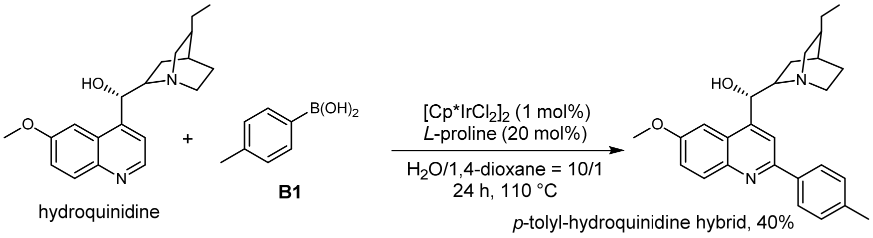

Under N_2 atmosphere, $[Cp^*IrCl_2]_2$ (1 mol%), *L*-proline (20 mol%), hydroquinidine (0.2 mmol), 4-tolylboronic acid **B1** (0.30 mmol) and H_2O/1,4-dioxane (10/1, 1.5 mL) were introduced in a Schlenk tube

(50 mL), successively. Then, the Schlenk tube was closed and the resulting mixture was stirred at 110 °C (oil bath temperature) for 24 h. After cooling down to room temperature, quenched with water, extracted with ethyl acetate (3×5 mL), and dried over anhydrous Na_2SO_4. The reaction mixture was concentrated by removing the solvent under vacuum, and the residue was purified by preparative TLC on silica, eluting with petroleum ether (60-90 °C), ethyl acetate and methanol (PE : EA : CH_3OH = 5 : 1 : 1) to give the desired product *p*-tolyl-hydroquinidine hybrid. Brown solid (33 mg, 40%), m.p.: 149.0-150.5 °C; TCL (petroleum ether : ethyl acetate methanol = 5 : 1 : 1): R_f = 0.35; ^{1}H NMR (500 MHz, $CDCl_3$) δ 7.92-7.90 (m, 2H), 7.88 (d, J = 8.0 Hz, 2H), 7.21 (d, J = 7.9 Hz, 2H), 7.17 (d, J = 9.2 Hz, 1H), 6.96 (d, J = 2.2 Hz, 1H), 6.01 (s, 1H), 3.78 (s, 3H), 3.75-3.72 (m, 1H), 3.19-3.08 (m, 4H), 2.45-2.41 (m, 1H), 2.38 (s, 3H), 2.25-2.20 (m, 1H), 1.62-1.54 (m, 5H), 1.27-1.25 (m, 2H), 0.90 (t, J = 7.1 Hz, 3H); ^{13}C NMR (126 MHz, $CDCl_3$) δ 157.9, 154.3, 145.8, 143.9, 139.0, 136.7, 131.5, 129.4, 127.2, 124.5, 122.0, 116.1, 100.1, 68.2, 60.0, 55.8, 50.2, 49.5, 35.9, 25.5, 24.9, 24.5, 21.3, 18.4, 11.7. HRMS (ESI): Calcd. for $C_{17}H_{16}N$: 417.2537; found: 417.2531.

Analytical data of the obtained compounds

(1) 2-(*p*-tolyl)quinoline (C1). CAS: 24667-94-5.

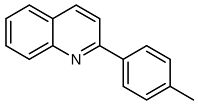

Using general procedure I. White solid (47 mg, 72%), m.p.: 85.7-86.9 °C; TCL (petroleum ether : ethyl acetate = 20 : 1): R_f = 0.68; ^{1}H NMR (400 MHz, $CDCl_3$) δ 8.05 (t, *J* = 9.5 Hz, 2H), 7.97 (d, *J* = 7.7 Hz, 2H), 7.72 (d, *J* = 8.6 Hz, 1H), 7.67 (d, *J* = 8.1 Hz, 1H), 7.59 (t, *J* = 7.6 Hz, 1H), 7.38 (t, *J* = 7.5 Hz, 1H), 7.21 (d, *J* = 7.7 Hz, 2H), 2.32 (s, 3H); ^{13}C NMR (101 MHz, $CDCl_3$) δ 157.3, 148.3, 139.4, 136.9, 136.7, 129.7, 129.6, 127.5, 127.1, 126.1, 118.9, 21.4. MS (EI, m/z): 219.24 $[M]^+$.

(2) 2-phenylquinoline (C2). CAS: 612-96-4.

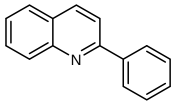

Using general procedure I. White solid (41 mg, 68%), m.p.: 72.7-73.6 °C; TCL (petroleum ether : ethyl acetate = 20 : 1): R_f = 0.68; ^{1}H NMR (400 MHz, $CDCl_3$) δ 8.23-8.18 (m, 4H), 7.87 (d, *J* = 8.6 Hz, 1H), 7.82 (d, *J* = 8.1 Hz, 1H), 7.74 (t, *J* = 7.7 Hz, 1H), 7.57-7.52 (m, 3H), 7.51-7.46 (m, 1H); ^{13}C NMR (101 MHz, $CDCl_3$) δ 157.4, 148.4, 139.7, 136.8, 129.8, 129.7, 129.4, 128.9, 127.6, 127.5, 127.2, 126.3, 119.0. MS (EI, m/z): 205.29 $[M]^+$.

(3) 2-(*o*-tolyl)quinoline (C3). CAS: 52146-06-2.

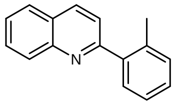

Using general procedure I. Colorless oil liquid (36 mg, 55%); TCL (petroleum ether : ethyl acetate = 20 : 1): R_f = 0.65; ^{1}H NMR (400 MHz, $CDCl_3$) δ 8.16 (d, *J* = 8.3 Hz, 2H), 7.82 (d, *J* = 8.1 Hz, 1H), 7.71 (t, *J* = 8.1 Hz, 1H), 7.54-7.48 (m, 3H), 7.33-7.28 (m, 3H), 2.41 (s, 3H); ^{13}C NMR (101 MHz, $CDCl_3$) δ 160.3, 147.9, 140.8, 136.1, 136.0, 130.9, 129.8, 129.7, 128.6, 127.5, 126.8, 126.4, 126.1, 122.4, 20.4. MS (EI, m/z): 219.27 $[M]^+$.

(4) 2-(*m*-tolyl)quinoline (C4). CAS: 24641-30-3.

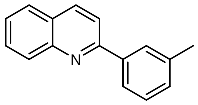

Using general procedure I. Colorless oil liquid (46 mg, 70%); TCL (petroleum ether : ethyl acetate = 20 : 1): R_f = 0.68; ^{1}H NMR (400 MHz, $CDCl_3$) δ 8.17 (d, *J* = 8.5 Hz, 1H), 8.14 (d, *J* = 8.7 Hz, 1H), 8.00 (s, 1H), 7.90 (d, *J* = 7.7 Hz, 1H), 7.81 (d, *J* = 8.6 Hz, 1H), 7.77 (d, *J* = 8.1 Hz, 1H), 7.69 (t, *J* = 7.7 Hz, 1H), 7.48 (t, *J* = 7.0 Hz, 1H), 7.39 (t, *J* = 7.6 Hz, 1H), 7.25 (d, *J* = 7.5 Hz, 1H), 2.45 (s, 3H); ^{13}C NMR (101 MHz, $CDCl_3$) δ 157.6, 148.3, 139.7, 138.5, 136.7, 130.2, 129.8, 129.6, 128.8, 128.3, 127.5, 127.2, 126.2, 124.8, 119.2, 21.6, 21.6. MS (EI, m/z): 219.30 $[M]^+$.

(5) 2-(4-ethylphenyl)quinoline (C5). CAS: 860200-72-2.

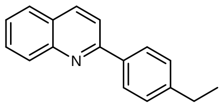

Using general procedure I. White solid (53 mg, 75%), m.p.: 49.8-51.2 °C; TCL (petroleum ether : ethyl acetate = 20 : 1): R_f = 0.65; ^{1}H NMR (400 MHz, $CDCl_3$) δ 8.16 (d, *J* = 8.5 Hz, 1H), 8.10 (d, *J* = 8.6 Hz, 1H), 8.07 (d, *J* = 8.2 Hz, 2H), 7.79 (d, *J* = 8.6 Hz, 1H), 7.74 (d, *J* = 8.1 Hz, 1H), 7.67 (t, *J* = 7.0 Hz, 1H), 7.45 (t, *J* = 7.5 Hz, 1H), 7.32 (d, *J* = 8.3 Hz, 2H), 2.70 (q, *J* = 7.6 Hz, 2H), 1.26 (t, *J* = 7.6 Hz, 3H); ^{13}C NMR (101 MHz, $CDCl_3$) δ 157.4, 148.4, 145.8, 137.2, 136.7, 129.7, 129.6, 128.4, 127.6, 127.5, 127.2, 126.1, 118.9, 28.8, 15.6. MS (EI, m/z): 233.32 $[M]^+$.

(6) 2-(4-(*tert*-butyl)phenyl)quinoline (C6). CAS: 917114-13-7.

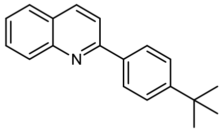

Using general procedure I. White solid (64 mg, 81%), m.p.: 71.2-72.8 °C; TCL (petroleum ether : ethyl acetate = 20 : 1): R_f = 0.65; ^{1}H NMR (400 MHz, $CDCl_3$) δ 8.19 (t, *J* = 7.6 Hz, 2H), 8.12 (d, *J* = 8.1 Hz, 2H), 7.86 (d, *J* = 8.6 Hz, 1H), 7.81 (d, *J* = 8.1 Hz, 1H), 7.73 (t, *J* = 7.6 Hz, 1H), 7.57 (d, *J* = 8.1 Hz, 2H), 7.52 (t, *J* = 7.5 Hz, 1H), 1.41 (s, 9H); ^{13}C NMR (101 MHz, $CDCl_3$) δ 156.3, 151.5, 147.3, 135.9, 135.6, 128.6, 128.5, 126.4, 126.3, 126.1, 125.0, 124.8, 117.9, 33.7, 30.3. MS (EI, m/z): 261.31 $[M]^+$.

(7) 2-(2-methoxyphenyl)quinoline (C7). CAS: 72195-25-6.

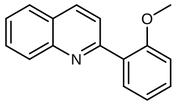

Using general procedure I. Colorless oil liquid (37 mg, 52%); TCL (petroleum ether : ethyl acetate = 20 : 1): R_f = 0.40; ^{1}H NMR (400 MHz, $CDCl_3$) δ 8.17 (d, *J* = 8.5 Hz, 1H), 8.09 (d, *J* = 8.6 Hz, 1H), 7.88-7.84 (m, 2H), 7.78 (d, *J* = 7.2 Hz, 1H), 7.67 (t, *J* = 7.0 Hz, 1H), 7.48 (t, *J* = 8.1 Hz, 1H), 7.41-7.37 (m, 1H), 7.11 (t, *J* = 7.5 Hz, 1H), 7.00 (d, *J* = 8.3 Hz, 1H), 3.81 (s, 3H); ^{13}C NMR (101 MHz, $CDCl_3$) δ 157.3, 157.2, 148.4, 135.1, 131.5, 130.4, 129.8, 129.7, 129.2, 127.4, 127.1, 126.2, 123.5, 121.3, 111.5, 55.7. MS (EI, m/z): 235.26 $[M]^+$.

(8) 2-(4-methoxyphenyl)quinoline (C8). CAS: 16032-40-9.

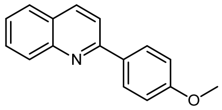

Using general procedure I. White solid (55 mg, 78%), m.p.: 121.1-122.4 °C; TCL (petroleum ether : ethyl acetate = 20 : 1): R_f = 0.42; ^{1}H NMR (400 MHz, $CDCl_3$) δ 8.14-8.09 (m, 3H), 8.07 (d, *J* = 8.7 Hz, 1H), 7.74 (t, *J* = 8.2 Hz, 2H), 7.67 (t, *J* = 7.0 Hz, 1H), 7.44 (t, *J* = 6.9 Hz, 1H), 7.00 (d, *J* = 8.9 Hz, 2H), 3.82 (s, 3H); ^{13}C NMR (101 MHz, $CDCl_3$) δ 160.9, 156.9, 148.4, 136.6, 132.3, 129.6, 128.9, 127.5, 127.0, 125.9, 118.5, 114.3, 55.4, 55.4. MS (EI, m/z): 235.29 $[M]^+$.

(9) 2-(4-(methylthio)phenyl)quinoline (C9). CAS: 1380751-78-9.

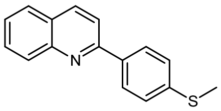

Using general procedure I. Brown solid (53 mg, 70%), m.p.: 125.6-126.9 °C; TCL (petroleum ether : ethyl acetate = 20 : 1): R_f = 0.41; ^{1}H NMR (400 MHz, $CDCl_3$) δ 8.20 (d, *J* = 8.8 Hz, 1H), 8.17 (d, *J* = 8.7 Hz, 1H), 8.11 (d, *J* = 8.1 Hz, 2H), 7.85 (d, *J* = 8.6 Hz, 1H), 7.81 (d, *J* = 8.2 Hz, 1H), 7.72 (t, *J* = 7.6 Hz, 1H), 7.51 (t, *J* = 7.4 Hz, 1H), 7.39 (d, *J* = 8.0 Hz, 2H), 2.55 (s, 3H); ^{13}C NMR (101 MHz, $CDCl_3$) δ 156.6, 148.2, 140.5, 136.9, 136.1, 129.8, 129.5, 127.9, 127.5, 127.2, 126.5, 126.3, 118.6, 15.6. MS (EI, m/z): 251.25 $[M]^+$.

(10) 2-(2-fluorophenyl)quinoline (C10). CAS: 2836-41-1.

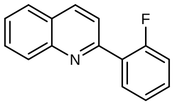

Using general procedure I. Colorless oil liquid (35 mg, 53%); TCL (petroleum ether : ethyl acetate = 20 : 1): R_f = 0.60; ^{1}H NMR (500 MHz, $CDCl_3$) δ 8.17 (t, *J* = 7.8 Hz, 2H), 8.10 (t, *J* = 7.8 Hz, 1H), 7.87-7.84 (m, 1H), 7.80 (d, *J* = 8.1 Hz, 1H), 7.71 (t, *J* = 8.2 Hz, 1H), 7.52 (t, *J* = 7.5 Hz, 1H), 7.42-7.37 (m, 1H), 7.29 (t, *J* = 7.5 Hz, 1H), 7.20-7.15 (m, 1H). ^{13}C NMR (126 MHz, $CDCl_3$) δ 160.8 (d, *J* = 198 Hz), 154.1, 148.4, 136.2, 131.6, 130.9, 130.8, 129.8, 129.6, 127.5, 127.3, 126.7, 124.7, 122.5 (d, *J* = 7 Hz), 116.3 (d, *J* = 18 Hz); ^{19}F NMR (471 MHz, $CDCl_3$) δ -117.11. MS (EI, m/z): 223.26 $[M]^+$.

(11) 2-(4-fluorophenyl)quinoline (C11). CAS: 323-91-1.

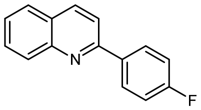

Using general procedure I. White solid (40 mg, 60%), m.p.: 91.2-92.7 °C; TCL (petroleum ether : ethyl acetate = 20 : 1): R_f = 0.64; ^{1}H NMR (400 MHz, $CDCl_3$) δ 8.16-8.11 (m, 4H), 7.79-7.74 (m, 2H), 7.70 (t, *J* = 7.6 Hz, 1H), 7.49 (t, *J* = 7.5 Hz, 1H), 7.18 (t, *J* = 8.7 Hz, 2H); ^{13}C NMR (101 MHz, $CDCl_3$) δ 163.8 (d, *J* = 248 Hz), 156.2, 148.2, 136.9, 129.8, 129.7, 129.4 (d, *J* = 8 Hz), 127.5, 127.1, 126.4, 118.6, 115.8 (d, *J* = 21 Hz); ^{19}F NMR (376 MHz, $CDCl_3$) δ -112.43. MS (EI, m/z): 223.25 $[M]^+$.

(12) 2-(4-chlorophenyl)quinoline (C12). CAS: 24698-70-2.

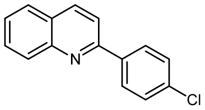

Using general procedure I. Yellow solid (43 mg, 60%), m.p.: 109.5 -110.9 °C; TCL (petroleum ether : ethyl acetate = 20 : 1): R_f = 0.64; ^{1}H NMR (400 MHz, $CDCl_3$) δ 8.24 (d, *J* = 8.6 Hz, 1H), 8.19 (d, *J* = 8.5 Hz, 1H), 8.15 (d, *J* = 7.5 Hz, 2H), 7.85 (d, *J* = 8.3 Hz, 2H), 7.76 (t, *J* = 7.6 Hz, 1H), 7.57 (d, *J* = 7.6 Hz, 1H), 7.52 (d, *J* = 8.1 Hz, 2H); ^{13}C NMR (101 MHz, $CDCl_3$) δ 156.0, 148.2, 138.0, 137.0, 135.6, 129.9, 129.7, 129.0, 128.9, 127.5, 127.2, 126.5, 118.6. MS (EI, m/z): 239.23 $[M]^+$.

(13) 2-(4-bromophenyl)quinoline (C13). CAS: 24641-31-4.

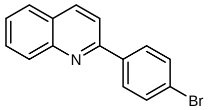

Using general procedure I. Yellow solid (52 mg, 61%), m.p.: 116.2 -117.5 °C; TCL (petroleum ether : ethyl acetate = 20 : 1): R_f = 0.64; ^{1}H NMR (400 MHz, $CDCl_3$) δ 8.22 (d, *J* = 8.7 Hz, 1H), 8.16 (d, *J* = 8.2 Hz, 1H), 8.05 (d, *J* = 7.7 Hz, 2H), 7.83 (d, *J* = 8.1 Hz, 2H), 7.73 (t, *J* = 7.4 Hz, 1H), 7.65 (d, *J* = 7.8 Hz, 2H), 7.54 (t, *J* = 7.3 Hz, 1H); ^{13}C NMR (101 MHz, $CDCl_3$) δ 155.0, 147.2, 137.4, 135.9, 130.9, 128.8, 128.7, 128.0, 126.4, 126.2, 125.5, 122.9, 117.4. MS (EI, m/z): 283.15 $[M]^+$.

(14) 2-(4-(trimethylsilyl)phenyl)quinoline (C14). CAS: 18557-94-3.

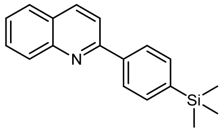

Using general procedure I. Yellow solid (52 mg, 62%), m.p.: 74.5-75.8 °C; TCL (petroleum ether : ethyl acetate = 20 : 1): R_f = 0.63; ^{1}H NMR (400 MHz, $CDCl_3$) δ 8.10 (d, *J* = 8.3 Hz, 2H), 8.04 (d, *J* = 7.4 Hz, 2H), 7.77 (d, *J* = 8.5 Hz, 1H), 7.72 (d, *J* = 8.1 Hz, 1H), 7.64 (d, *J* = 7.6 Hz, 1H), 7.60 (d, *J* = 7.6 Hz, 2H), 7.42 (t, *J* = 7.3 Hz, 1H), 0.23 (s, 9H); ^{13}C NMR (101 MHz, $CDCl_3$) δ 158.6, 149.5, 143.0, 141.1, 137.9, 135.0, 130.9, 130.8, 128.6, 128.4, 127.9, 127.4, 120.2, 2.2. MS (EI, m/z): 277.28 $[M]^+$.

(15) 1-(4-(quinolin-2-yl)phenyl)ethan-1-one (C15). CAS: 221910-24-3.

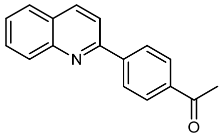

Using general procedure I. White solid (38 mg, 51%), m.p.: 133.5-134.2 °C; TCL (petroleum ether : ethyl acetate = 10 : 1): R_f = 0.25; ^{1}H NMR (400 MHz, $CDCl_3$) δ 8.27 (d, *J* = 8.4 Hz, 2H), 8.24 (d, *J* = 8.7 Hz, 1H), 8.20 (d, *J* = 8.5 Hz, 1H), 8.10 (d, *J* = 8.4 Hz, 2H), 7.89 (d, *J* = 8.6 Hz, 1H), 7.84 (d, *J* = 8.1 Hz, 1H), 7.76 (t, *J* = 8.2 Hz, 1H), 7.56 (t, *J* = 7.2 Hz, 1H), 2.66 (s, 3H); ^{13}C NMR (101 MHz, $CDCl_3$) δ 197.9, 155.9, 148.3, 143.8, 137.4, 137.0, 130.0, 129.9, 128.8, 127.7, 127.5, 127.4, 126.8, 118.9, 26.8. MS (EI, m/z): 247.26 $[M]^+$.

(16) ethyl 4-(quinolin-2-yl)benzoate (C16). CAS: 1079883-97-8.

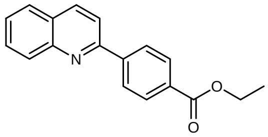

Using general procedure I. White solid (35 mg, 42%), m.p.: 84.5-85.9 °C; TCL (petroleum ether : ethyl acetate = 20 : 1): R_f = 0.30; ^{1}H NMR (400 MHz, $CDCl_3$) δ 8.27-8.20 (m, 6H), 7.89 (d, *J* = 8.5 Hz, 1H), 7.84 (d, *J* = 7.4 Hz, 1H), 7.76 (t, *J* = 8.4 Hz, 1H), 7.56 (t, *J* = 7.6 Hz, 1H), 4.44 (q, *J* = 7.1 Hz, 2H), 1.45 (t, *J* = 7.1 Hz, 3H); ^{13}C NMR (101 MHz, $CDCl_3$) δ 166.4, 156.0, 148.3, 143.6, 137.0, 131.0, 130.0, 129.9, 127.5,

127.4, 126.7, 118.9, 61.1, 14.4. MS (EI, m/z): 277.27 $[M]^+$.

(17) 2-(4-(trifluoromethyl)phenyl)quinoline (C17). CAS: 506421-62-1.

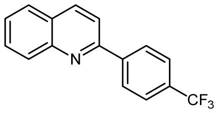

Using general procedure I. White solid (25 mg, 30%), m.p.: 126.0-127.5 °C; TCL (petroleum ether : ethyl acetate = 20 : 1): R_f = 0.60; ^{1}H NMR (500 MHz, $CDCl_3$) δ 8.26 (d, J = 8.1 Hz, 2H), 8.23 (d, J = 8.6 Hz, 1H), 8.18 (d, J = 8.5 Hz, 1H), 7.85 (d, J = 8.6 Hz, 1H), 7.83 (d, J = 9.2 Hz, 1H), 7.77-7.73 (m, 3H), 7.55 (t, J = 7.5 Hz, 1H); ^{13}C NMR (126 MHz, $CDCl_3$) δ 155.7, 148.3, 143.0, 137.1, 131.1 (d, J = 32.7 Hz), 129.9 (d, J = 15.3 Hz), 127.8, 127.5, 127.5, 126.9, 125.7 (q, J = 3.6 Hz), 125.3, 123.2, 118.8; ^{19}F NMR (471 MHz, $CDCl_3$) δ -62.55. MS (EI, m/z): 273.22 $[M]^+$.

(18) 2-(4-nitrophenyl)quinoline (C18). CAS: 64388-23-4.

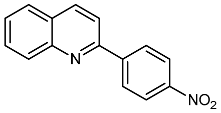

Using general procedure I. Yellow solid (23 mg, 30%), m.p.: 127.1-128.2 °C; TCL (petroleum ether : ethyl acetate = 20 : 1): R_f = 0.34; ^{1}H NMR (500 MHz, $CDCl_3$) δ 8.34-8.30 (m, 4H), 8.26 (d, J = 8.6 Hz, 1H), 8.17 (d, J = 8.5 Hz, 1H), 7.88 (d, J = 8.6 Hz, 1H), 7.85 (d, J = 8.1 Hz, 1H), 7.76 (t, J = 7.8 Hz, 1H), 7.58 (t, J = 7.5 Hz, 1H); ^{13}C NMR (126 MHz, $CDCl_3$) δ 154.5, 148.3, 148.3, 145.4, 137.3, 130.2, 130.0, 128.3, 127.6, 127.3, 124.0, 118.7. MS (EI, m/z): 250.22 $[M]^+$.

(19) 2-(3,4,5-trifluorophenyl)quinoline (C19). CAS: 1789717-78-7.

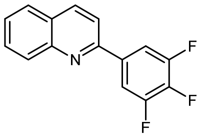

Using general procedure I. White solid (33 mg, 43%), m.p.: 115.8-116.7 °C; TCL (petroleum ether : ethyl acetate = 20 : 1): R_f = 0.66; ^{1}H NMR (500 MHz, $CDCl_3$) δ 8.15 (d, J = 8.6 Hz, 1H), 8.09 (d, J = 8.5 Hz, 1H), 7.79-7.75 (m, 3H), 7.71 (t, J = 7.7 Hz, 1H), 7.66 (d, J = 8.6 Hz, 1H), 7.52 (t, J = 7.5 Hz, 1H); ^{13}C NMR (126 MHz, CDCl3) δ 153.4, 152.6 (d, J = 4.0 Hz), 152.5 (d, J = 3.9 Hz), 141.6 (t, J = 15.5 Hz), 139.6 (t, J = 15.5 Hz), 137.2, 130.1, 129.7, 127.5, 127.4, 127.0, 117.7, 111.5 (d, J = 5.3 Hz), 111.3 (d, J = 5.3 Hz); ^{19}F NMR (471 MHz, $CDCl_3$) δ -133.82, -159.49. MS (EI, m/z): 259.20 $[M]^+$.

(20) 2-(benzo[*d*][1,3]dioxol-5-yl)quinoline (C20). CAS: 6808-65-7.

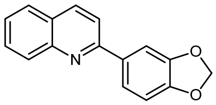

Using general procedure I. White solid (60 mg, 80%), m.p.: 90.0-91.5 °C; TCL (petroleum ether : ethyl acetate = 20 : 1): R_f = 0.38; ^{1}H NMR (400 MHz, $CDCl_3$) δ 8.14 (t, J = 9.6 Hz, 2H), 7.80-7.76 (m, 2H), 7.74 (s, 1H), 7.70 (t, J = 7.7 Hz, 1H), 7.65 (d, J = 8.1 Hz, 1H), 7.49 (t, J = 7.5 Hz, 1H), 6.94 (d, J = 8.1 Hz, 1H),

6.03 (s, 2H); ^{13}C NMR (101 MHz, $CDCl_3$) δ 156.7, 148.9, 148.4, 148.2, 136.7, 134.1, 129.7, 129.5, 127.4, 127.0, 126.1, 121.8, 118.6, 108.5, 108.0, 101.4. MS (EI, m/z): 249.20 $[M]^+$.

(21) 2-(6-methoxynaphthalen-2-yl)quinoline (C21). CAS: 860201-69-0.

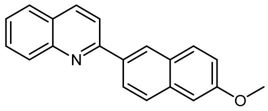

Using general procedure I. White solid (66 mg, 77%), m.p.: 170.0-171.5 °C; TCL (petroleum ether : ethyl acetate = 20 : 1): R_f = 0.22; ^{1}H NMR (400 MHz, $CDCl_3$) δ 8.55 (s, 1H), 8.33 (d, *J* = 8.6 Hz, 1H), 8.22 (t, *J* = 7.6 Hz, 2H), 8.00 (d, J = 8.6 Hz, 1H), 7.88 (d, *J* = 8.6 Hz, 2H), 7.83 (d, *J* = 7.1 Hz, 1H), 7.74 (t, *J* = 7.6 Hz, 1H), 7.52 (t, *J* = 7.4 Hz, 1H), 7.19 (t, *J* = 6.4 Hz, 2H), 3.95 (s, 3H); ^{13}C NMR (101 MHz, $CDCl_3$) δ 158.4, 157.3, 148.3, 136.8, 135.2, 134.8, 130.4, 129.7, 129.6, 129.0, 127.5, 127.4, 127.2, 127.0, 126.2, 125.6, 119.2, 119.0, 105.7, 55.4. MS (EI, m/z): 285.24 $[M]^+$.

(22) 2-(4-phenoxyphenyl)quinoline (C22). CAS: 860203-65-2.

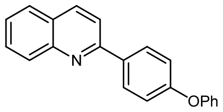

Using general procedure I. Yellow solid (67 mg, 75%), m.p.: 115.2-116.7 °C; TCL (petroleum ether : ethyl acetate = 20 : 1): R_f = 0.52; ^{1}H NMR (400 MHz, $CDCl_3$) δ 8.21-8.12 (m, 4H), 7.81 (t, *J* = 8.9 Hz, 2H), 7.71 (t, *J* = 7.7 Hz, 1H), 7.50 (t, *J* = 7.5 Hz, 1H), 7.36 (t, *J* = 7.4 Hz, 2H), 7.14 (d, *J* = 7.9 Hz, 3H), 7.08 (d, *J* = 7.9 Hz, 2H); ^{13}C NMR (101 MHz, $CDCl_3$) δ 158.7, 156.9, 156.7, 148.3, 136.9, 134.6, 129.9, 129.7, 129.6, 129.2, 127.5, 127.1, 126.2, 123.7, 119.3, 118.9, 118.7. MS (EI, m/z): 297.25 $[M]^+$.

(23) *N*,*N*-diphenyl-4-(quinolin-2-yl)aniline (C23). CAS: 1263145-46-5.

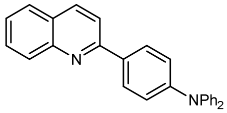

Using general procedure I. Yellow solid (67 mg, 60%), m.p.: 169.0-170.5 °C; TCL (petroleum ether : ethyl acetate = 20 : 1): R_f = 0.52; ^{1}H NMR (400 MHz, $CDCl_3$) δ 8.20 (t, *J* = 7.9 Hz, 2H), 8.09 (d, *J* = 8.2 Hz, 2H), 7.85 (t, *J* = 8.3 Hz, 2H), 7.74 (t, *J* = 7.5 Hz, 1H), 7.53 (t, *J* = 7.3 Hz, 1H), 7.33 (t, *J* = 7.5 Hz, 4H), 7.26-7.19 (m, 6H), 7.11 (t, *J* = 7.2 Hz, 2H); ^{13}C NMR (101 MHz, $CDCl_3$) δ 157.0, 149.1, 148.3, 147.5, 136.7, 133.2, 129.6, 129.6, 129.4, 128.5, 127.5, 127.0, 126.0, 124.9, 123.4, 123.2, 118.7. MS (EI, m/z): 372.29 $[M]^+$.

(24) 2-(1-methyl-1H-indol-5-yl)quinoline (C24). CAS: 1246092-04-5.

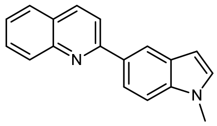

Using general procedure I. Yellow solid (46 mg, 60%), m.p.: 168.7-168.5 °C; TCL (petroleum ether : ethyl acetate = 20 : 1): R_f = 0.65; ^{1}H NMR (400 MHz, $CDCl_3$) δ 8.50 (d, *J* = 1.3 Hz, 1H), 8.25 (d, *J* = 8.5 Hz,

1H), 8.22-8.20 (m, 1H), 8.19-8.18 (m, 1H), 7.99 (d, J = 8.6 Hz, 1H), 7.84 (d, J = 8.1 Hz, 1H), 7.76 (t, J = 7.7 Hz, 1H), 7.53 (t, J = 6.9 Hz, 1H), 7.48 (d, J = 8.6 Hz, 1H), 7.12 (d, J = 3.1 Hz, 1H), 6.66 (d, J = 3.7 Hz, 1H), 3.84 (s, 3H); ^{13}C NMR (101 MHz, $CDCl_3$) δ 158.7, 148.4, 137.6, 136.4, 131.3, 129.7, 129.6, 129.4, 129.0, 127.5, 126.9, 125.7, 121.6, 120.6, 119.3, 109.6, 102.1, 33.0. MS (EI, m/z): 258.26 $[M]^+$.

(25) 2-(pyridin-4-yl)quinoline (C25). CAS: 52089-02-8.

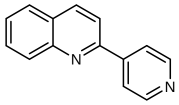

Using general procedure I. White solid (25 mg, 40%), m.p.: 96.7-97.8 °C; TCL (petroleum ether : ethyl acetate = 5 : 1): R_f = 0.27; ^{1}H NMR (400 MHz, $CDCl_3$) δ 8.78 (d, J = 6.1 Hz, 2H), 8.26 (d, J = 8.6 Hz, 1H), 8.20 (d, J = 8.5 Hz, 1H), 8.06 (d, J = 6.1 Hz, 2H), 7.88 (d, J = 8.6 Hz, 1H), 7.85 (d, J = 8.2 Hz, 1H), 7.77 (t, J = 7.7 Hz, 1H), 7.58 (t, J = 7.5 Hz, 1H); ^{13}C NMR (101 MHz, $CDCl_3$) δ 154.4, 150.5, 148.3, 146.6, 137.3, 130.1, 123.0, 127.8, 127.6, 127.2, 121.6, 118.4. MS (EI, m/z): 206.21 $[M]^+$.

(26) 2-(pyridin-3-yl)quinoline (C26). CAS: 6294-65-1.

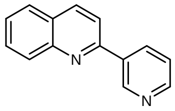

Using general procedure I. Yellow oil liquid (28 mg, 45%); TCL (petroleum ether : ethyl acetate = 5 : 1): R_f = 0.25; ^{1}H NMR (400 MHz, $CDCl_3$) δ 9.36 (s, 1H), 8.69 (d, J = 4.6 Hz, 1H), 8.47 (d, J = 9.3 Hz, 1H), 8.20 (d, J = 8.5 Hz, 1H), 8.16 (d, J = 8.5 Hz, 1H), 7.81 (t, J = 7.4 Hz, 2H), 7.73 (t, J = 7.7 Hz, 1H), 7.53 (t, J = 7.5 Hz, 1H), 7.44-7.40 (m, 1H); ^{13}C NMR (101 MHz, $CDCl_3$) δ 154.5, 150.2, 148.8, 148.3, 137.1, 135.1, 134.9, 130.0, 129.7, 127.6, 127.3, 126.8, 123.7, 118.5. MS (EI, m/z): 206.22 $[M]^+$.

(27) 2-(furan-3-yl)quinoline (C27). CAS: 1438894-27-9.

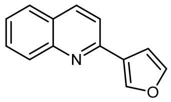

Using general procedure I. Black solid (26 mg, 45%), m.p.: 56.5-57.8 °C; TCL (petroleum ether : ethyl acetate = 20 : 1): R_f = 0.40; ^{1}H NMR (400 MHz, $CDCl_3$) δ 8.17 (s, 1H), 8.15 (d, J = 8.6 Hz, 1H), 8.11 (d, J = 8.5 Hz, 1H), 7.80 (d, J = 8.2 Hz, 1H), 7.72 (t, J = 7.7 Hz, 1H), 7.62 (d, J = 8.5 Hz, 1H), 7.57 (s, 1H), 7.51 (t, J = 7.8 Hz, 1H), 7.14 (s, 1H); ^{13}C NMR (101 MHz, $CDCl_3$) δ 151.7, 148.3, 144.0, 142.1, 136.5, 129.7, 129.3, 127.7, 127.5, 127.1, 126.0, 119.0, 109.1. MS (EI, m/z): 195.18 $[M]^+$.

(28) 2-(thiophen-3-yl)quinoline (C28). CAS: 104967-53-5.

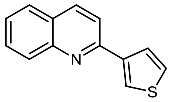

Using general procedure I. Yellow solid (26 mg, 41%), m.p.: 133.5-134.8 °C; TCL (petroleum ether : ethyl acetate = 20 : 1): R_f = 0.40; ^{1}H NMR (400 MHz, $CDCl_3$) δ 8.17 (d, J = 8.5 Hz, 1H), 8.13 (d, J = 8.6 Hz, 1H), 8.06 (s, 1H), 7.92 (d, J = 5.0 Hz, 1H), 7.79 (d, J = 8.2 Hz, 1H), 7.76-7.71 (m, 2H), 7.52 (t, J = 7.5 Hz,

1H), 7.48-7.44 (m, 1H); ^{13}C NMR (101 MHz, $CDCl_3$) δ 153.3, 148.3, 142.7, 136.7, 129.7, 129.5, 127.5, 127.1, 126.9, 126.4, 126.1, 124.7, 119.1. MS (EI, m/z): 211.16 $[M]^+$.

(29) 4-methyl-2-(*p*-tolyl)quinoline (C29). CAS: 148336-20-3.

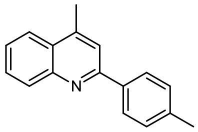

Using general procedure I. White solid (45 mg, 65%), m.p.: 54.4-55.9 °C; TCL (petroleum ether : ethyl acetate = 20 : 1): R_f = 0.65; ^{1}H NMR (400 MHz, $CDCl_3$) δ 8.21 (d, *J* = 8.4 Hz, 1H), 8.09 (d, *J* = 7.9 Hz, 2H), 8.01 (d, *J* = 8.2 Hz, 1H), 7.74 (d, *J* = 12.0 Hz, 2H), 7.56 (t, *J* = 7.5 Hz, 1H), 7.36 (d, *J* = 7.8 Hz, 2H), 2.78 (s, 3H), 2.47 (s, 3H); ^{13}C NMR (101 MHz, CDCl3) δ 156.0, 147.1, 143.7, 138.2, 135.9, 129.1, 128.5, 128.2, 126.4, 126.2, 124.8, 122.6, 118.6, 20.3, 18.0. MS (EI, m/z): 233.25 $[M]^+$.

(30) 6-methyl-2-(*p*-tolyl)quinoline (C30). CAS: 118714-26-4.

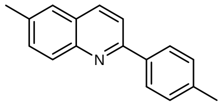

Using general procedure I. White solid (50 mg, 71%), m.p.: 131.0-132.5 °C; TCL (petroleum ether : ethyl acetate = 20 : 1): R_f = 0.65; ^{1}H NMR (400 MHz, $CDCl_3$) δ 8.08 (d, *J* = 8.2 Hz, 2H), 8.04 (d, *J* = 7.8 Hz, 2H), 7.79 (d, *J* = 8.6 Hz, 1H), 7.53 (d, *J* = 9.2 Hz, 2H), 7.31 (d, *J* = 7.9 Hz, 2H), 2.53 (s, 3H), 2.42 (s, 3H); ^{13}C NMR (101 MHz, $CDCl_3$) δ 155.4, 145.7, 138.2, 135.9, 135.0, 134.9, 130.9, 128.5, 128.2, 126.3, 126.1, 125.3, 117.8, 20.5, 20.3. MS (EI, m/z): 233.25 $[M]^+$.

(31) 7-methyl-2-(*p*-tolyl)quinoline (C31). CAS: 2413666-98-3.

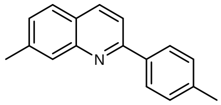

Using general procedure I. White solid (45 mg, 65%), m.p.: 123.0-124.4 °C; TCL (petroleum ether : ethyl acetate = 20 : 1): R_f = 0.63; ^{1}H NMR (400 MHz, $CDCl_3$) δ 8.03 (d, *J* = 8.6 Hz, 1H), 7.97 (d, *J* = 7.6 Hz, 2H), 7.87 (s, 1H), 7.69 (d, *J* = 8.6 Hz, 1H), 7.60 (d, *J* = 8.2 Hz, 1H), 7.26-7.22 (m, 3H), 2.48 (s, 3H), 2.34 (s, 3H); ^{13}C NMR (101 MHz, $CDCl_3$) δ 157.3, 148.4, 139.9, 139.3, 136.9, 136.4, 129.6, 128.7, 128.4, 127.4, 127.1, 125.2, 118.1, 21.9, 21.3. MS (EI, m/z): 233.25 $[M]^+$.

(32) 8-methyl-2-(*p*-tolyl)quinoline (C32).

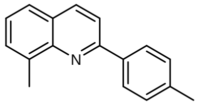

Using general procedure I. White solid (43 mg, 62%), m.p.: 75.5-76.9 °C; TCL (petroleum ether : ethyl acetate = 20 : 1): R_f = 0.68; ^{1}H NMR (400 MHz, $CDCl_3$) δ 8.15 (d, *J* = 8.2 Hz, 3H), 7.87 (d, *J* = 8.6 Hz, 1H), 7.64 (d, *J* = 8.0 Hz, 1H), 7.55 (d, *J* = 6.9 Hz, 1H), 7.39 (t, *J* = 7.5 Hz, 1H), 7.32 (d, *J* = 7.7 Hz, 2H), 2.89 (s, 3H), 2.43 (s, 3H); ^{13}C NMR (101 MHz, $CDCl_3$) δ 154.5, 146.1, 138.3, 136.5, 136.0, 135.8, 128.6,

128.5, 126.3, 126.0, 124.8, 124.3, 117.0, 20.3, 16.9. HRMS (ESI): Calcd. for $C_{17}H_{16}N$: 234.1277; found: 234.1272.

(33) 6-methoxy-2-(*p*-tolyl)quinoline (C33). CAS: 117839-37-9.

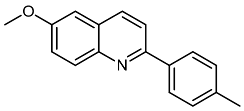

Using general procedure I. White solid (45 mg, 60%), m.p.: 140.8-141.9 °C; TCL (petroleum ether : ethyl acetate = 20 : 1): R_f = 0.60; ^{1}H NMR (400 MHz, $CDCl_3$) δ 8.11 (d, *J* = 2.8 Hz, 1H), 8.08 (d, *J* = 3.4 Hz, 2H), 8.06 (s, 1H), 7.83 (d, *J* = 8.6 Hz, 1H), 7.41 (d, *J* = 9.2 Hz, 1H), 7.35 (d, *J* = 7.9 Hz, 2H), 7.10 (d, *J* = 2.4 Hz, 1H), 3.96 (s, 3H), 2.46 (s, 3H); ^{13}C NMR (101 MHz, $CDCl_3$) δ 157.6, 155.0, 144.3, 139.0, 136.9, 135.5, 131.0, 129.5, 128.0, 127.2, 122.2, 119.1, 105.1, 55.6, 21.3. MS (EI, m/z): 249.25 $[M]^+$.

(34) 5-bromo-2-(*p*-tolyl)quinoline (C34).

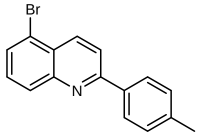

Using general procedure I. Yellow solid (64 mg, 71%), m.p.: 68.5-69.6 °C; TCL (petroleum ether : ethyl acetate = 20 : 1): R_f = 0.64; ^{1}H NMR (400 MHz, $CDCl_3$) δ 8.52 (d, *J* = 8.9 Hz, 1H), 8.11 (d, *J* = 8.5 Hz, 1H), 8.07 (d, *J* = 7.8 Hz, 2H), 7.91 (d, *J* = 8.9 Hz, 1H), 7.75 (d, *J* = 7.5 Hz, 1H), 7.53 (t, *J* = 8.0 Hz, 1H), 7.32 (d, *J* = 7.9 Hz, 2H), 2.43 (s, 3H); ^{13}C NMR (101 MHz, $CDCl_3$) δ 156.9, 148.0, 138.9, 135.1, 135.0, 128.7, 128.7, 128.6, 128.5, 126.5, 125.5, 120.7, 118.8, 20.3. HRMS (ESI): Calcd. for $C_{17}H_{16}N$: 298.0226; found: 298.0221.

(35) 6-fluoro-2-(*p*-tolyl)quinoline (C35) CAS: 1802383-39-6.

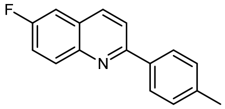

Using general procedure I. Yellow solid (50 mg, 70%), m.p.: 129.8-130.7 °C; TCL (petroleum ether : ethyl acetate = 20 : 1): R_f = 0.64; ^{1}H NMR (400 MHz, $CDCl_3$) δ 8.13 (dd, *J* = 9.2, 5.4 Hz, 1H), 8.09 (d, *J* = 8.7 Hz, 1H), 8.03 (d, *J* = 8.1 Hz, 2H), 7.83 (d, *J* = 8.7 Hz, 1H), 7.49-7.43 (m, 1H), 7.41-7.37 (m, 1H), 7.31 (d, *J* = 8.1 Hz, 2H), 2.42 (s, 3H); ^{13}C NMR (101 MHz, $CDCl_3$) δ 159.2 (d, *J* = 246 Hz), 155.7, 144.3, 138.5, 135.5, 134.9, 131.0 (d, *J* = 9 Hz), 128.6, 126.3, 118.8, 118.5, 109.4 (d, *J* = 21 Hz), 20.3; ^{19}F NMR (376 MHz, $CDCl_3$) δ -114.11. MS (EI, m/z): 237.22 $[M]^+$.

(36) 6-chloro-2-(*p*-tolyl)quinoline (C36). CAS: 1638845-00-7.

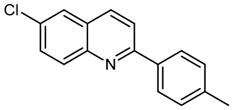

Using general procedure I. Yellow solid (54 mg, 71%), m.p.: 160.7-161.9 °C; TCL (petroleum ether : ethyl acetate = 20 : 1): R_f = 0.64; ^{1}H NMR (400 MHz, $CDCl_3$) δ 7.98-7.92 (m, 4H), 7.71 (d, *J* = 8.6 Hz, 1H),

7.63 (s, 1H), 7.52 (d, *J* = 9.0 Hz, 1H), 7.21 (d, *J* = 7.9 Hz, 2H), 2.32 (s, 3H); ^{13}C NMR (101 MHz, $CDCl_3$) δ 157.5, 146.7, 139.7, 136.4, 135.7, 131.7, 131.2, 130.5, 129.7, 127.7, 127.4, 126.1, 119.6, 21.4. MS (EI, m/z): 253.17 $[M]^+$.

(37) 6-bromo-2-(*p*-tolyl)quinoline (C37). CAS: 2378164-28-2.

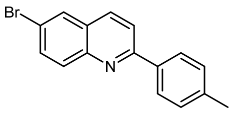

Using general procedure I. Yellow solid (58 mg, 65%), m.p.: 172.2-173.4 °C; TCL (petroleum ether : ethyl acetate = 20 : 1): R_f = 0.64; ^{1}H NMR (400 MHz, $CDCl_3$) δ 7.97-7.90 (m, 4H), 7.84 (s, 1H), 7.75 (d, *J* = 8.6 Hz, 1H), 7.67 (d, *J* = 8.9 Hz, 1H), 7.23 (d, *J* = 7.8 Hz, 2H), 2.34 (s, 3H); ^{13}C NMR (101 MHz, $CDCl_3$) δ 156.6, 145.8, 138.7, 135.3, 134.6, 132.0, 130.3, 128.6, 128.4, 127.1, 126.4, 118.8, 118.5, 20.3. MS (EI, m/z): 297.14 $[M]^+$.

(38) 6-iodo-2-(*p*-tolyl)quinoline (C38).

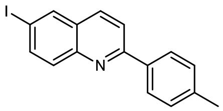

Using general procedure I. Brown solid (60 mg, 58%), m.p.: 180.3-181.5 °C; TCL (petroleum ether : ethyl acetate = 20 : 1): R_f = 0.64; ^{1}H NMR (400 MHz, $CDCl_3$) δ 8.17 (s, 1H), 8.06-8.01 (m, 3H), 7.92 (d, *J* = 10.4 Hz, 1H), 7.87 (d, *J* = 8.9 Hz, 1H), 7.83 (d, *J* = 8.7 Hz, 1H), 7.32 (d, *J* = 8.0 Hz, 2H), 2.43 (s, 3H); 13C NMR (101 MHz, CDCl3) δ 156.7, 146.2, 138.8, 137.2, 135.3, 135.1, 134.4, 130.3, 128.6, 127.7, 126.4, 118.4, 90.3, 20.3. HRMS (ESI): Calcd. for $C_{17}H_{16}N$: 346.0087; found: 346.0080.

(39) 8-chloro-2-(*p*-tolyl)quinoline (C39).

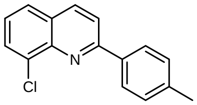

Using general procedure I. White solid (46 mg, 60%), m.p.: 95.0-96.9 °C; TCL (petroleum ether : ethyl acetate = 20 : 1): R_f = 0.58; ^{1}H NMR (400 MHz, $CDCl_3$) δ 8.19-8.13 (m, 3H), 7.90 (d, *J* = 8.6 Hz, 1H), 7.80 (d, *J* = 7.4 Hz, 1H), 7.68 (d, *J* = 8.1 Hz, 1H), 7.37 (t, *J* = 7.8 Hz, 1H), 7.32 (d, *J* = 7.6 Hz, 2H), 2.42 (s, 3H); ^{13}C NMR (101 MHz, $CDCl_3$) δ 157.5, 144.4, 140.0 137.1, 136.3, 133.9, 129.7, 129.7, 128.4, 127.6, 126.5, 125.9, 119.2, 21.4. HRMS (ESI): Calcd. for $C_{17}H_{16}N$: 254.0731; found: 254.0727.

(40) 8-bromo-2-(*p*-tolyl)quinoline (C40). CAS: 1345669-70-6.

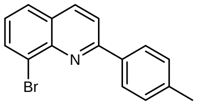

Using general procedure I. White solid (49 mg, 55%), m.p.: 98.5-99.8 °C; TCL (petroleum ether : ethyl acetate = 20 : 1): R_f = 0.57; ^{1}H NMR (400 MHz, $CDCl_3$) δ 8.20 (d, *J* = 7.6 Hz, 2H), 8.14 (d, *J* = 8.6 Hz, 1H), 7.91 (d, *J* = 8.6 Hz, 1H), 7.74 (d, *J* = 8.0 Hz, 1H), 7.35-7.30 (m, 3H), 2.43 (s, 3H); ^{13}C NMR (101 MHz, $CDCl_3$) δ 156.6, 144.0, 138.9, 136.1, 135.1, 132.2, 128.6, 127.3, 126.5, 126.3, 125.4, 124.4, 118.0,

20.4. MS (EI, m/z): 297.17 $[M]^+$.

(41) 6-bromo-4-chloro-2-(*p*-tolyl)quinoline (C41).

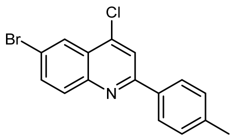

Using general procedure I. White solid (48 mg, 48%), m.p.: 127.4-128.7 °C; TCL (petroleum ether : ethyl acetate = 20 : 1): R_f = 0.65; ^{1}H NMR (400 MHz, $CDCl_3$) δ 8.37 (s, 1H), 8.04 (t, *J* = 8.8 Hz, 3H), 7.97 (s, 1H), 7.83 (d, *J* = 6.8 Hz, 1H), 7.35 (d, *J* = 8.0 Hz, 2H), 2.46 (s, 3H); ^{13}C NMR (101 MHz, $CDCl_3$) δ 157.5, 147.7, 141.8, 140.4, 135.3, 134.0, 131.6, 129.7, 127.3, 126.3, 126.3, 121.2, 119.5, 21.4. HRMS (ESI): Calcd. for $C_{17}H_{16}N$: 331.9836; found: 331.9830.

(42) 4,7-dichloro-2-(*p*-tolyl)quinoline (C42). CAS: 101726-90-3.

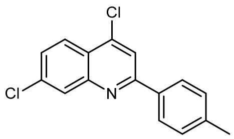

Using general procedure I. White solid (43 mg, 50%), m.p.: 120.0-121.4 °C; TCL (petroleum ether : ethyl acetate = 20 : 1): R_f = 0.70; ^{1}H NMR (400 MHz, $CDCl_3$) δ 8.18 (s, 1H), 8.14 (d, *J* = 8.9 Hz, 1H), 8.05 (d, *J* = 8.1 Hz, 2H), 7.94 (s, 1H), 7.55 (d, *J* = 8.9 Hz, 1H), 7.35 (d, *J* = 8.0 Hz, 2H), 2.46 (s, 3H); ^{13}C NMR (101 MHz, $CDCl_3$) δ 158.3, 149.4, 143.0, 140.4, 136.5, 135.3, 129.7, 128.8, 127.9, 127.4, 125.3, 123.7, 119.0, 21.4. MS (EI, m/z): 287.13 $[M]^+$.

(43) 4-chloro-6,7-dimethoxy-2-(*p*-tolyl)quinoline (C43).

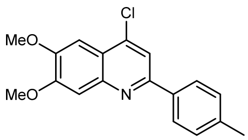

Yellow solid (40 mg, 42%), m.p.: 180.6-181.8 °C; TCL (petroleum ether : ethyl acetate = 10 : 1): R_f = 0.44; ^{1}H NMR (400 MHz, $CDCl_3$) δ 8.00 (d, *J* = 8.1 Hz, 2H), 7.82 (s, 1H), 7.50 (s, 1H), 7.41 (s, 1H), 7.33 (d, *J* = 8.0 Hz, 2H), 4.08 (s, 3H), 4.08 (s, 3H), 2.45 (s, 3H); ^{13}C NMR (101 MHz, $CDCl_3$) δ 155.4, 153.2, 150.4, 146.1, 140.9, 139.4, 136.1, 129.6, 127.1, 120.6, 117.2, 108.6, 101.7, 56.3, 56.3, 21.3. HRMS (ESI): Calcd. for $C_{17}H_{16}N$: 314.0942; found: 314.0936.

(44) methyl 2-(*p*-tolyl)quinoline-6-carboxylate (C44). CAS: 197730-08-8.

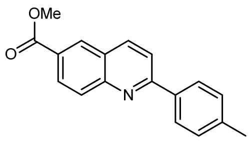

Using general procedure I. Brown solid (67 mg, 80%), m.p.: 174.9-175.8 °C; TCL (petroleum ether : ethyl acetate = 10 : 1): R_f = 0.40; ^{1}H NMR (400 MHz, $CDCl_3$) δ 8.57 (s, 1H), 8.31-8.27 (m, 2H), 8.19 (d, *J* = 8.8

Hz, 1H), 8.10 (d, J = 7.8 Hz, 2H), 7.92 (d, J = 8.6 Hz, 1H), 7.34 (d, J = 7.8 Hz, 2H), 3.99 (s, 3H), 2.44 (s, 3H); ^{13}C NMR (101 MHz, $CDCl_3$) δ 166.8, 159.3, 150.2, 140.2, 137.9, 136.2, 130.7, 129.8, 129.7, 129.2, 127.6, 127.5, 126.2, 119.5, 52.4, 21.4. MS (EI, m/z): 277.24 $[M]^+$.

(45) 5-nitro-2-(*p*-tolyl)quinoline (C45). CAS: 2253886-92-7.

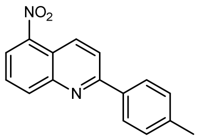

Using general procedure I. Yellow solid (59 mg, 75%), m.p.: 124.9-125.7 °C; TCL (petroleum ether : ethyl acetate = 20 : 1): R_f = 0.38; ^{1}H NMR (400 MHz, $CDCl_3$) δ 9.03 (d, J = 9.1 Hz, 1H), 8.45 (d, J = 8.5 Hz, 1H), 8.32 (d, J = 7.7 Hz, 1H), 8.10 (t, J = 8.2 Hz, 3H), 7.78 (t, J = 7.9 Hz, 1H), 7.36 (d, J = 7.6 Hz, 2H), 2.45 (s, 3H); ^{13}C NMR (101 MHz, $CDCl_3$) δ 158.3, 148.5, 145.5, 140.6, 136.7, 135.4, 132.6, 129.8, 127.6, 127.5, 124.0, 121.6, 120.0, 21.4. MS (EI, m/z): 264.23 $[M]^+$.

(46) 2-(*p*-tolyl)quinoxaline (C46). CAS: 17286-62-3.

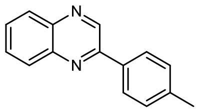

Using general procedure I. Yellow solid (33 mg, 50%), m.p.: 85.0-86.8 °C; TCL (petroleum ether : ethyl acetate = 20 : 1): R_f = 0.22; ^{1}H NMR (400 MHz, $CDCl_3$) δ 9.23 (s, 1H), 8.07-8.01 (m, 4H), 7.71-7.62 (m, 2H), 7.29 (d, J = 7.5 Hz, 2H), 2.37 (s, 3H); ^{13}C NMR (101 MHz, $CDCl_3$) δ 151.9, 143.3, 142.3, 141.4, 140.5, 134.0, 130.2, 129.9, 129.6, 129.3, 129.1, 127.5, 21.5. MS (EI, m/z): 220.22 $[M]^+$.

(47) 2-(*p*-tolyl)quinazoline (C47). CAS: 80089-59-4.

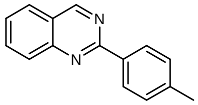

Using general procedure I. Yellow solid (34 mg, 52%), m.p.: 104.2-105.7 °C; TCL (petroleum ether : ethyl acetate = 10 : 1): R_f = 0.45; ^{1}H NMR (400 MHz, $CDCl_3$) δ 9.45 (s, 1H), 8.51 (d, J = 8.0 Hz, 2H), 8.07 (d, J = 8.4 Hz, 1H), 7.92-7.87 (m, 2H), 7.59 (t, J = 7.5 Hz, 1H), 7.34 (d, J = 7.9 Hz, 2H), 2.45 (s, 3H); ^{13}C NMR (101 MHz, $CDCl_3$) δ 161.2, 160.5, 150.8, 140.9, 135.4, 134.1, 129.4, 128.6, 128.5, 127.1, 127.1, 123.5, 21.5. MS (EI, m/z): 220.27 $[M]^+$.

(48) 2-(*p*-tolyl)-1,5-naphthyridine (C48). CAS: 2378283-13-5.

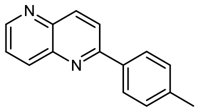

Using general procedure I. Brown solid (36 mg, 55%), m.p.: 118.2-119.7 °C; TCL (petroleum ether : ethyl acetate = 5 : 1): R_f = 0.38; ^{1}H NMR (400 MHz, $CDCl_3$) δ 8.94 (d, J = 2.8 Hz, 1H), 8.45 (d, J = 8.8 Hz, 2H), 8.09 (t, J = 8.9 Hz, 3H), 7.65 (d, J = 8.3 Hz, 1H), 7.35 (d, J = 7.7 Hz, 2H), 2.44 (s, 3H); ^{13}C NMR (101 MHz, $CDCl_3$) δ 158.1, 150.4, 143.7, 143.0, 140.1, 137.8, 136.1, 129.7, 128.7, 127.6, 124.5, 122.3, 21.4;

MS (EI, m/z): 220.26 $[M]^+$.

(49) 2-(*p*-tolyl)-1,8-naphthyridine (C49). CAS: 65182-53-8.

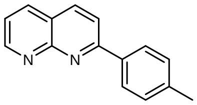

Using general procedure I. White solid (27 mg, 41%), m.p.: 148.5-149.7 °C; TCL (petroleum ether : ethyl acetate = 3 : 1): R_f = 0.20; ^{1}H NMR (400 MHz, $CDCl_3$) δ 9.12 (d, *J* = 6.1 Hz, 1H), 8.24 (d, *J* = 8.2 Hz, 2H), 8.21 (d, *J* = 8.6 Hz, 1H), 8.17 (d, *J* = 8.1 Hz, 1H), 7.99 (d, *J* = 8.5 Hz, 1H), 7.46-7.42 (m, 1H), 7.34 (d, *J* = 8.0 Hz, 2H), 2.44 (s, 3H); ^{13}C NMR (101 MHz, $CDCl_3$) δ 160.3, 156.2, 153.7, 140.4, 137.6, 136.7, 135.7, 129.6, 127.8, 121.6, 119.5, 21.4. MS (EI, m/z): 220.25 $[M]^+$.

(50) 8-(*p*-tolyl)imidazo[1,2-a]pyrazine (C50).

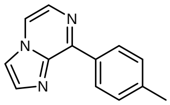

Under N_2 atmosphere, $[Cp^*IrCl_2]_2$ (1 mol%), *L*-proline (20 mol%), N-heteroarenes **A23** (0.3 mmol), arylboronic acids **B1** (0.36 mmol) and H_2O/1,4-dioxane (10/1, 1.5 mL) were introduced in a Schlenk tube (50 mL), successively. Then, the Schlenk tube was closed and the resulting mixture was stirred at 110 °C (oil bath temperature) for 30 h. After cooling down to room temperature, quenched with water, extracted with ethyl acetate (3×5 mL), and dried over anhydrous Na_2SO_4. The reaction mixture was concentrated by removing the solvent under vacuum, and the residue was purified by preparative TLC on silica, eluting with petroleum ether (60-90 °C) and ethyl acetate to give the desired product **C50**.

White solid (16 mg, 25%), m.p.: 91.2-92.5 °C; TCL (petroleum ether : ethyl acetate = 5 : 1): R_f = 0.48; ^{1}H NMR (500 MHz, $CDCl_3$) δ 8.57 (d, *J* = 8.2 Hz, 2H), 8.03 (d, *J* = 4.4 Hz, 1H), 7.96 (d, *J* = 4.5 Hz, 1H), 7.85 (s, 1H), 7.72 (s, 1H), 7.35 (d, *J* = 8.0 Hz, 2H), 2.44 (s, 3H); ^{13}C NMR (126 MHz, $CDCl_3$) δ 150.5, 140.6, 134.8, 134.5, 133.3, 129.8, 129.5, 129.2, 129.1, 128.0, 117.6, 113.7, 21.5. HRMS (ESI): Calcd. for $C_{13}H_{12}N_3$: 210.1026; found: 210.1025.

(51) 2-(*p*-tolyl)benzo[h]quinoline (C51). CAS: 912365-39-0.

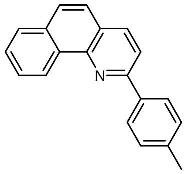

Using general procedure I. White solid (40 mg, 50%), m.p.: 70.5-71.4 °C; TCL (petroleum ether : ethyl acetate = 20 : 1): R_f = 0.30; ^{1}H NMR (400 MHz, $CDCl_3$) δ 9.49 (d, *J* = 8.1 Hz, 1H), 8.25 (d, *J* = 7.9 Hz, 2H), 8.20 (d, *J* = 8.4 Hz, 1H), 7.99 (d, *J* = 8.4 Hz, 1H), 7.90 (d, *J* = 7.7 Hz, 1H), 7.77 (t, *J* = 8.7 Hz, 2H), 7.73-7.68 (m, 2H), 7.36 (d, *J* = 7.9 Hz, 2H), 2.46 (s, 3H); ^{13}C NMR (101 MHz, $CDCl_3$) δ 155.6, 146.2, 139.3, 137.0, 136.5, 133.9, 131.9, 129.6, 128.1, 127.8, 127.3, 127.2, 126.8, 125.1, 125.0, 124.8, 118.7, 21.4. MS (EI, m/z): 269.26 $[M]^+$.

(52) 6-(*p*-tolyl)phenanthridine (C52). CAS: 47135-83-1.

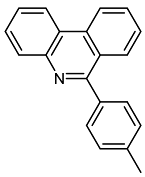

Using general procedure I. Yellow solid (39 mg, 48%), m.p.: 81.5-82.9 °C; TCL (petroleum ether : ethyl acetate = 20 : 1): $R_f = 0.37$; ^{1}H NMR (400 MHz, $CDCl_3$) δ 8.69 (d, J = 8.0 Hz, 1H), 8.61 (d, J = 7.9 Hz, 1H), 8.25 (d, J = 8.0 Hz, 1H), 8.14 (d, J = 8.2 Hz, 1H), 7.85 (t, J = 7.5 Hz, 1H), 7.75 (t, J = 7.5 Hz, 1H), 7.69-7.58 (m, 4H), 7.37 (d, J = 7.4 Hz, 2H), 2.48 (s, 3H); ^{13}C NMR (101 MHz, $CDCl_3$) δ 161.3, 143.7, 138.7, 136.8, 133.5, 130.6, 130.2, 129.7, 129.1, 129.1, 128.8, 127.1, 126.9, 125.3, 123.7, 122.2, 121.9, 21.4. MS (EI, m/z): 269.20 $[M]^+$.

(53) 5-(*p*-tolyl)thieno[3,2-b]pyridine (C53).

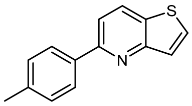

Under N_2 atmosphere, $[Cp^*IrCl_2]_2$ (1 mol%), *L*-proline (20 mol%), N-heteroarenes **A26** (0.3 mmol), arylboronic acids **B1** (0.36 mmol) and H_2O/1,4-dioxane (10/1, 1.5 mL) were introduced in a Schlenk tube (50 mL), successively. Then, the Schlenk tube was closed and the resulting mixture was stirred at 110 °C (oil bath temperature) for 30 h. After cooling down to room temperature, quenched with water, extracted with ethyl acetate (3×5 mL), and dried over anhydrous Na_2SO_4. The reaction mixture was concentrated by removing the solvent under vacuum, and the residue was purified by preparative TLC on silica, eluting with petroleum ether (60-90 °C) and ethyl acetate to give the desired product **C53**.

Yellow solid (22 mg, 32%), m.p.: 83.0-84.6 °C; TCL (petroleum ether : ethyl acetate = 10 : 1): $R_f = 0.55$; ^{1}H NMR (500 MHz, $CDCl_3$) δ 8.20 (d, J = 8.5 Hz, 1H), 7.96 (d, J = 8.1 Hz, 2H), 7.74 (d, J = 5.5 Hz, 1H), 7.67 (d, J = 8.5 Hz, 1H), 7.61 (d, J = 5.5 Hz, 1H), 7.30 (d, J = 8.0 Hz, 2H), 2.42 (s, 3H); ^{13}C NMR (126 MHz, $CDCl_3$) δ 156.3, 155.6, 138.9, 136.9, 131.3, 130.9, 130.7, 129.6, 127.2, 125.5, 116.3, 21.3. HRMS (ESI): Calcd. for $C_{14}H_{12}NS$: 226.0685; found: 226.0683.

(54) 2-(*p*-tolyl)pyrimidine (C54). CAS: 77232-13-4.

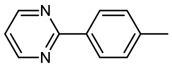

Under N_2 atmosphere, $[Cp^*IrCl_2]_2$ (1 mol%), *L*-proline (20 mol%), N-heteroarenes **A27** (0.3 mmol), arylboronic acids **B1** (0.36 mmol) and H_2O/1,4-dioxane (10/1, 1.5 mL) were introduced in a Schlenk tube (50 mL), successively. Then, the Schlenk tube was closed and the resulting mixture was stirred at 110 °C (oil bath temperature) for 48 h. After cooling down to room temperature, quenched with water, extracted with ethyl acetate (3×5 mL), and dried over anhydrous Na_2SO_4. The reaction mixture was concentrated by removing the solvent under vacuum, and the residue was purified by preparative TLC on silica, eluting with petroleum ether (60-90 °C) and ethyl acetate to give the desired product **C54**.

White solid (13 mg, 25%), m.p.: 86.8-87.4 °C; TCL (petroleum ether : ethyl acetate = 20 : 1): $R_f = 0.48$; ^{1}H NMR (400 MHz, $CDCl_3$) δ 8.81 (d, J = 4.8 Hz, 2H), 8.36 (d, J = 8.0 Hz, 2H), 7.33 (d, J = 8.0 Hz, 2H), 7.18 (t, J = 4.8 Hz, 1H), 2.45 (s, 3H); ^{13}C NMR (101 MHz, $CDCl_3$) δ 164.9, 157.2, 141.1, 134.9, 129.4, 128.1,

118.8, 21.5. MS (EI, m/z): 170.24 $[M]^+$.

(55) 2-(*p*-tolyl)pyrazine (C55). CAS: 108030-80-4.

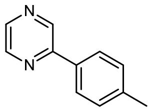

Under N_2 atmosphere, $[Cp^*IrCl_2]_2$ (1 mol%), *L*-proline (20 mol%), N-heteroarenes **A28** (0.3 mmol), arylboronic acids **B1** (0.36 mmol) and H_2O/1,4-dioxane (10/1, 1.5 mL) were introduced in a Schlenk tube (50 mL), successively. Then, the Schlenk tube was closed and the resulting mixture was stirred at 110 °C (oil bath temperature) for 48 h. After cooling down to room temperature, quenched with water, extracted with ethyl acetate (3×5 mL), and dried over anhydrous Na_2SO_4. The reaction mixture was concentrated by removing the solvent under vacuum, and the residue was purified by preparative TLC on silica, eluting with petroleum ether (60-90 °C) and ethyl acetate to give the desired product **C55**.

Colorless oil liquid (17 mg, 33%); TCL (petroleum ether : ethyl acetate = 20 : 1): R_f = 0.45; ^{1}H NMR (500 MHz, $CDCl_3$) δ 9.01 (s, 1H), 8.60 (d, *J* = 2.3 Hz, 1H), 8.47 (d, *J* = 2.3 Hz, 1H), 7.92 (d, *J* = 8.1 Hz, 2H), 7.32 (d, *J* = 8.0 Hz, 2H), 2.42 (s, 3H); ^{13}C NMR (126 MHz, $CDCl_3$) δ 152.9, 144.1, 142.6, 142.0, 140.2, 133.6, 129.8, 126.8, 21.4. MS (EI, m/z): 170.20 $[M]^+$.

(56) 2-(*p*-tolyl)pyridine (C56). CAS: 4467-06-5.

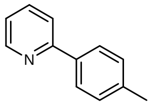

Under N_2 atmosphere, (Cp*)Ir(*L*-Pro)Cl (1 mol%), *L*-proline (20 mol%), CF_3COOH (20 mol%), N-heteroarenes **A29** (0.3 mmol), arylboronic acids **B1** (0.36 mmol) and H_2O/1,4-dioxane (10/1, 1.5 mL) were introduced in a Schlenk tube (50 mL), successively. Then, the Schlenk tube was closed and the resulting mixture was stirred at 130 °C (oil bath temperature) for 48 h. After cooling down to room temperature, quenched with water, extracted with ethyl acetate (3×5 mL), and dried over anhydrous Na_2SO_4. The reaction mixture was concentrated by removing the solvent under vacuum, and the residue was purified by preparative TLC on silica, eluting with petroleum ether (60-90 °C) and ethyl acetate to give the desired product **C56**.

Using general procedure I. Colorless oil liquid (10 mg, 20%); TCL (petroleum ether : ethyl acetate = 20 : 1): R_f = 0.53; ^{1}H NMR (500 MHz, $CDCl_3$) δ 8.67 (d, *J* = 4.6 Hz, 1H), 7.89 (d, *J* = 8.1 Hz, 2H), 7.71 (t, *J* = 9.3 Hz, 2H), 7.28 (d, *J* = 8.0 Hz, 2H), 7.20 (t, *J* = 6.6 Hz, 1H), 2.41 (s, 3H); ^{13}C NMR (126 MHz, $CDCl_3$) δ 157.5, 149.6, 139.0, 136.7, 129.5, 126.8, 121.8, 120.3, 21.3. MS (EI, m/z): 169.07 $[M]^+$.

(57) 2-phenyl-6-(*p*-tolyl)pyridine (C57). CAS: 1286679-11-5.

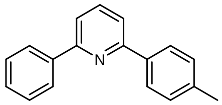

Under N_2 atmosphere, (Cp*)Ir(*L*-Pro)Cl (1 mol%), *L*-proline (20 mol%), CF_3COOH (20 mol%), N-heteroarenes **A30** (0.3 mmol), arylboronic acids **B1** (0.36 mmol) and H_2O/1,4-dioxane (10/1, 1.5 mL) were introduced in a Schlenk tube (50 mL), successively. Then, the Schlenk tube was closed and the resulting mixture was stirred at 130 °C (oil bath temperature) for 48 h. After cooling down to room

temperature, quenched with water, extracted with ethyl acetate (3×5 mL), and dried over anhydrous Na_2SO_4. The reaction mixture was concentrated by removing the solvent under vacuum, and the residue was purified by preparative TLC on silica, eluting with petroleum ether (60-90 °C) and ethyl acetate to give the desired product **C57**.

White solid (23 mg, 31%), m.p.: 84.5-85.2 °C; TCL (petroleum ether : ethyl acetate = 20 : 1): R_f = 0.65; ^{1}H NMR (500 MHz, $CDCl_3$) δ 8.07 (d, J = 7.4 Hz, 2H), 7.98 (d, J = 8.0 Hz, 2H), 7.71 (t, J = 7.8 Hz, 1H), 7.58 (d, J = 8.0 Hz, 2H), 7.42 (t, J = 7.5 Hz, 2H), 7.34 (t, J = 7.3 Hz, 1H), 7.22 (d, J = 7.9 Hz, 2H), 2.34 (s, 3H); ^{13}C NMR (126 MHz, $CDCl_3$) δ 156.9, 156.8, 139.6, 139.0, 137.4, 136.7, 129.4, 128.9, 128.7, 127.0, 126.9, 118.3, 21.3. MS (EI, m/z): 245.11 $[M]^+$.

(58) 4-phenyl-2-(*p*-tolyl)pyridine (C58). CAS: 262362-61-8.

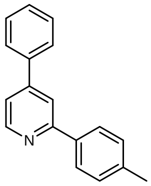

Under N_2 atmosphere, (Cp*)Ir(*L*-Pro)Cl (1 mol%), *L*-proline (20 mol%), CF_3COOH (20 mol%), N-heteroarenes **A31** (0.3 mmol), arylboronic acids **B1** (0.36 mmol) and H_2O/1,4-dioxane (10/1, 1.5 mL) were introduced in a Schlenk tube (50 mL), successively. Then, the Schlenk tube was closed and the resulting mixture was stirred at 130 °C (oil bath temperature) for 48 h. After cooling down to room temperature, quenched with water, extracted with ethyl acetate (3×5 mL), and dried over anhydrous Na_2SO_4. The reaction mixture was concentrated by removing the solvent under vacuum, and the residue was purified by preparative TLC on silica, eluting with petroleum ether (60-90 °C) and ethyl acetate to give the desired product **C58**.

Colorless oil liquid (19 mg, 26%); TCL (petroleum ether : ethyl acetate = 20 : 1): R_f = 0.55; ^{1}H NMR (500 MHz, $CDCl_3$) δ 8.71 (d, J = 5.0 Hz, 1H), 7.95 (d, J = 7.8 Hz, 2H), 7.90 (s, 1H), 7.69 (d, J = 7.4 Hz, 2H), 7.50 (t, J = 7.3 Hz, 2H), 7.46 (d, J = 7.0 Hz, 1H), 7.42 (d, J = 4.9 Hz, 1H), 7.30 (d, J = 7.8 Hz, 2H), 2.42 (s, 3H); ^{13}C NMR (126 MHz, $CDCl_3$) δ 158.1, 150.0, 149.3, 139.1, 138.7, 136.7, 129.5, 129.1, 129.0, 127.1, 126.9, 120.0, 118.5, 21.3. MS (EI, m/z): 245.11 $[M]^+$.

(59) 2-(pyridin-2-yl)quinoline (C59). CAS: 7491-86-3.

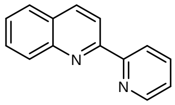

Using general procedure I. White solid (20 mg, 32%), m.p.: 94.6-95.8 °C; TCL (petroleum ether : ethyl acetate = 5 : 1): R_f = 0.62; ^{1}H NMR (500 MHz, $CDCl_3$) δ 8.74 (d, J = 4.6 Hz, 1H), 8.66 (d, J = 8.0 Hz, 1H), 8.56 (d, J = 8.6 Hz, 1H), 8.29 (d, J = 8.6 Hz, 1H), 8.18 (d, J = 8.5 Hz, 1H), 7.90-7.85 (m, 2H), 7.74 (t, J = 7.7 Hz, 1H), 7.56 (t, J = 7.5 Hz, 1H), 7.38-7.35 (m, 1H); ^{13}C NMR (126 MHz, $CDCl_3$) δ 156.4, 156.2, 149.2, 147.9, 137.0, 136.8, 129.8, 129.6, 128.3, 127.6, 126.8, 124.0, 121.9, 119.0. MS (EI, m/z): 206.22 $[M]^+$.

(60) 2,8'-biquinoline (C60). CAS: 2376100-65-9.

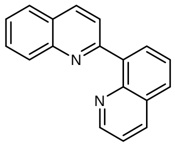

Using general procedure I. White solid (39 mg, 51%), m.p.: 120.9-121.8 °C; TCL (petroleum ether : ethyl acetate = 5 : 1): R_f = 0.40; ^{1}H NMR (400 MHz, $CDCl_3$) δ 8.97 (d, *J* = 4.1 Hz, 1H), 8.27-8.23 (m, 3H), 8.22 (d, *J* = 8.5 Hz, 1H), 8.14 (d, *J* = 8.5 Hz, 1H), 7.92 (d, *J* = 8.1 Hz, 1H), 7.89 (d, *J* = 8.2 Hz, 1H), 7.77-7.73 (m, 1H), 7.71 (t, *J* = 6.1 Hz, 1H), 7.56 (t, *J* = 7.5 Hz, 1H), 7.44-7.40 (m, 1H); ^{13}C NMR (101 MHz, $CDCl_3$) δ 158.3, 150.4, 148.5, 146.1, 139.5, 136.4, 134.8, 131.5, 129.7, 129.3, 129.1, 128.6, 127.6, 127.4, 126.7, 126.4, 125.0, 121.2. MS (EI, m/z): 256.22 $[M]^+$.

(61) 6-methyl-2,8'-biquinoline (C61).

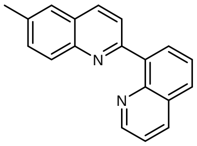

Using general procedure I. Colorless oil liquid (36 mg, 45%); TCL (petroleum ether : ethyl acetate = 5 : 1): R_f = 0.39; ^{1}H NMR (400 MHz, $CDCl_3$) δ 8.96 (d, *J* = 5.5 Hz, 1H), 8.24 (d, *J* = 7.1 Hz, 1H), 8.20 (d, *J* = 8.3 Hz, 1H), 8.16 (d, *J* = 8.8 Hz, 2H), 8.11 (d, *J* = 8.5 Hz, 1H), 7.90 (d, *J* = 8.2 Hz, 1H), 7.70 (t, *J* = 7.6 Hz, 1H), 7.64 (s, 1H), 7.57 (d, *J* = 8.6 Hz, 1H), 7.42-7.38 (m, 1H), 2.56 (s, 3H); ^{13}C NMR (101 MHz, $CDCl_3$) δ 157.3, 150.3, 147.1, 146.1, 139.6, 136.4, 136.2, 134.3, 131.6, 131.5, 129.3, 129.0, 128.6, 127.4, 126.7, 126.4, 125.0, 121.1, 21.7. HRMS (ESI): Calcd. for $C_{17}H_{16}N$: 271.1230; found: 271.1224.

(62) 2-(pyridin-2-yl)quinoxaline (C62). CAS: 7755-91-1.

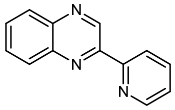

Using general procedure I. Yellow solid (22 mg, 35%), m.p.: 122.5-126.7 °C; TCL (petroleum ether : ethyl acetate = 10 : 1): R_f = 0.45; ^{1}H NMR (500 MHz, $CDCl_3$) δ 9.96 (s, 1H), 8.78 (d, *J* = 4.6 Hz, 1H), 8.59 (d, *J* = 7.9 Hz, 1H), 8.16 (d, *J* = 9.3 Hz, 2H), 7.89 (t, *J* = 7.7 Hz, 1H), 7.80-7.75 (m, 2H), 7.42-7.39 (m, 1H); ^{13}C NMR (126 MHz, $CDCl_3$) δ 154.5, 150.2, 149.4, 144.2, 142.6, 141.8, 137.1, 130.2, 130.1, 129.7, 129.3, 124.6, 122.1. MS (EI, m/z): 207.22 $[M]^+$.

(63) 2-(quinolin-8-yl)quinoxaline (C63). CAS: 7755-91-1.

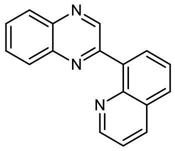

Using general procedure I. White solid (33 mg, 43%), m.p.: 182.5-183.9 °C; TCL (petroleum ether : ethyl acetate = 5 : 1): R_f = 0.35; ^{1}H NMR (400 MHz, $CDCl_3$) δ 9.64 (s, 1H), 8.97 (d, *J* = 4.0 Hz, 1H), 8.30 (d, *J* = 7.1 Hz, 1H), 8.26 (d, *J* = 8.3 Hz, 1H), 8.24-8.19 (m, 2H), 7.99 (d, *J* = 8.2 Hz, 1H), 7.81-7.78 (m, 2H),

7.77-7.73 (m, 1H), 7.50-7.46 (m, 1H); ^{13}C NMR (101 MHz, $CDCl_3$) δ 153.3, 150.7, 148.7, 145.9, 142.8, 141.4, 136.4, 136.4, 131.8, 130.0, 129.7, 129.6, 129.6, 129.2, 128.6, 126.8, 121.6. MS (EI, m/z): 257.21 $[M]^+$.

NMR spectra of the obtained compounds

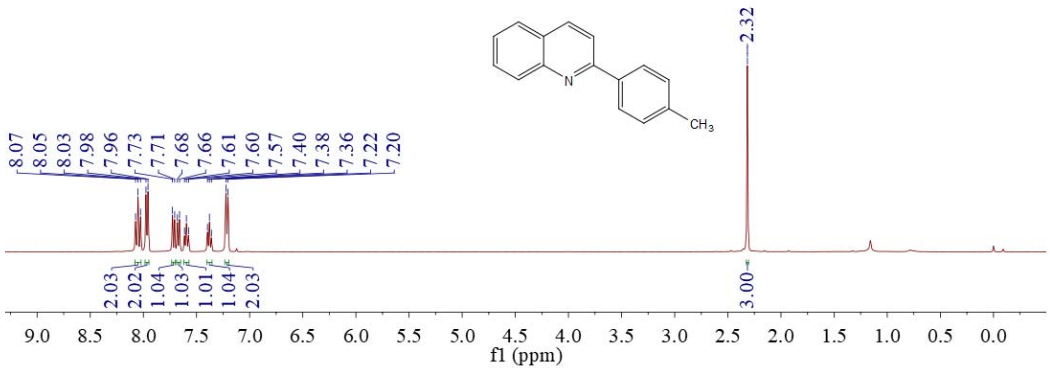

Supplementary Figure 7. ^{1}H-NMR (400 MHz, $CDCl_3$) spectrum of **C1**

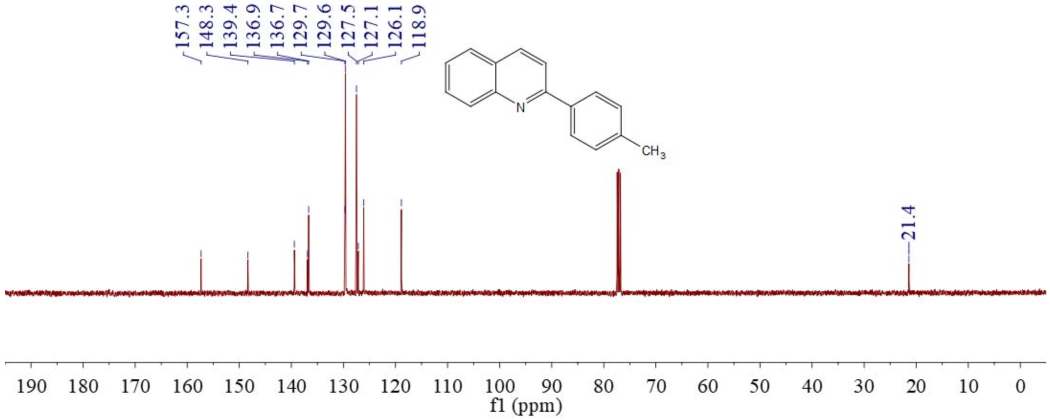

Supplementary Figure 8. ^{13}C-NMR (101 MHz, $CDCl_3$) spectrum of **C1**

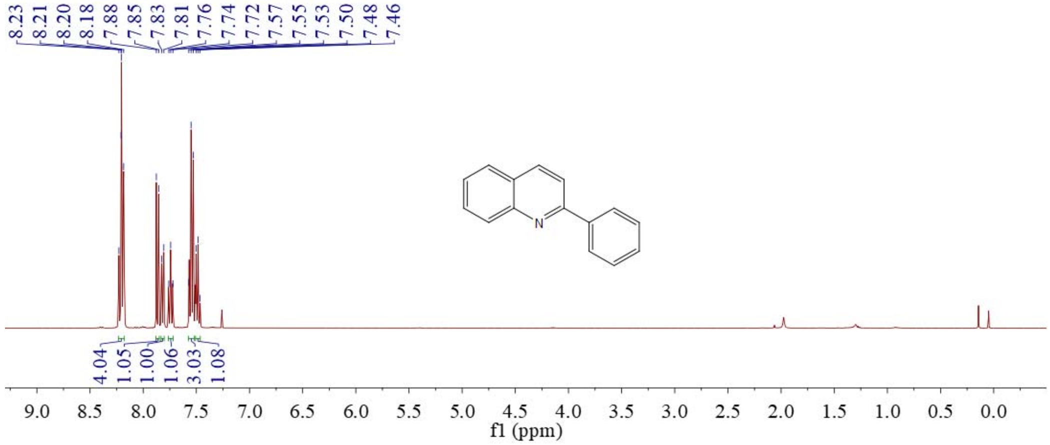

Supplementary Figure 9. ^{1}H-NMR (400 MHz, $CDCl_3$) spectrum of **C2**

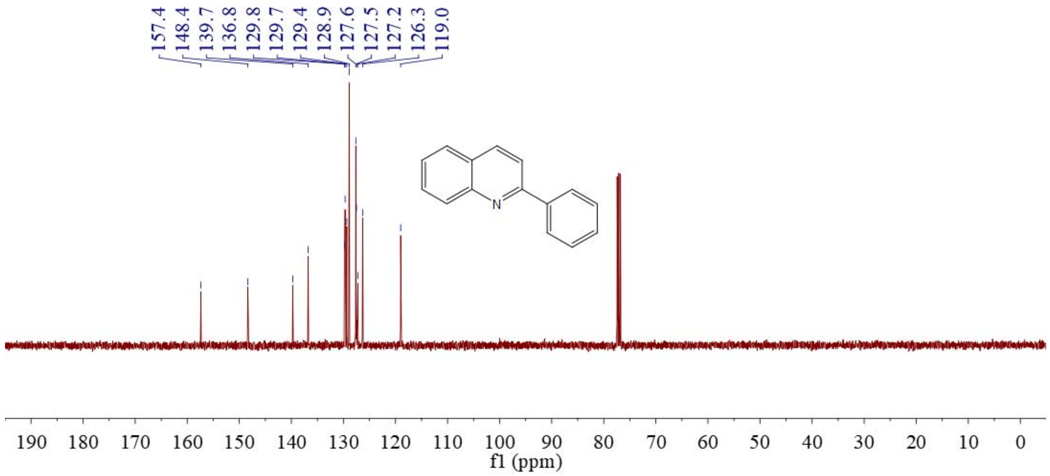

Supplementary Figure 10. ^{13}C-NMR (101 MHz, $CDCl_3$) spectrum of **C2**

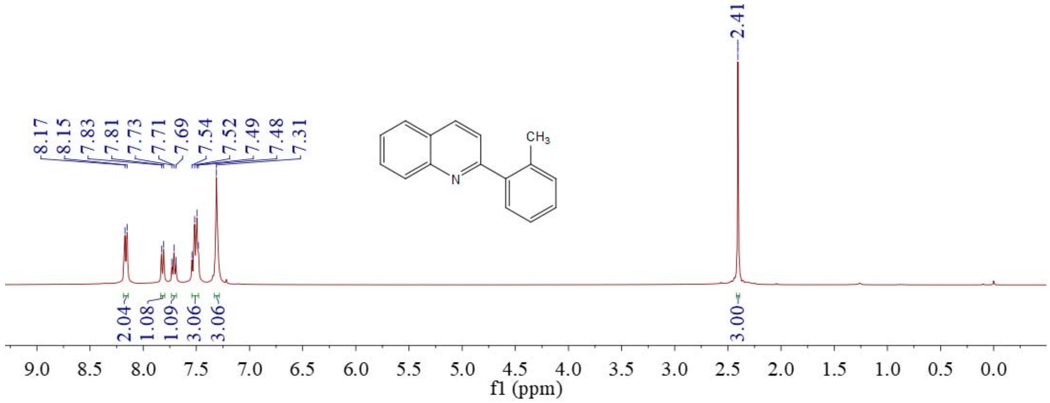

Supplementary Figure 11. ^{1}H-NMR (400 MHz, $CDCl_3$) spectrum of **C3**

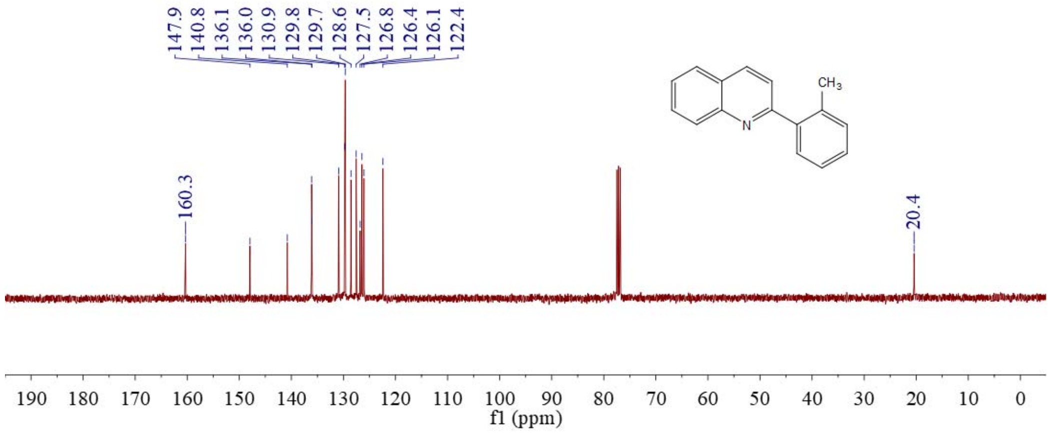

Supplementary Figure 12. ^{13}C-NMR (101 MHz, $CDCl_3$) spectrum of **C3**

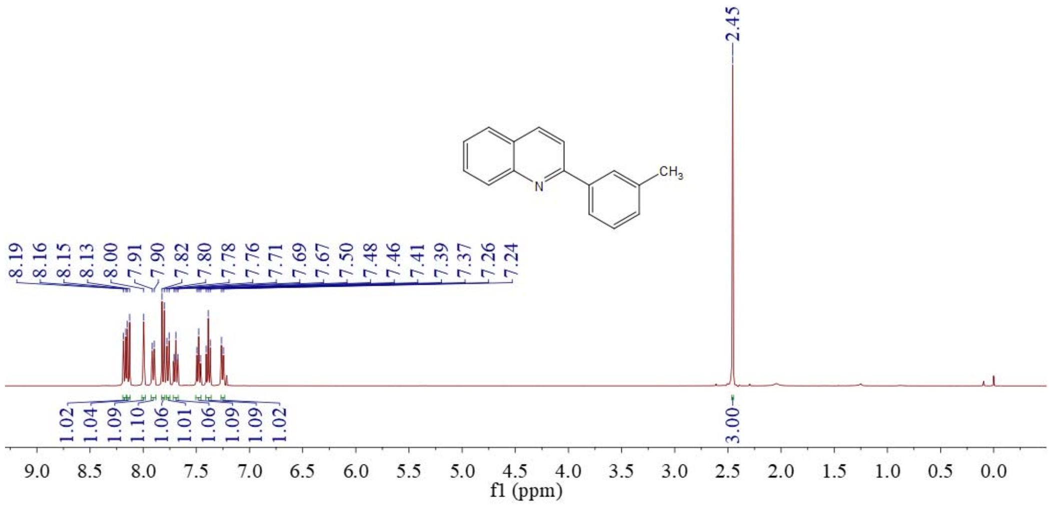

Supplementary Figure 13. ^{1}H-NMR (400 MHz, $CDCl_3$) spectrum of **C4**

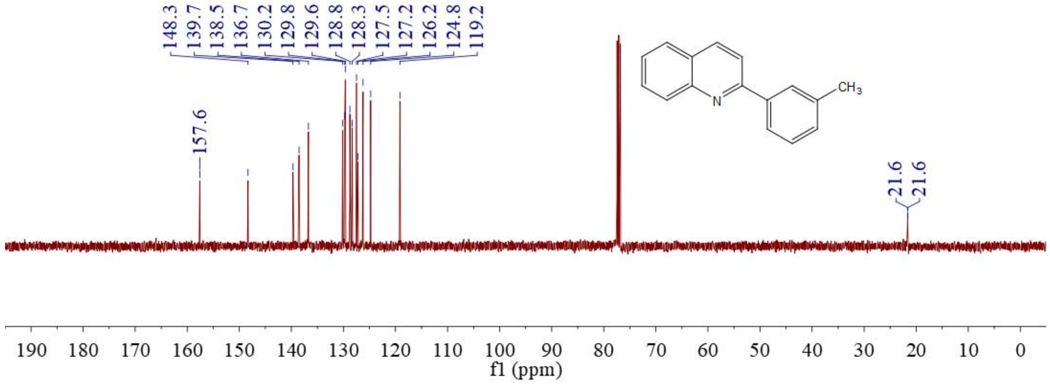

Supplementary Figure 14. ^{13}C-NMR (101 MHz, $CDCl_3$) spectrum of **C4**

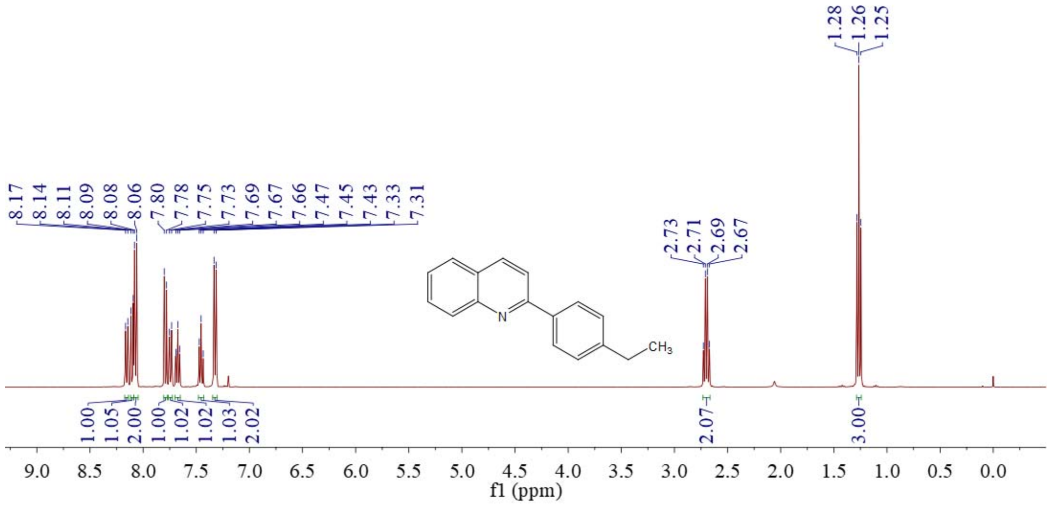

Supplementary Figure 15. ^{1}H-NMR (400 MHz, $CDCl_3$) spectrum of **C5**

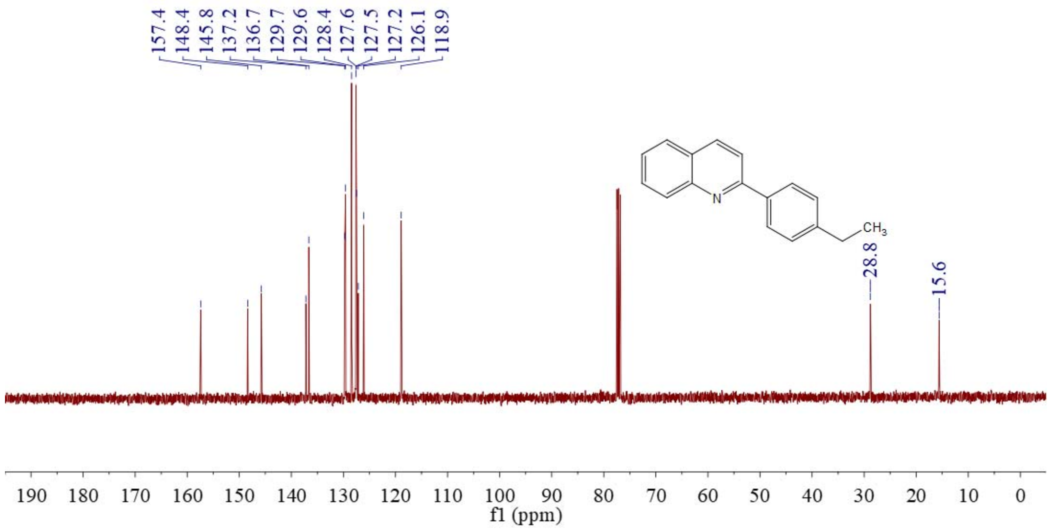

Supplementary Figure 16. ^{13}C-NMR (101 MHz, $CDCl_3$) spectrum of **C5**

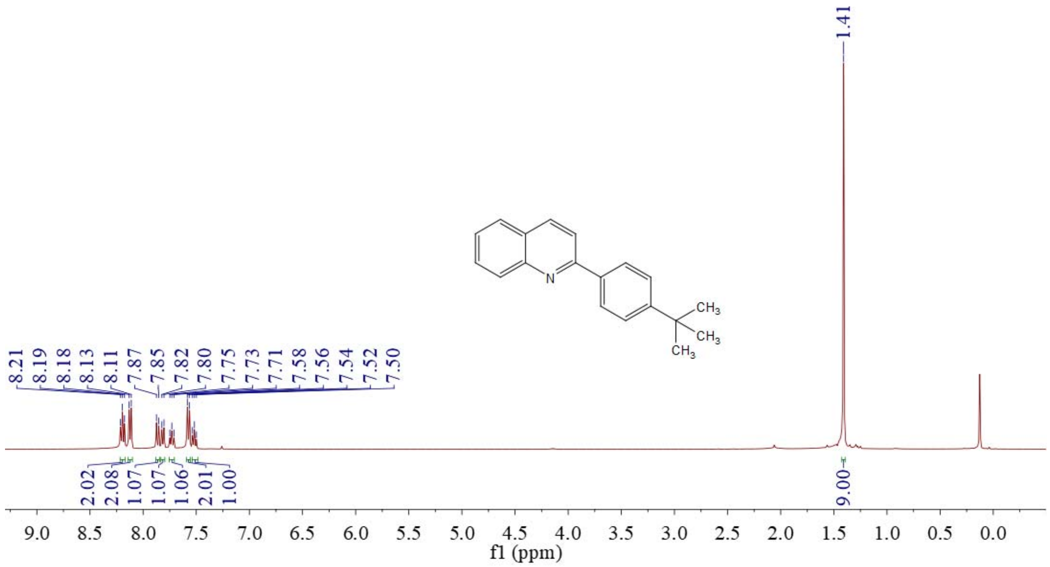

Supplementary Figure 17. ^{1}H-NMR (400 MHz, $CDCl_3$) spectrum of **C6**

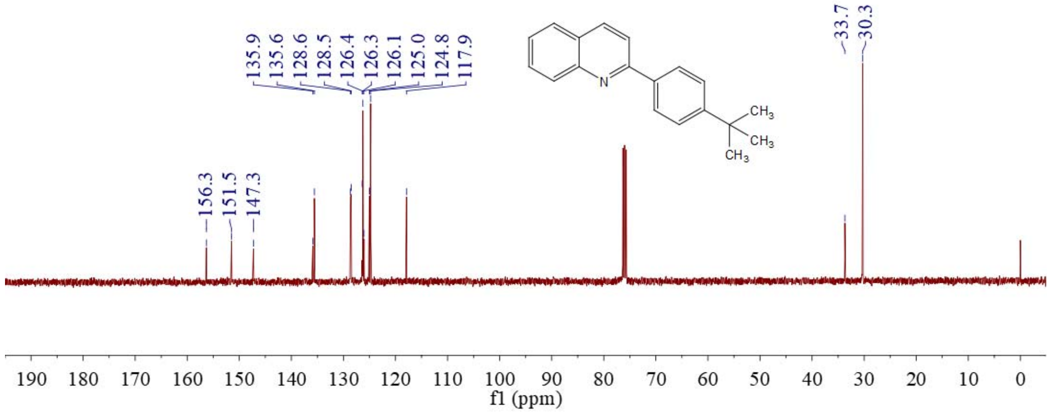

Supplementary Figure 18. ^{13}C-NMR (101 MHz, $CDCl_3$) spectrum of **C6**

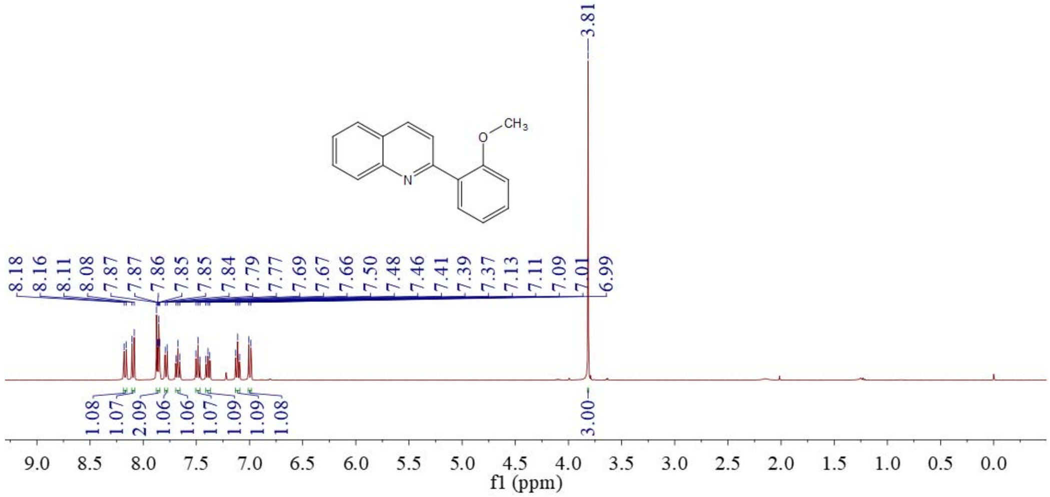

Supplementary Figure 19. ^{1}H-NMR (400 MHz, $CDCl_3$) spectrum of **C7**

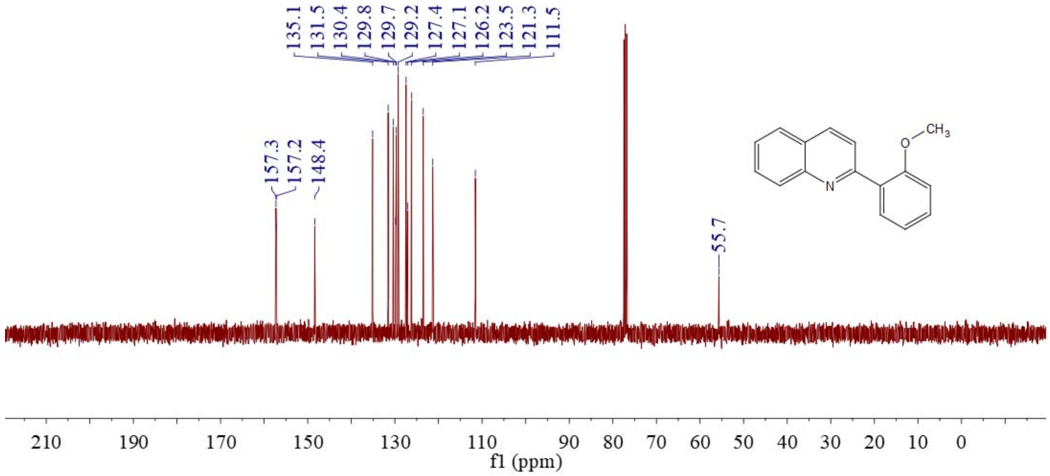

Supplementary Figure 20. ^{13}C-NMR (101 MHz, $CDCl_3$) spectrum of **C7**

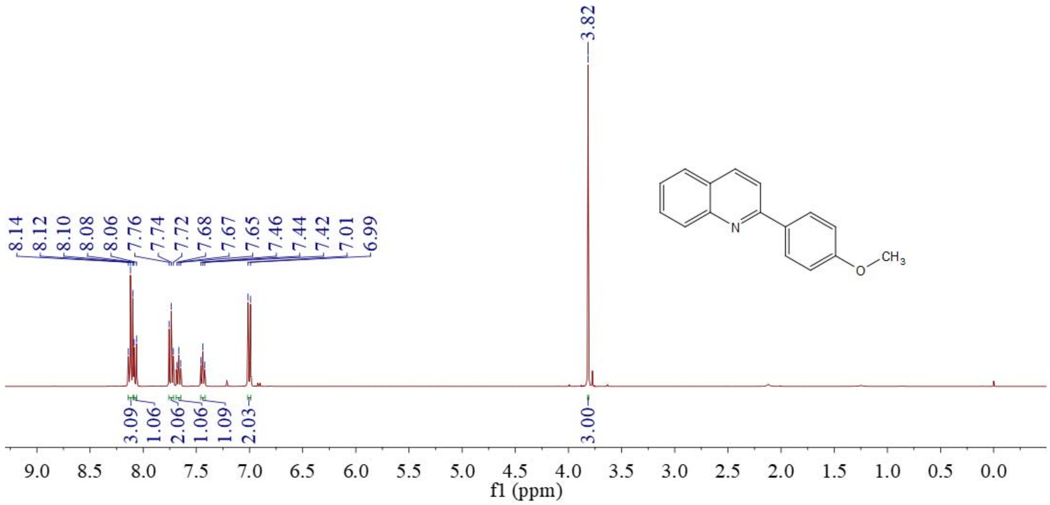

Supplementary Figure 21. ^{1}H-NMR (400 MHz, $CDCl_3$) spectrum of **C8**

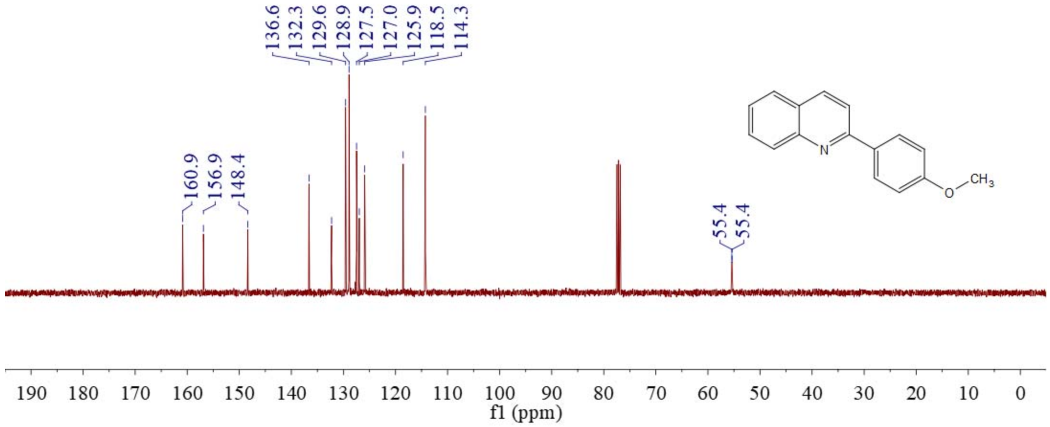

Supplementary Figure 22. ^{13}C-NMR (101 MHz, $CDCl_3$) spectrum of **C8**

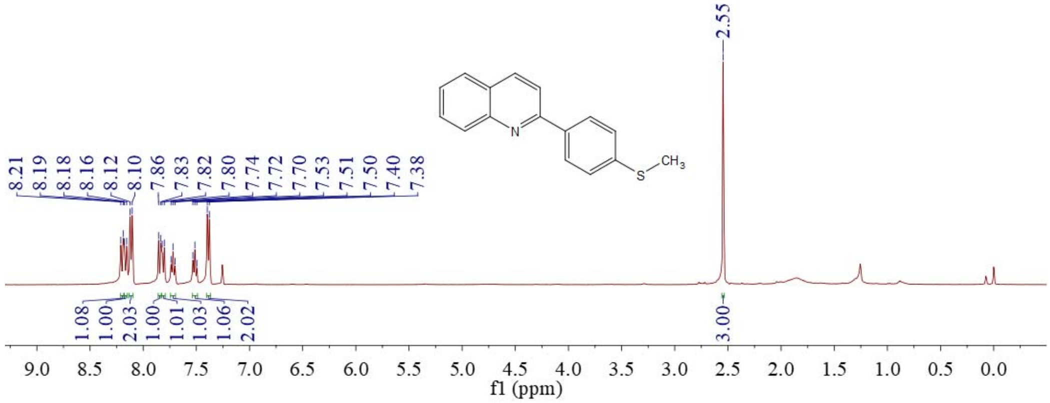

Supplementary Figure 23. ^{1}H-NMR (400 MHz, $CDCl_3$) spectrum of **C9**

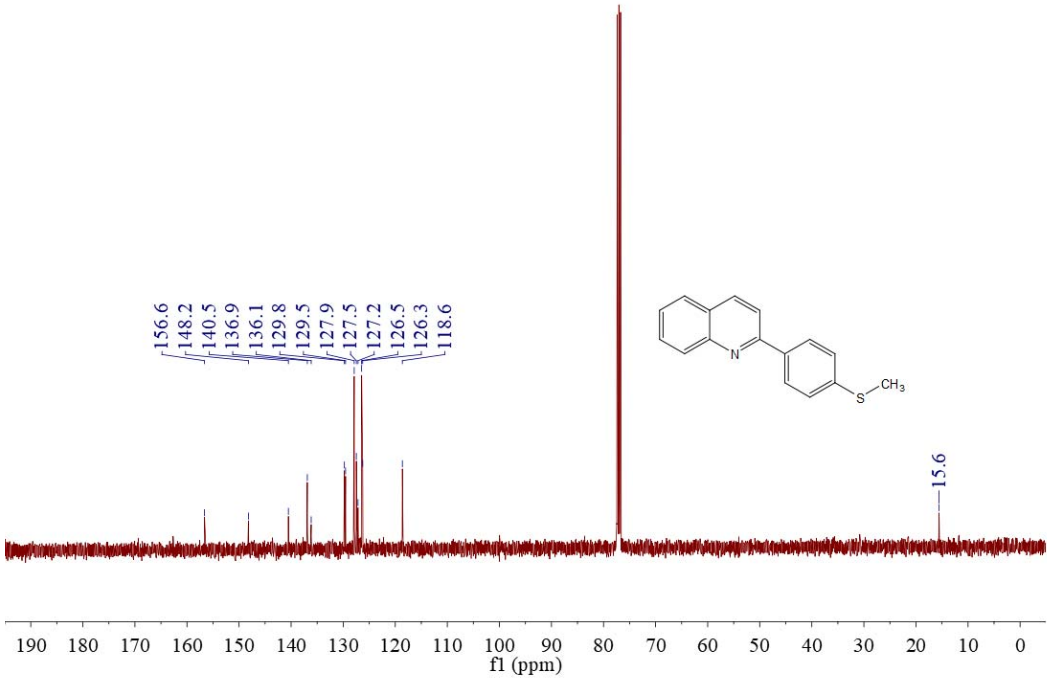

Supplementary Figure 24. ^{13}C-NMR (101 MHz, $CDCl_3$) spectrum of **C9**

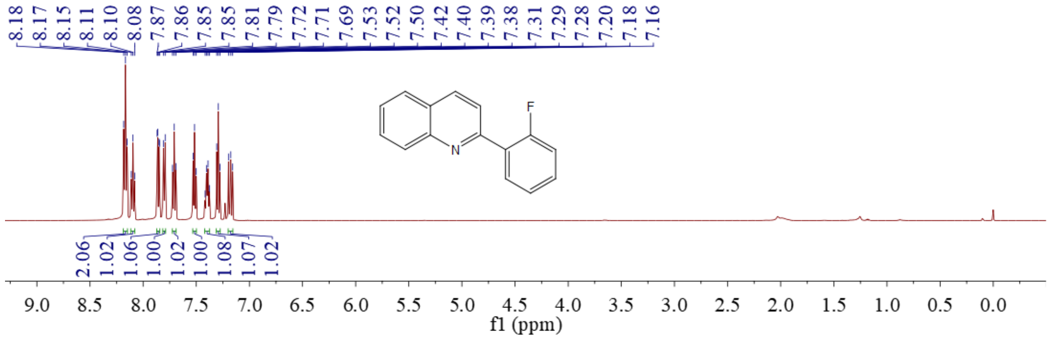

Supplementary Figure 25. ^{1}H-NMR (500 MHz, $CDCl_3$) spectrum of **C10**

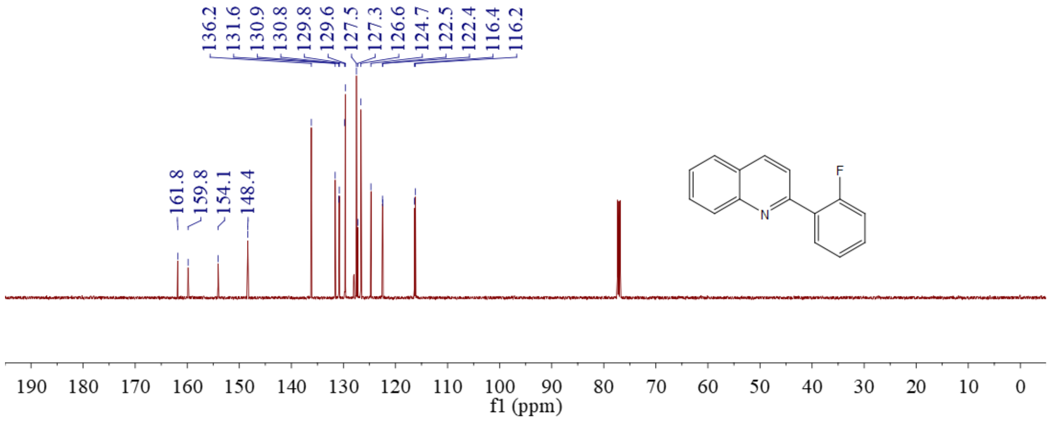

Supplementary Figure 26. ^{13}C-NMR (126 MHz, $CDCl_3$) spectrum of **C10**

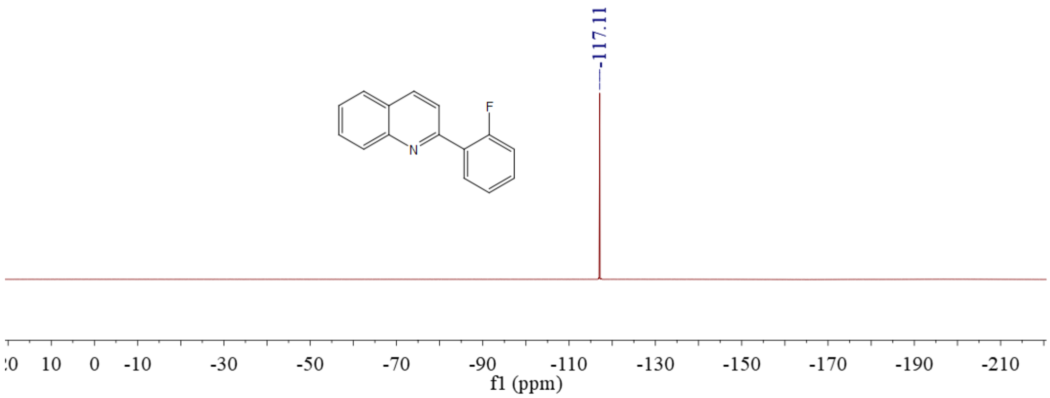

Supplementary Figure 27. ^{19}F-NMR (471 MHz, $CDCl_3$) spectrum of **C10**

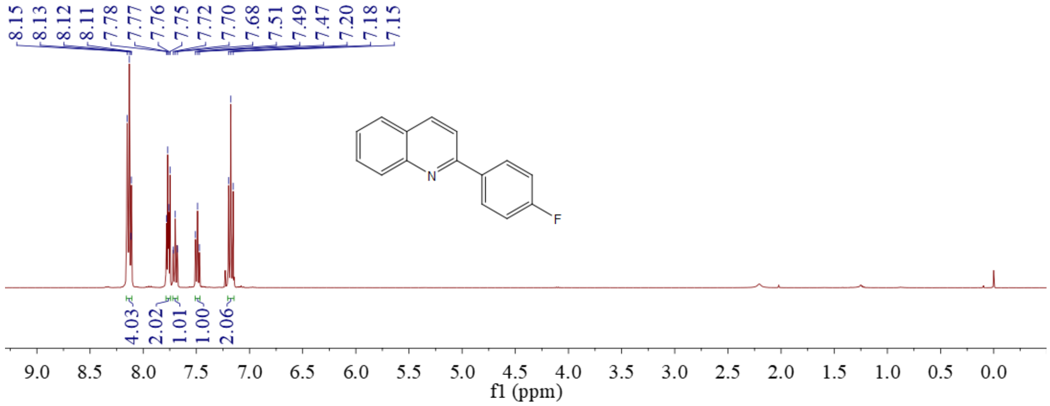

Supplementary Figure 28. ^{1}H-NMR (400 MHz, $CDCl_3$) spectrum of **C11**

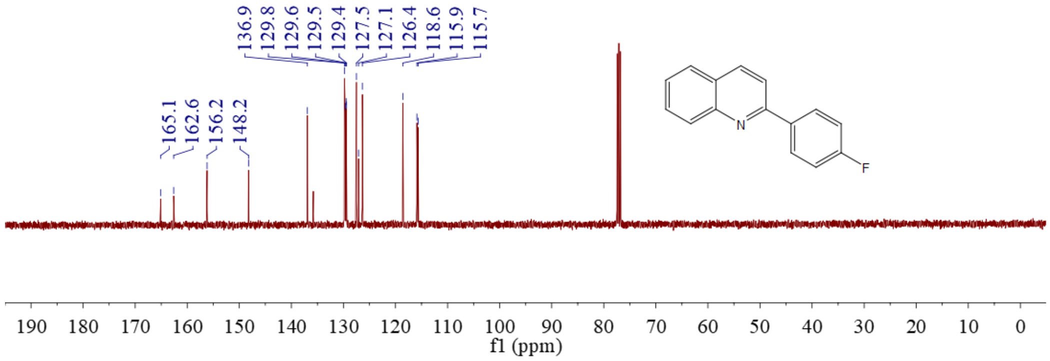

Supplementary Figure 29. ^{13}C-NMR (101 MHz, $CDCl_3$) spectrum of **C11**

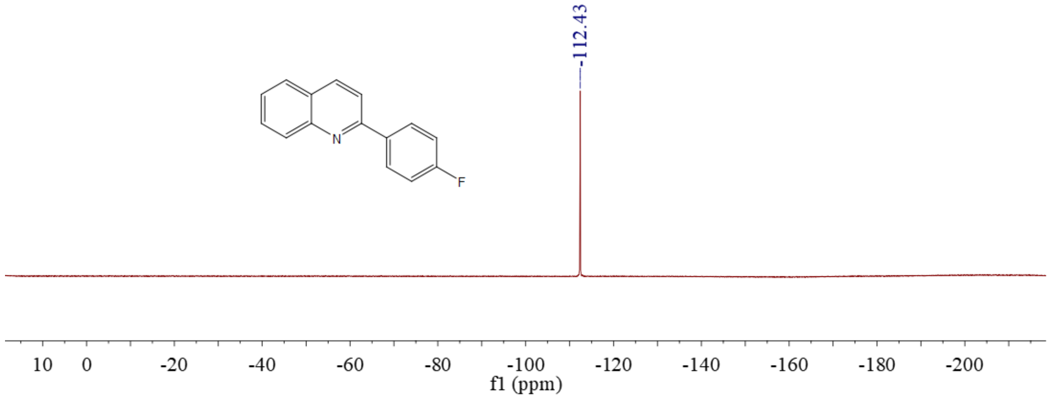

Supplementary Figure 30. ^{19}F-NMR (376 MHz, $CDCl_3$) spectrum of **C11**

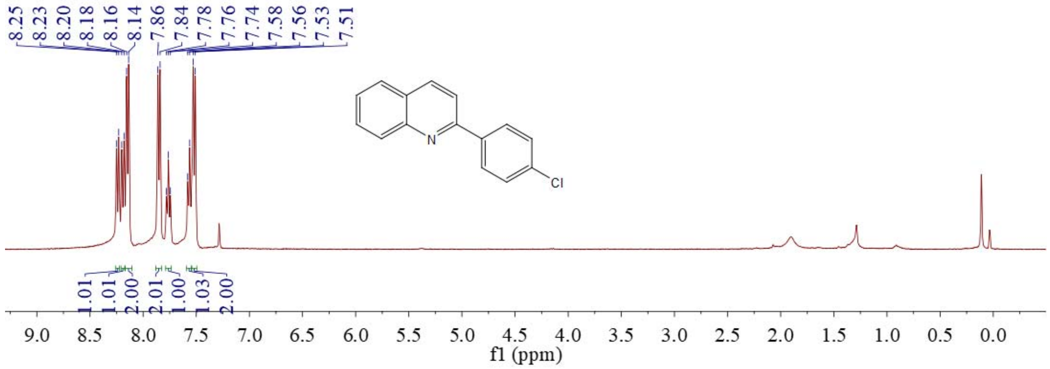

Supplementary Figure 31. ^{1}H-NMR (400 MHz, $CDCl_3$) spectrum of **C12**

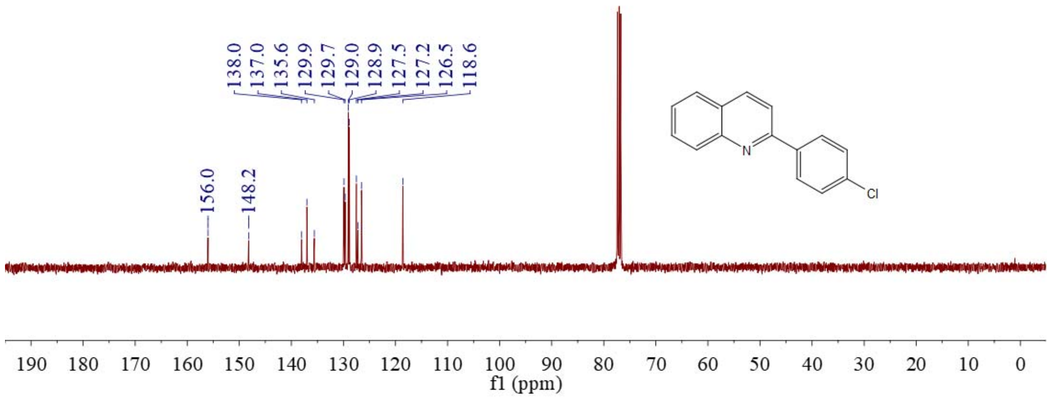

Supplementary Figure 32. ^{13}C-NMR (101 MHz, $CDCl_3$) spectrum of **C12**

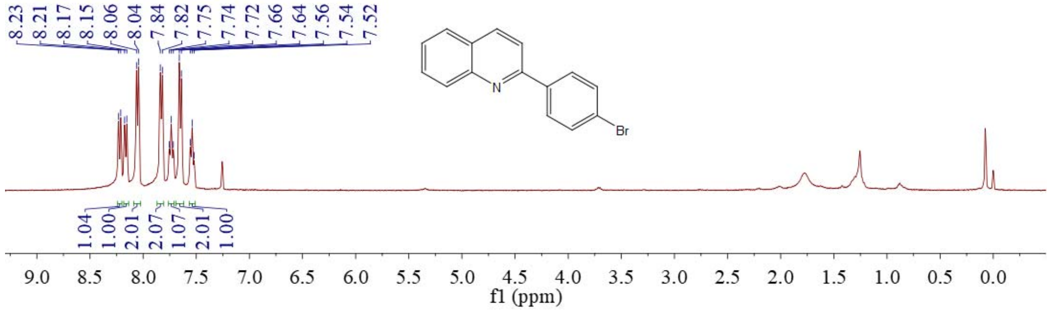

Supplementary Figure 33. ^{1}H-NMR (400 MHz, $CDCl_3$) spectrum of **C13**

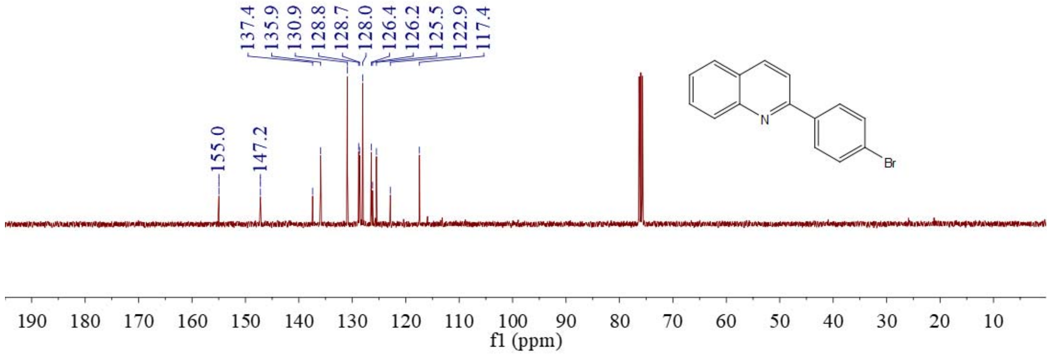

Supplementary Figure 34. ^{13}C-NMR (101 MHz, $CDCl_3$) spectrum of **C13**

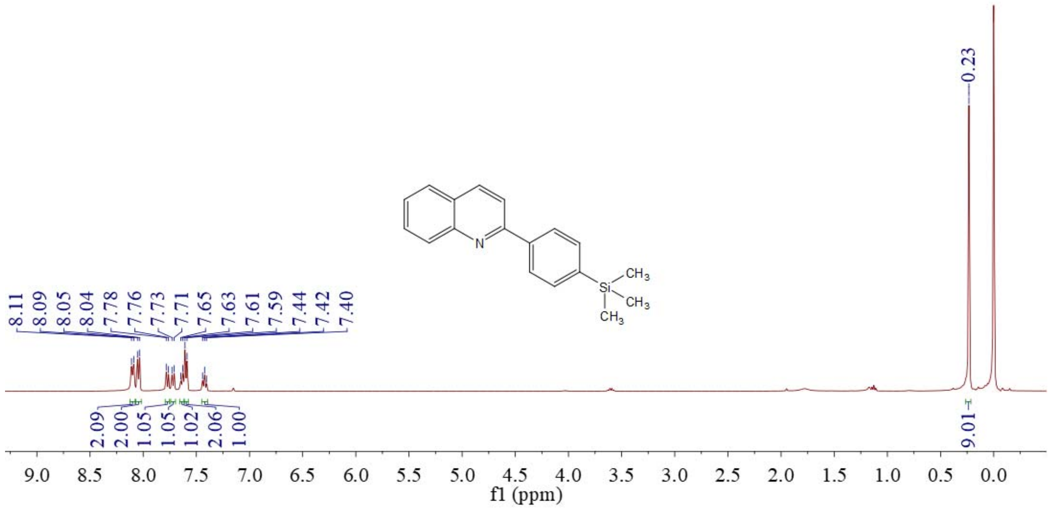

Supplementary Figure 35. ^{1}H-NMR (400 MHz, $CDCl_3$) spectrum of **C14**

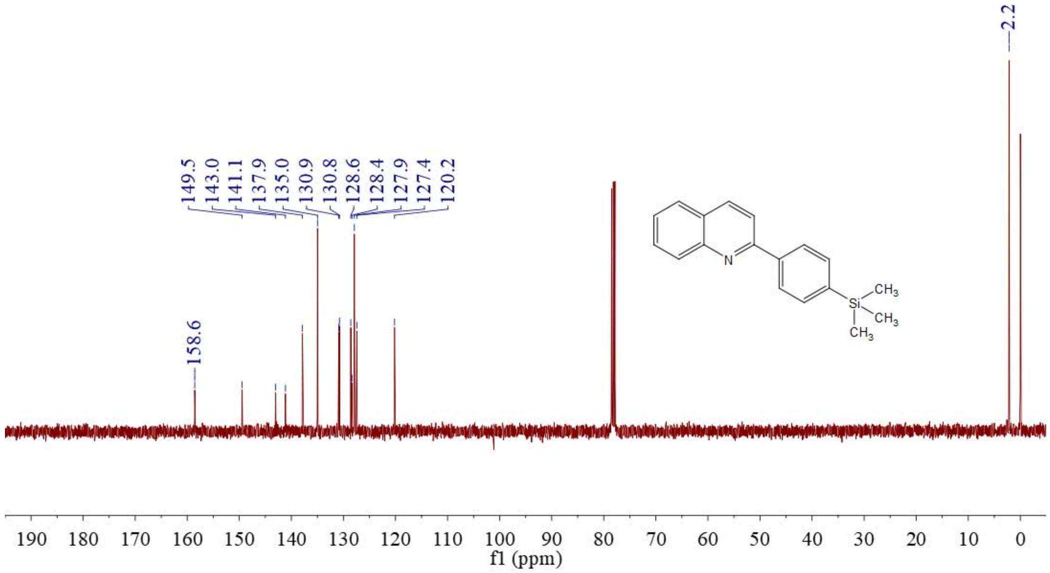

Supplementary Figure 36. ^{13}C-NMR (101 MHz, $CDCl_3$) spectrum of **C14**

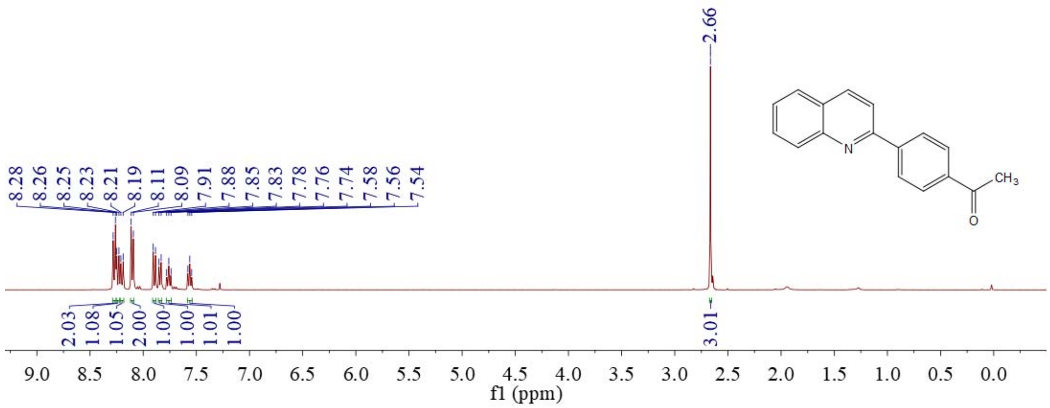

Supplementary Figure 37. ^{1}H-NMR (400 MHz, $CDCl_3$) spectrum of **C15**

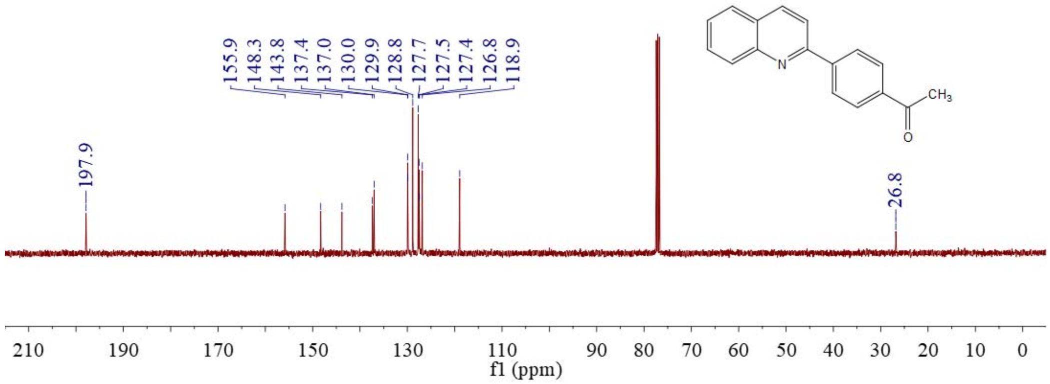

Supplementary Figure 38. ^{13}C-NMR (101 MHz, $CDCl_3$) spectrum of **C15**

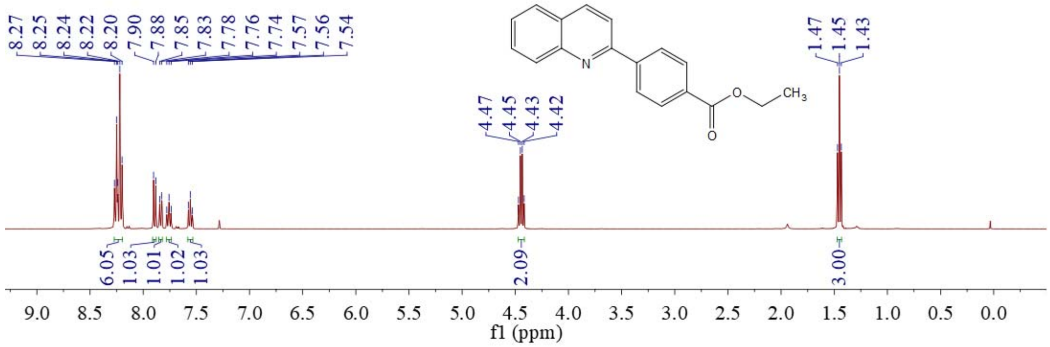

Supplementary Figure 39. ^{1}H-NMR (400 MHz, $CDCl_3$) spectrum of **C16**

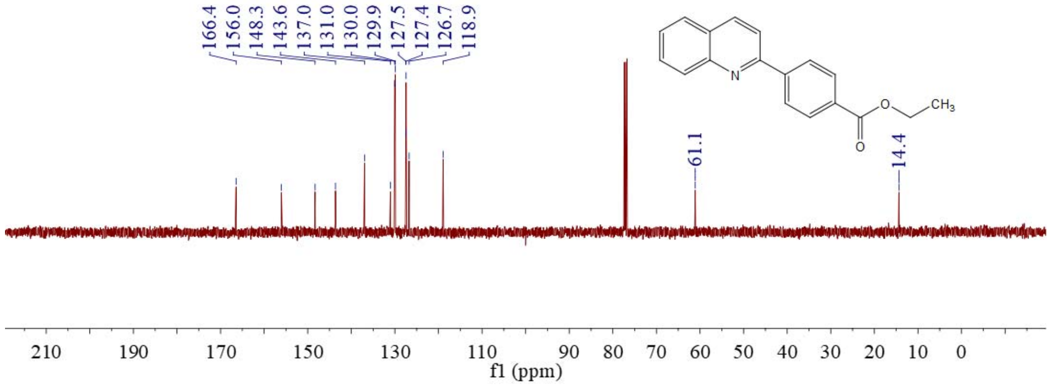

Supplementary Figure 40. ^{13}C-NMR (101 MHz, $CDCl_3$) spectrum of **C16**

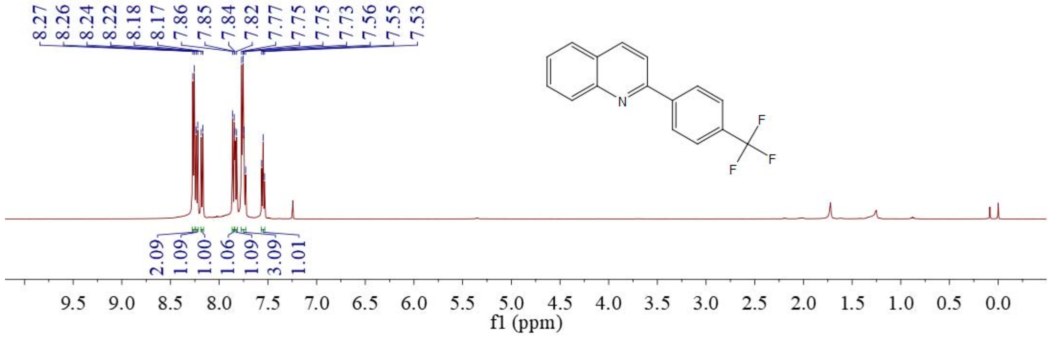

Supplementary Figure 41. ^{1}H-NMR (500 MHz, $CDCl_3$) spectrum of **C17**

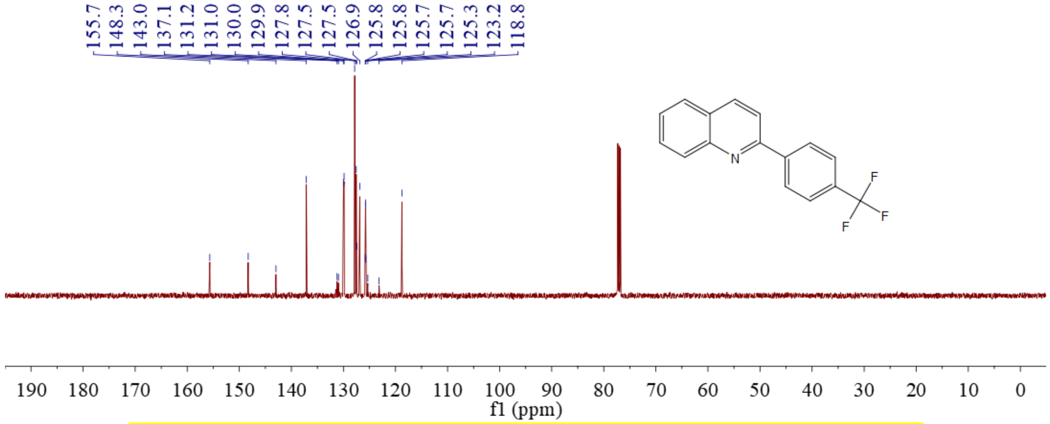

Supplementary Figure 42. ^{13}C-NMR (126 MHz, $CDCl_3$) spectrum of **C17**

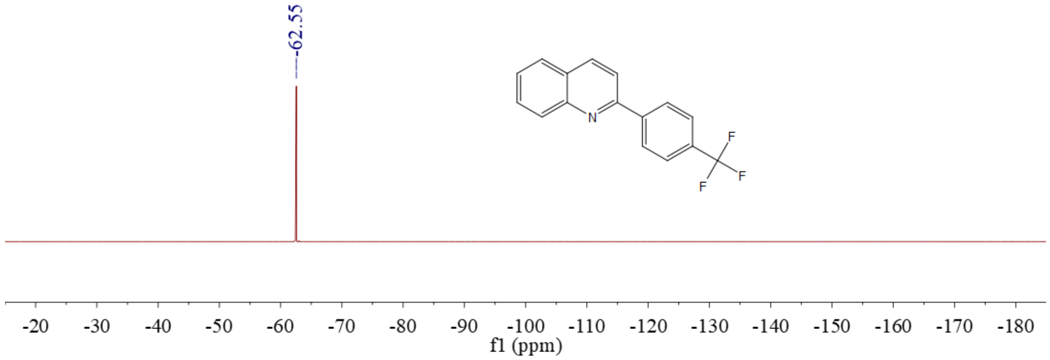

Supplementary Figure 43. ^{19}F-NMR (471 MHz, $CDCl_3$) spectrum of **C17**

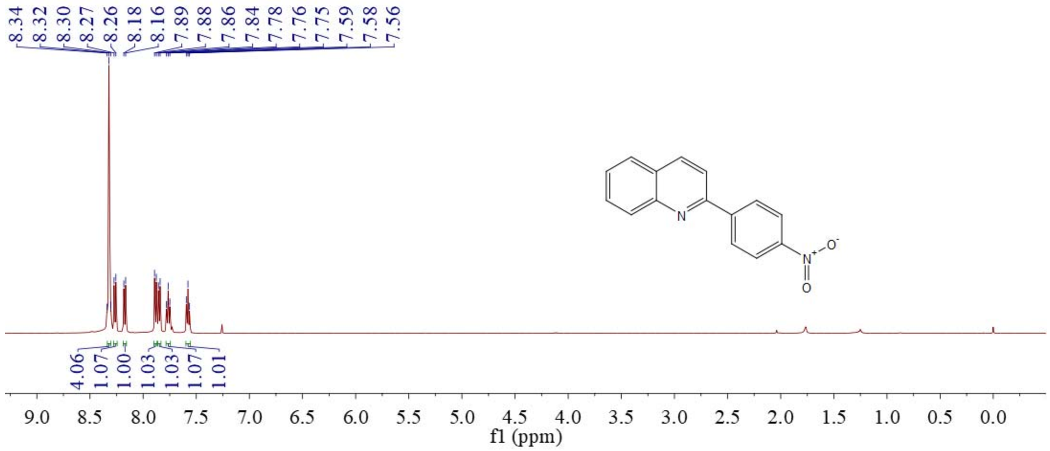

Supplementary Figure 44. ^{1}H-NMR (500 MHz, $CDCl_3$) spectrum of **C18**

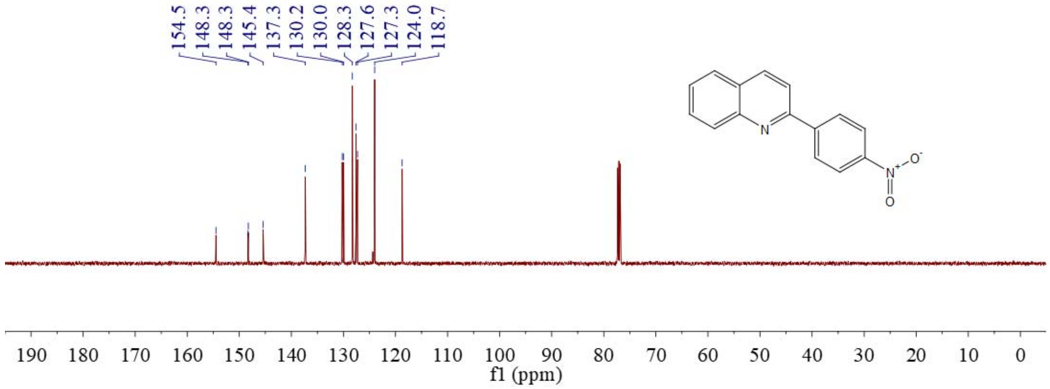

Supplementary Figure 45. ^{13}C-NMR (126 MHz, $CDCl_3$) spectrum of **C18**

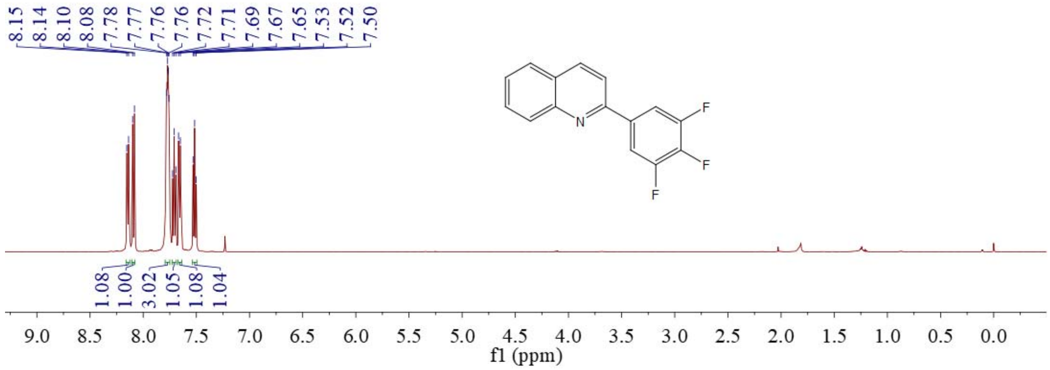

Supplementary Figure 46. ^{1}H-NMR (500 MHz, $CDCl_3$) spectrum of **C19**

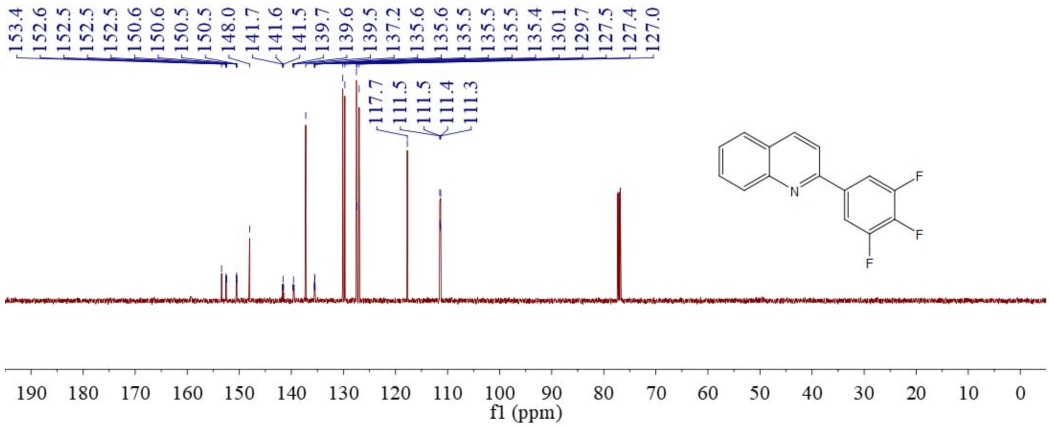

Supplementary Figure 47. ^{13}C-NMR (126 MHz, $CDCl_3$) spectrum of **C19**

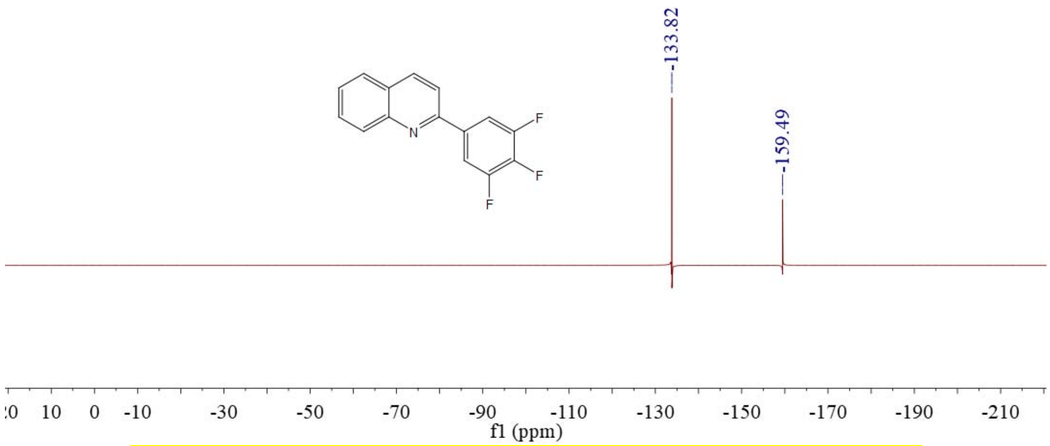

Supplementary Figure 48. ^{19}F-NMR (471 MHz, $CDCl_3$) spectrum of **C19**

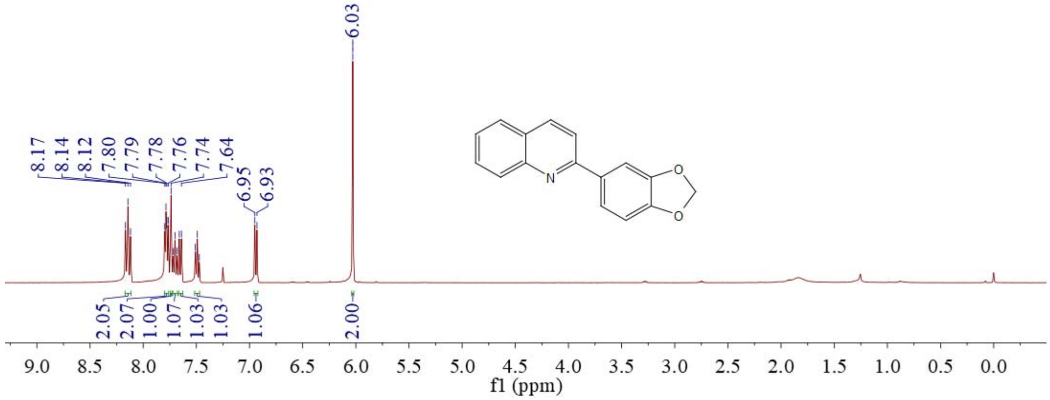

Supplementary Figure 49. ^{1}H-NMR (400 MHz, $CDCl_3$) spectrum of **C20**

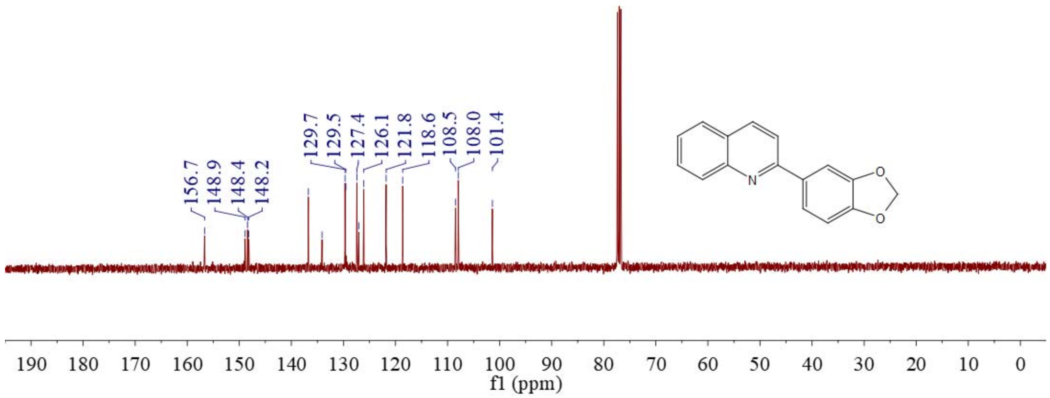

Supplementary Figure 50. ^{13}C-NMR (101 MHz, $CDCl_3$) spectrum of **C20**

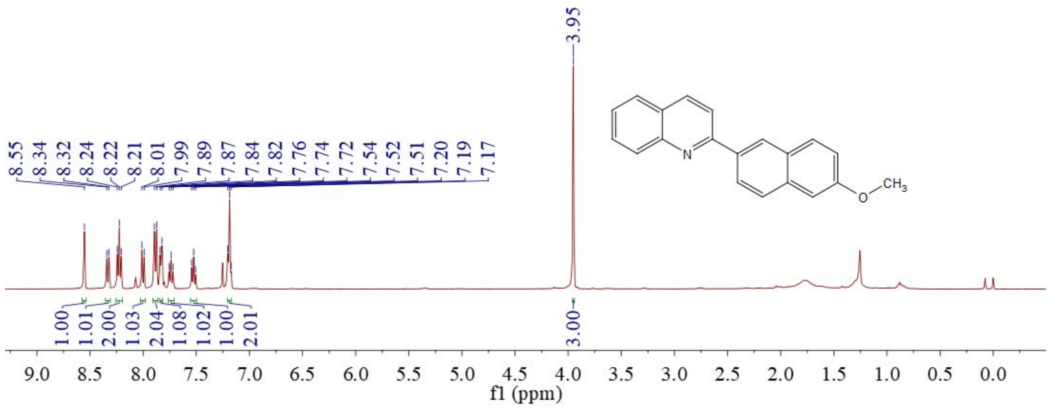

Supplementary Figure 51. ^{1}H-NMR (400 MHz, $CDCl_3$) spectrum of **C21**

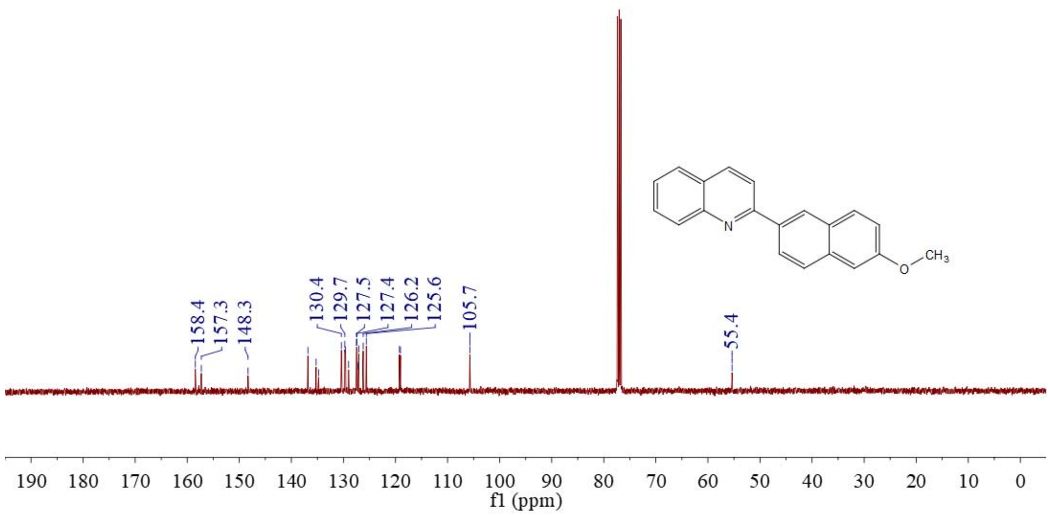

Supplementary Figure 52. ^{13}C-NMR (101 MHz, $CDCl_3$) spectrum of **C21**

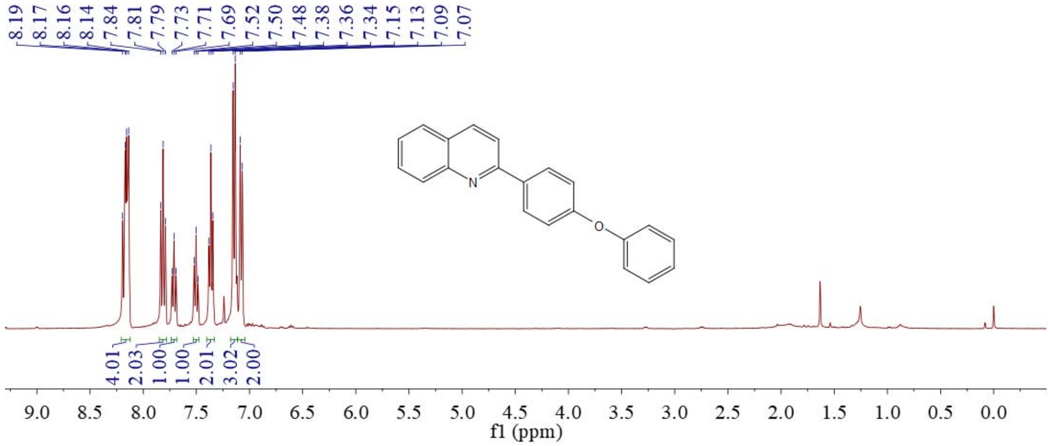

Supplementary Figure 53. ^{1}H-NMR (400 MHz, $CDCl_3$) spectrum of **C22**

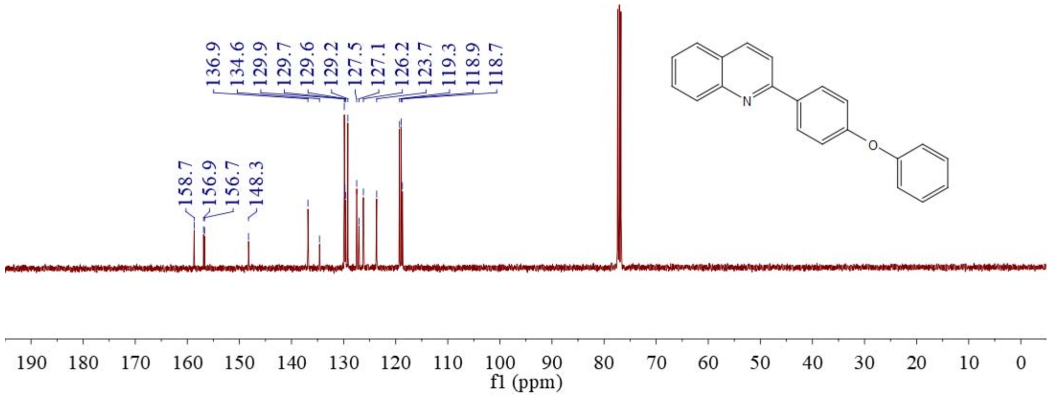

Supplementary Figure 54. ^{13}C-NMR (101 MHz, $CDCl_3$) spectrum of **C22**

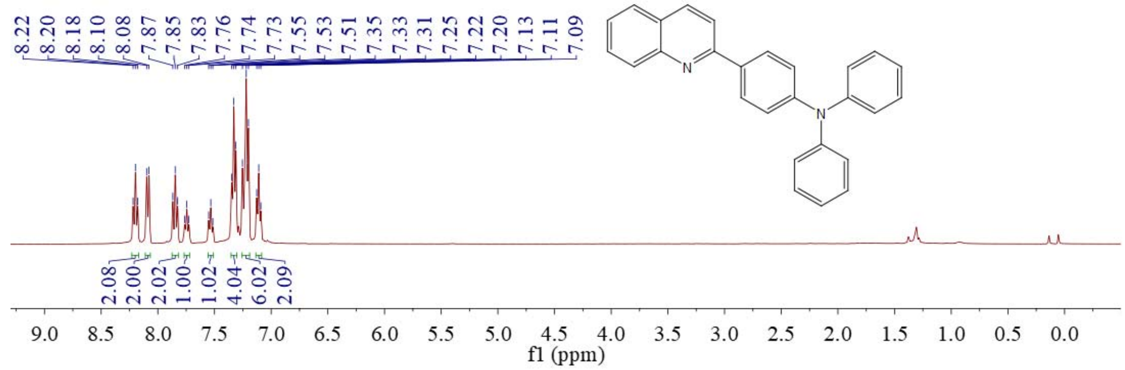

Supplementary Figure 55. ^{1}H-NMR (400 MHz, $CDCl_3$) spectrum of **C23**

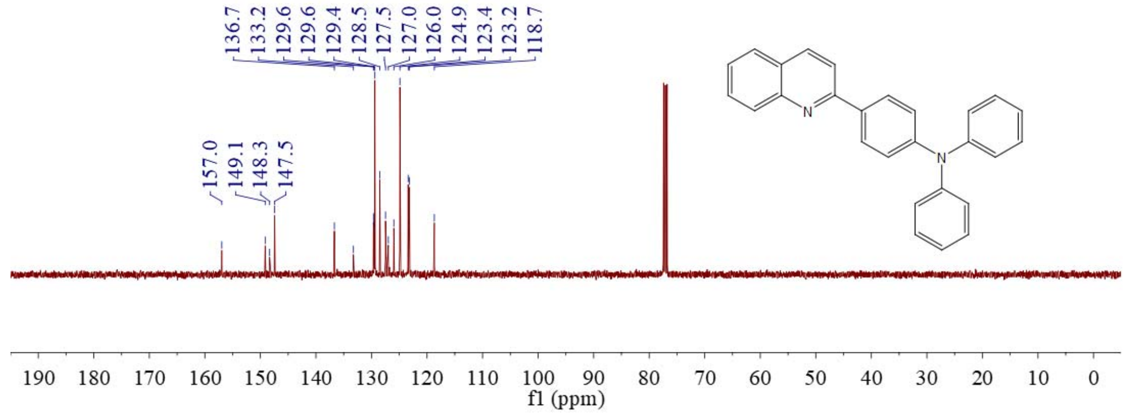

Supplementary Figure 56. ^{13}C-NMR (101 MHz, $CDCl_3$) spectrum of **C23**

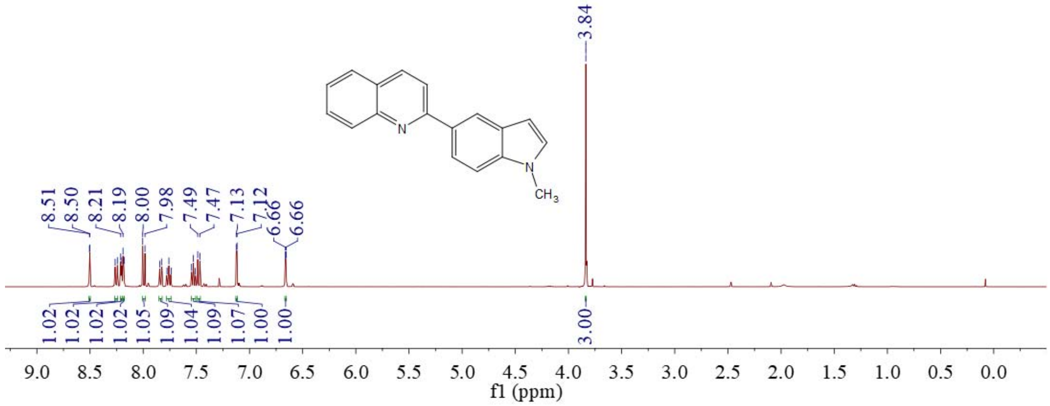

Supplementary Figure 57. ^{1}H-NMR (400 MHz, $CDCl_3$) spectrum of **C24**

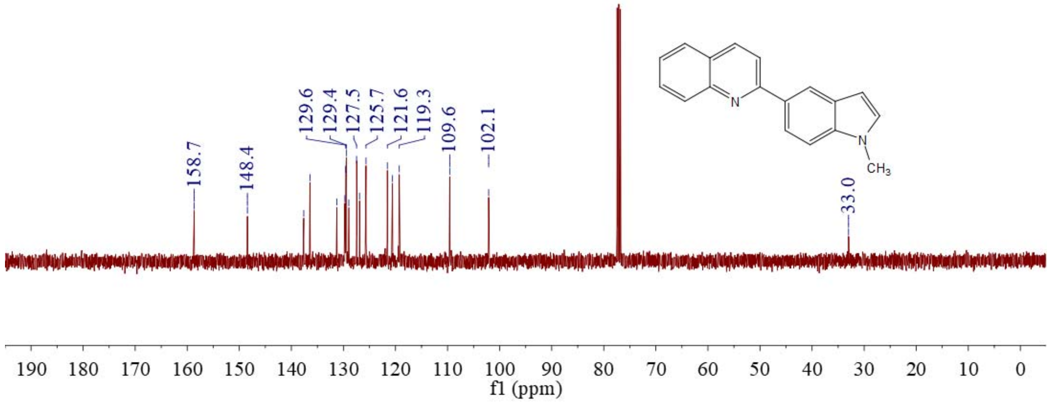

Supplementary Figure 58. ^{13}C-NMR (101 MHz, $CDCl_3$) spectrum of **C24**

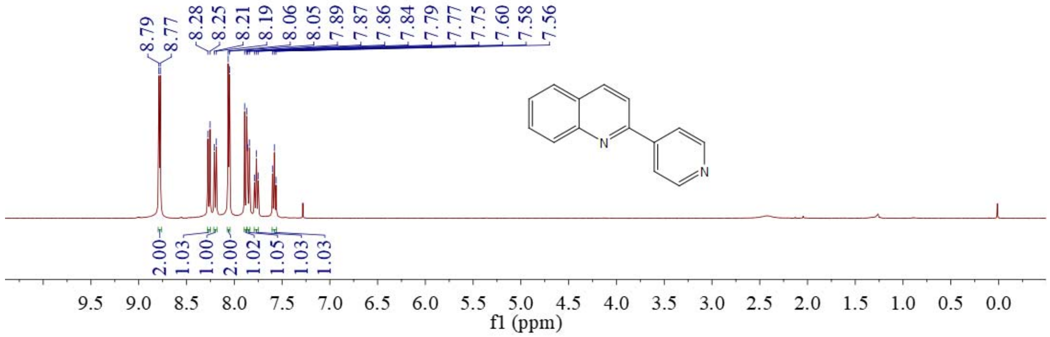

Supplementary Figure 59. ^{1}H-NMR (400 MHz, $CDCl_3$) spectrum of **C25**

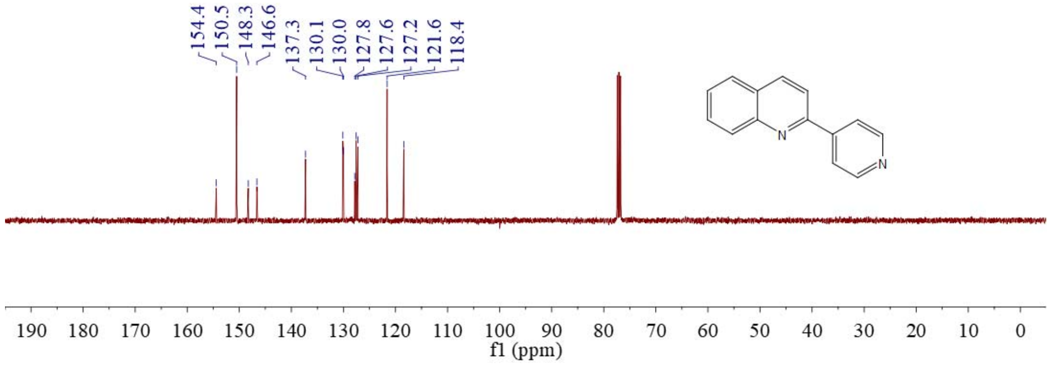

Supplementary Figure 60. ^{13}C-NMR (101 MHz, $CDCl_3$) spectrum of **C25**

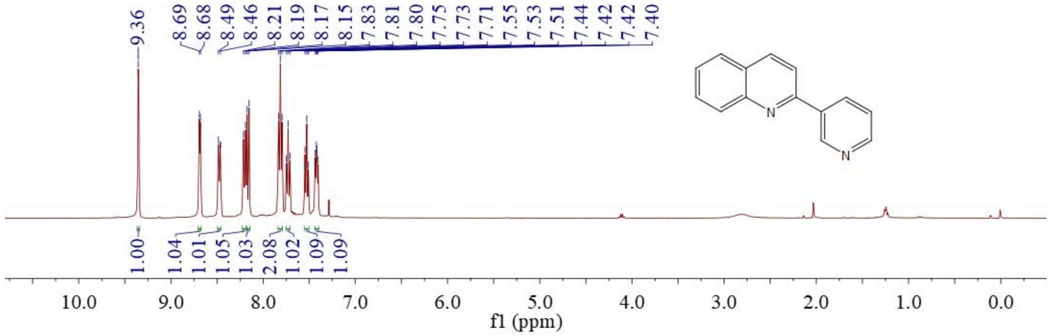

Supplementary Figure 61. ^{1}H-NMR (400 MHz, $CDCl_3$) spectrum of **C26**

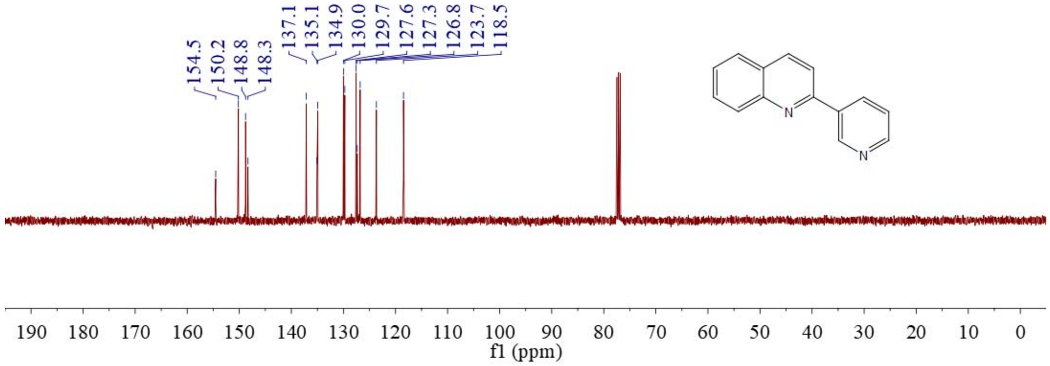

Supplementary Figure 62. ^{13}C-NMR (101 MHz, $CDCl_3$) spectrum of **26**

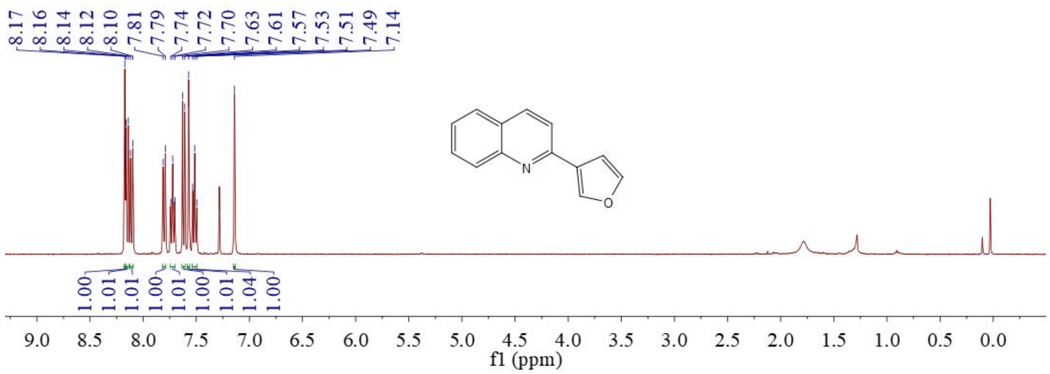

Supplementary Figure 63. ^{1}H-NMR (400 MHz, $CDCl_3$) spectrum of **C27**

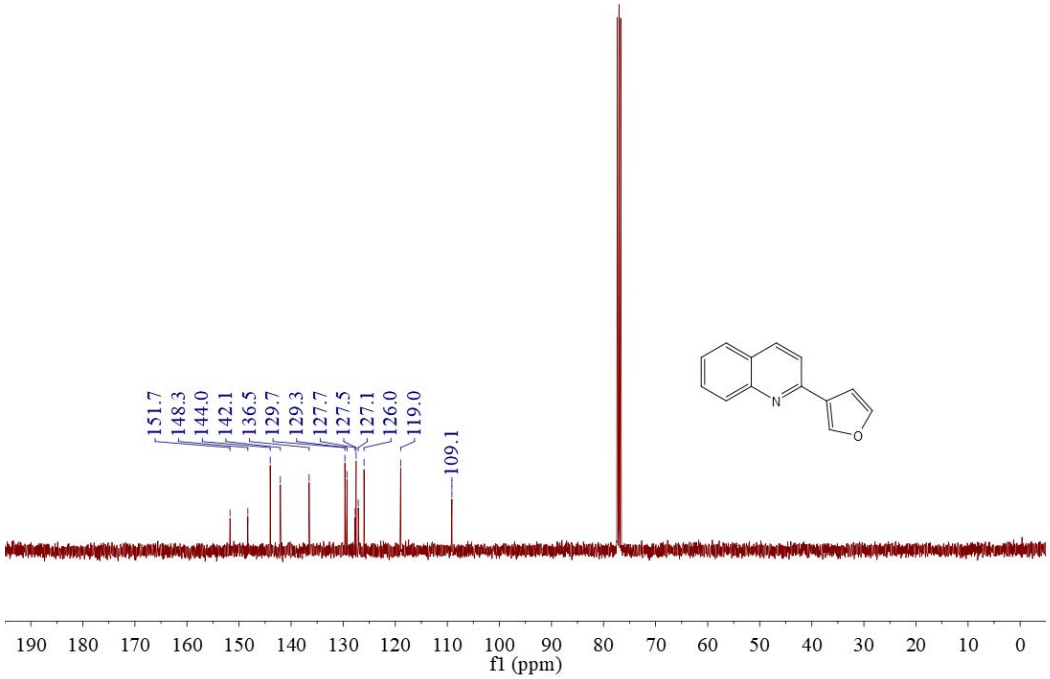

Supplementary Figure 64. ^{13}C-NMR (101 MHz, $CDCl_3$) spectrum of **C27**

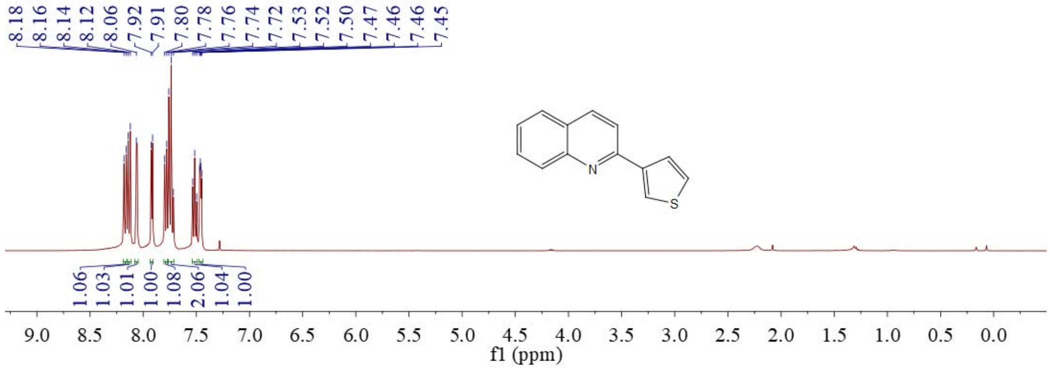

Supplementary Figure 65. ^{1}H-NMR (400 MHz, $CDCl_3$) spectrum of **C28**

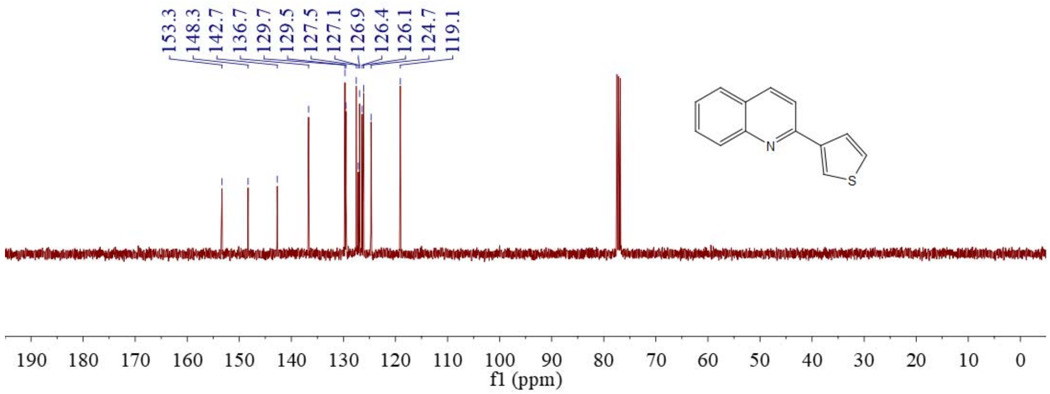

Supplementary Figure 66. ^{13}C-NMR (101 MHz, $CDCl_3$) spectrum of **C28**

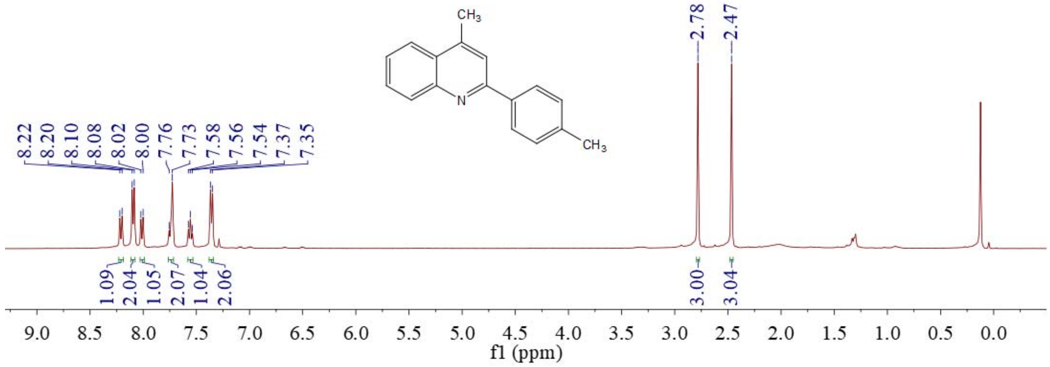

Supplementary Figure 67. ^{1}H-NMR (400 MHz, $CDCl_3$) spectrum of **C29**

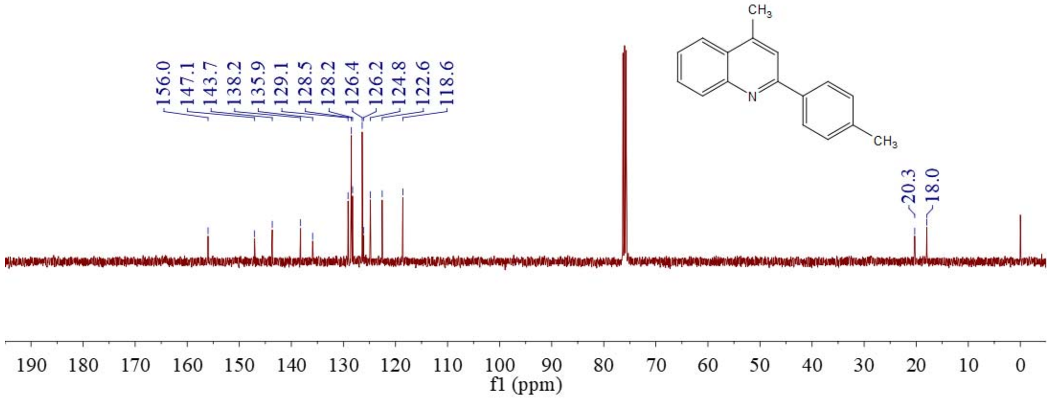

Supplementary Figure 68. ^{13}C-NMR (101 MHz, $CDCl_3$) spectrum of **C29**

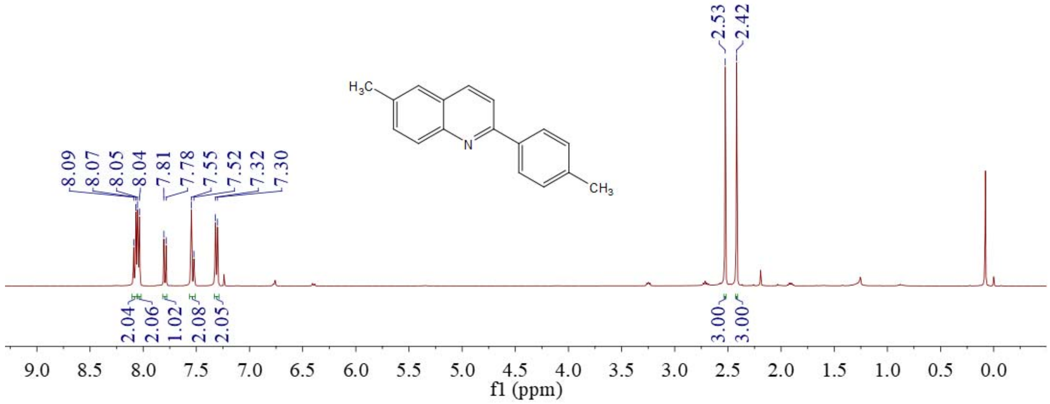

Supplementary Figure 69. ^{1}H-NMR (400 MHz, $CDCl_3$) spectrum of **C30**

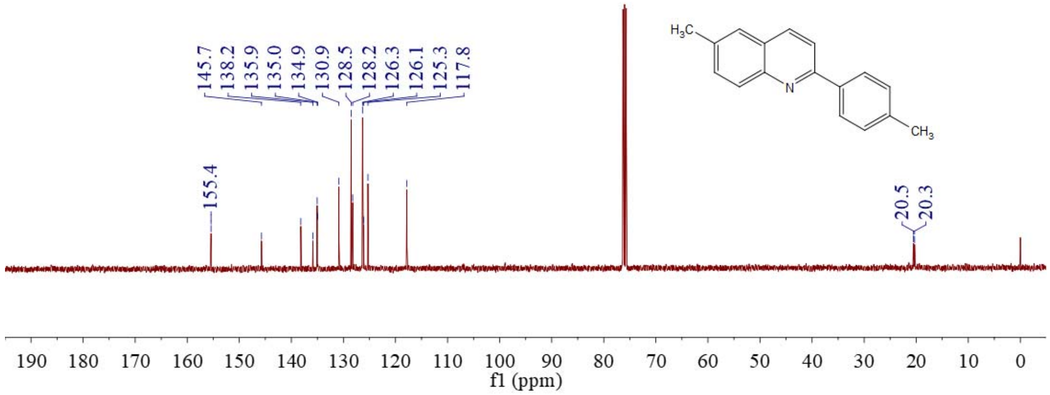

Supplementary Figure 70. ^{13}C-NMR (101 MHz, $CDCl_3$) spectrum of **C30**

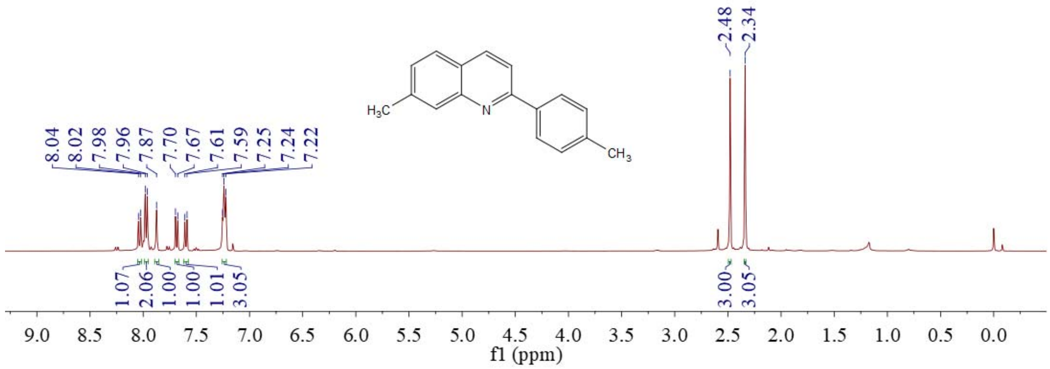

Supplementary Figure 71. ^{1}H-NMR (400 MHz, $CDCl_3$) spectrum of **C31**

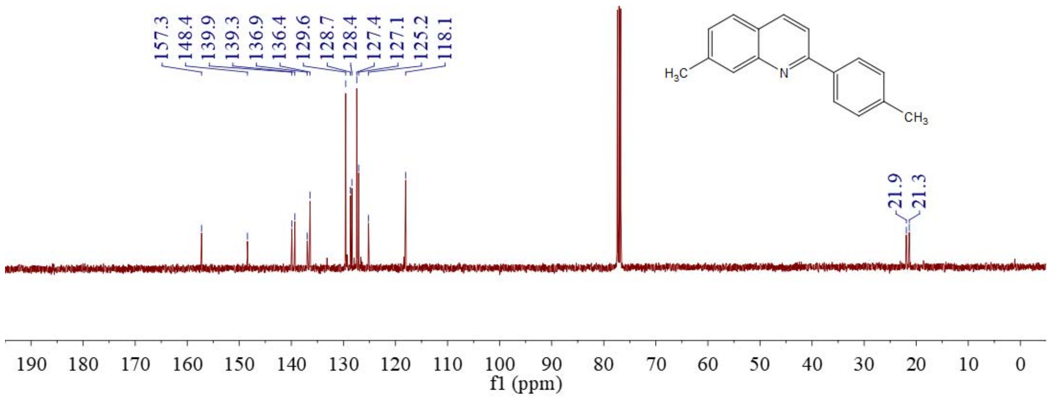

Supplementary Figure 72. ^{13}C-NMR (101 MHz, $CDCl_3$) spectrum of **C31**

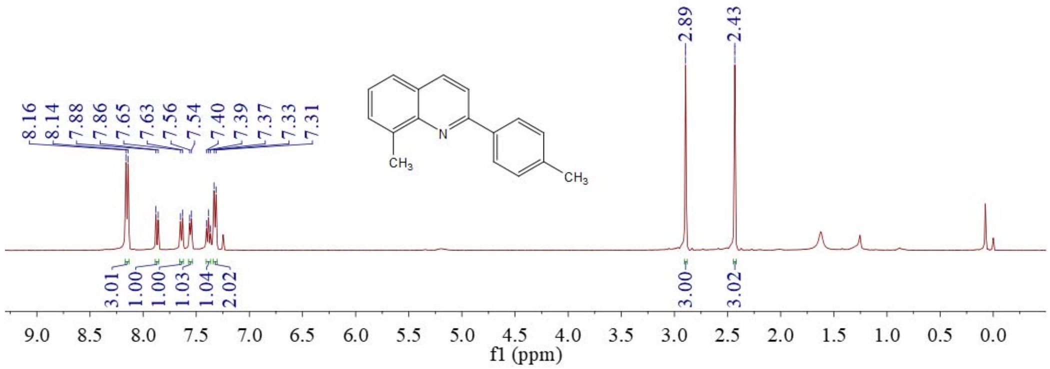

Supplementary Figure 73. ^{1}H-NMR (400 MHz, $CDCl_3$) spectrum of **C32**

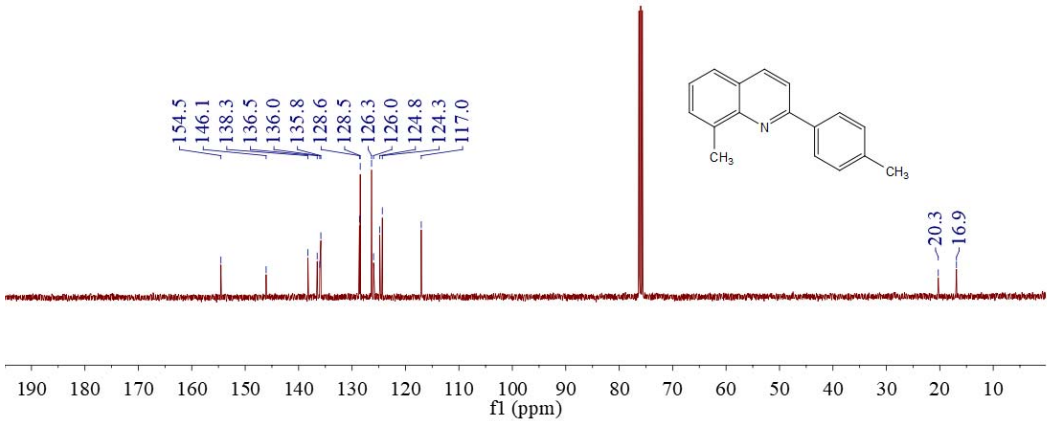

Supplementary Figure 74. ^{13}C-NMR (101 MHz, $CDCl_3$) spectrum of **C32**

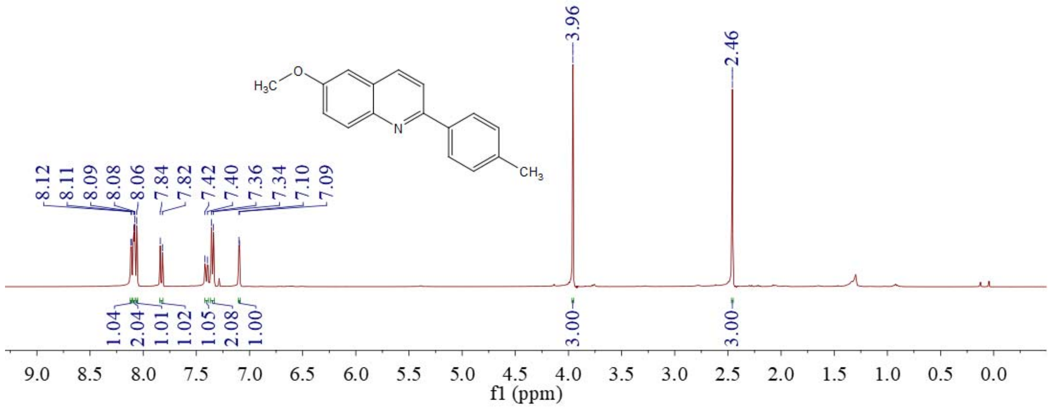

Supplementary Figure 75. ^{1}H-NMR (400 MHz, $CDCl_3$) spectrum of **C33**

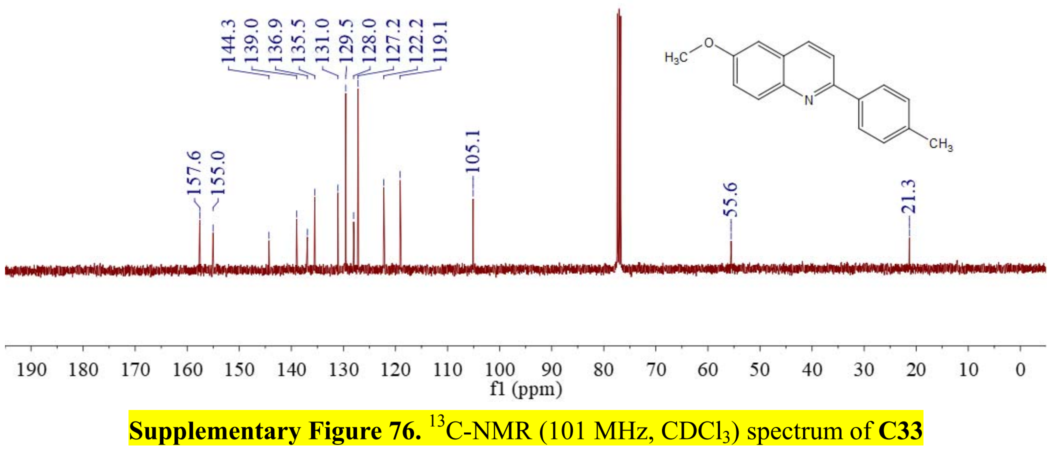

Supplementary Figure 76. ^{13}C-NMR (101 MHz, $CDCl_3$) spectrum of **C33**

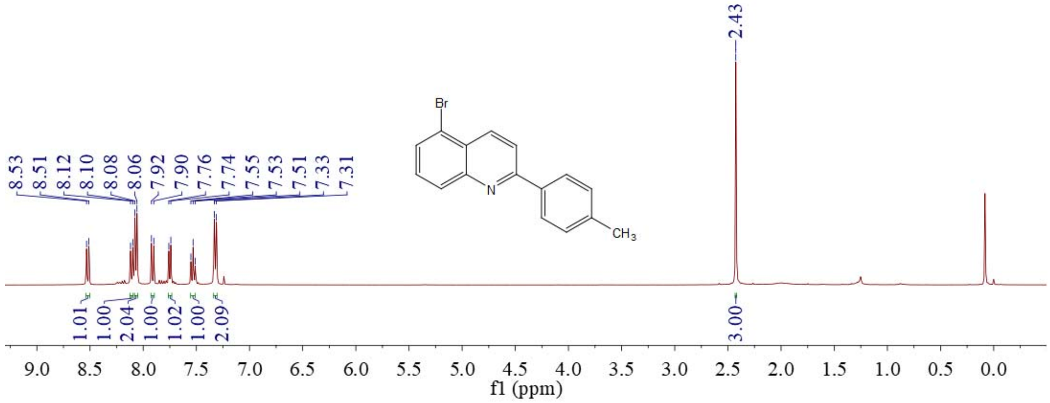

Supplementary Figure 77. ^{1}H-NMR (400 MHz, $CDCl_3$) spectrum of **C34**

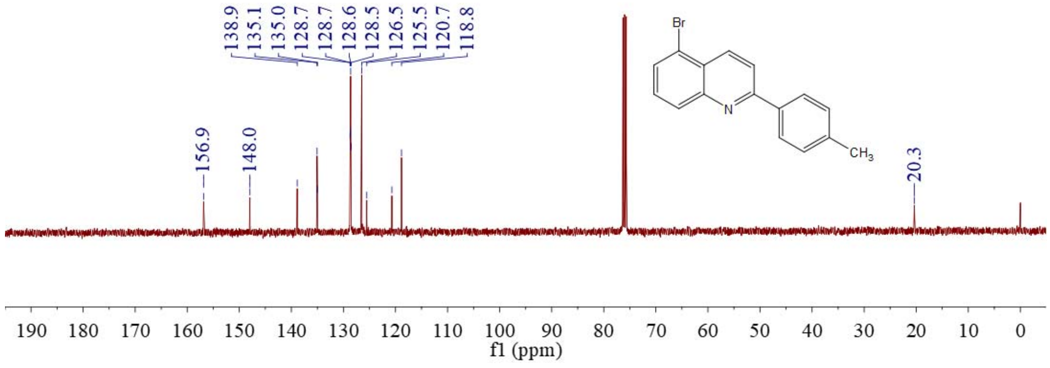

Supplementary Figure 78. ^{13}C-NMR (101 MHz, $CDCl_3$) spectrum of **C34**

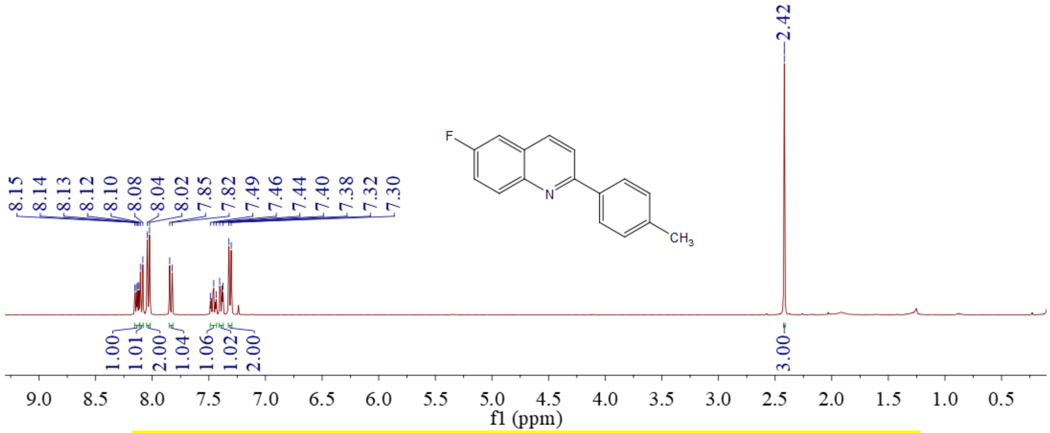

Supplementary Figure 79. ^{1}H-NMR (400 MHz, $CDCl_3$) spectrum of **C35**

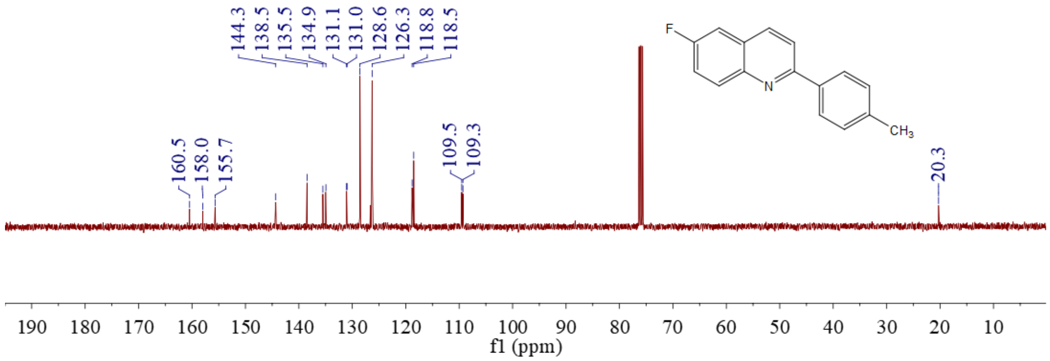

Supplementary Figure 80. ^{13}C-NMR (101 MHz, $CDCl_3$) spectrum of **C35**

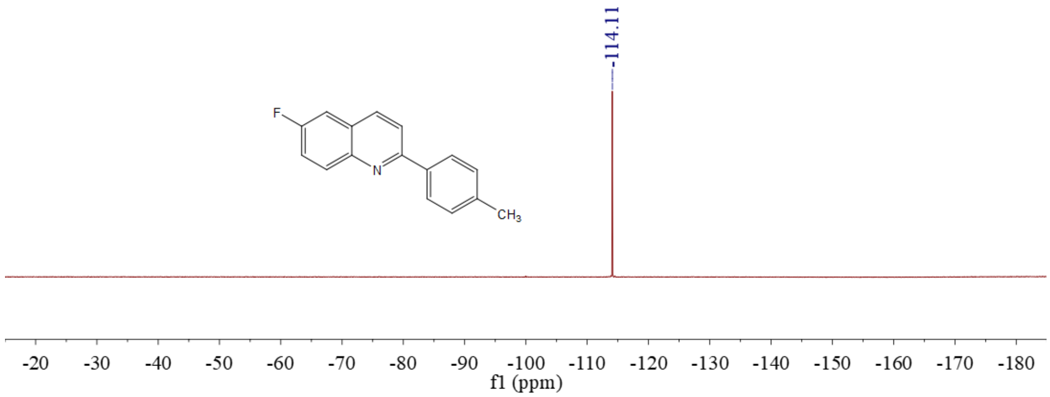

Supplementary Figure 81. ^{19}F-NMR (376 MHz, $CDCl_3$) spectrum of **C35**

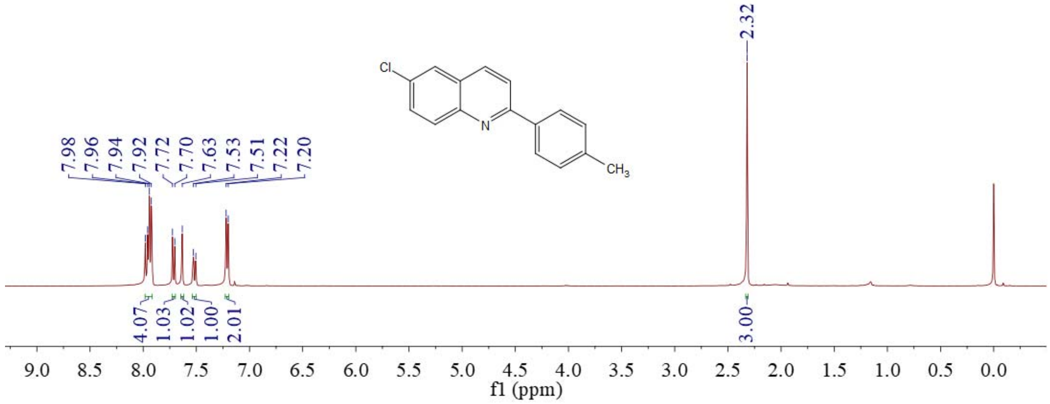

Supplementary Figure 82. ^{1}H-NMR (400 MHz, $CDCl_3$) spectrum of **C36**

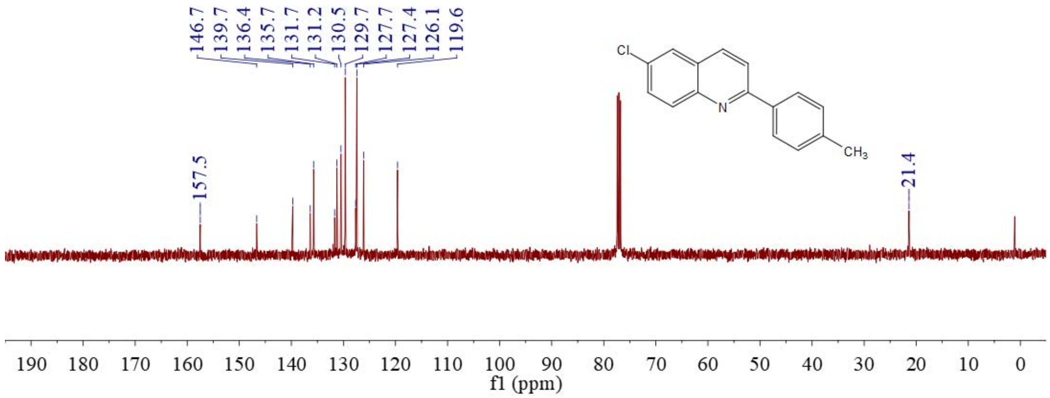

Supplementary Figure 83. ^{13}C-NMR (101 MHz, $CDCl_3$) spectrum of **C36**

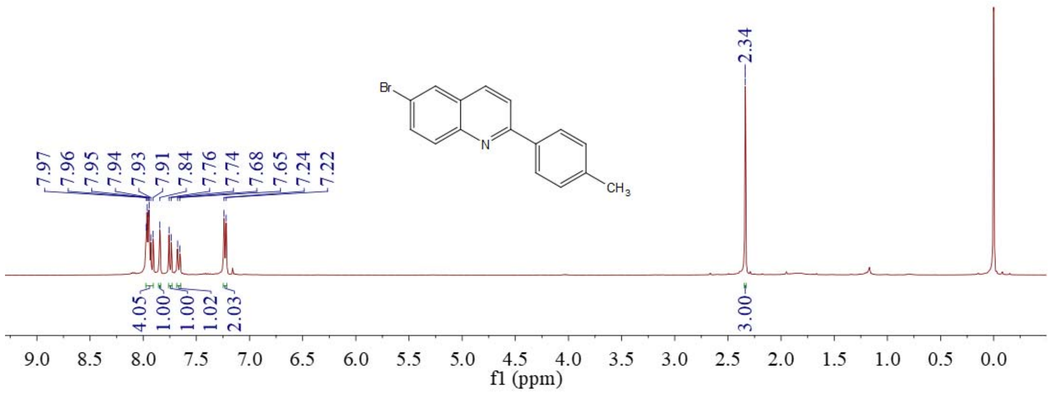

Supplementary Figure 84. ^{1}H-NMR (400 MHz, $CDCl_3$) spectrum of **C37**

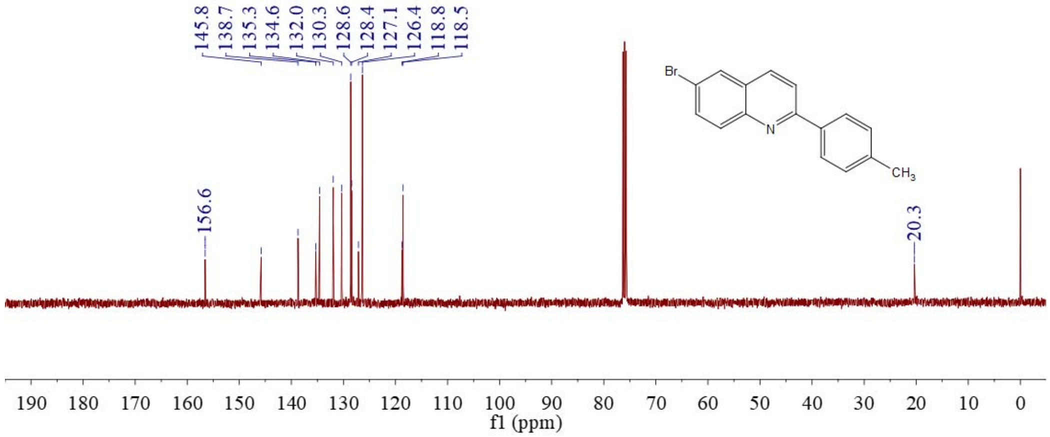

Supplementary Figure 85. ^{13}C-NMR (101 MHz, $CDCl_3$) spectrum of **C37**

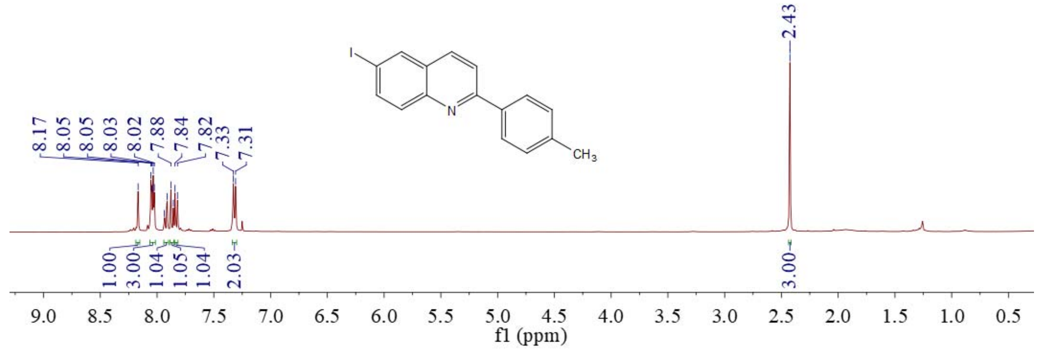

Supplementary Figure 86. ^{1}H-NMR (400 MHz, $CDCl_3$) spectrum of **C38**

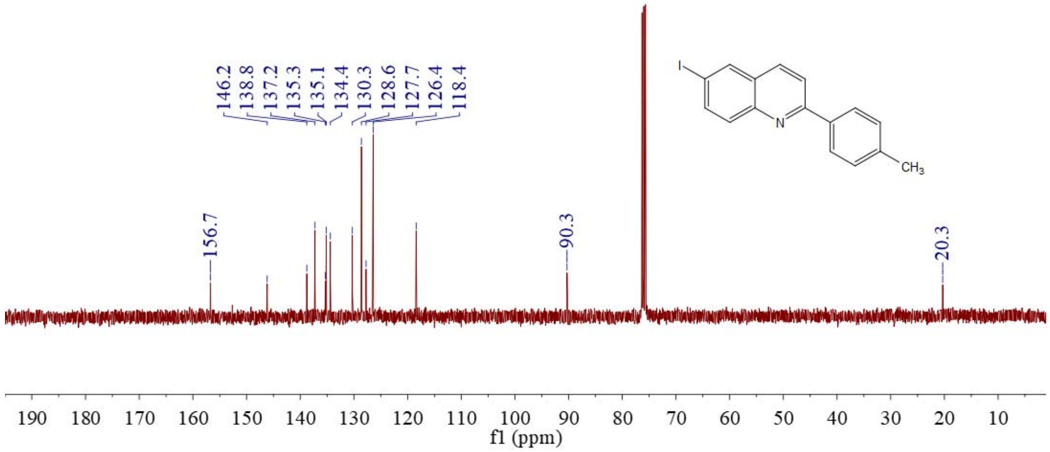

Supplementary Figure 87. ^{13}C-NMR (101 MHz, $CDCl_3$) spectrum of **C38**

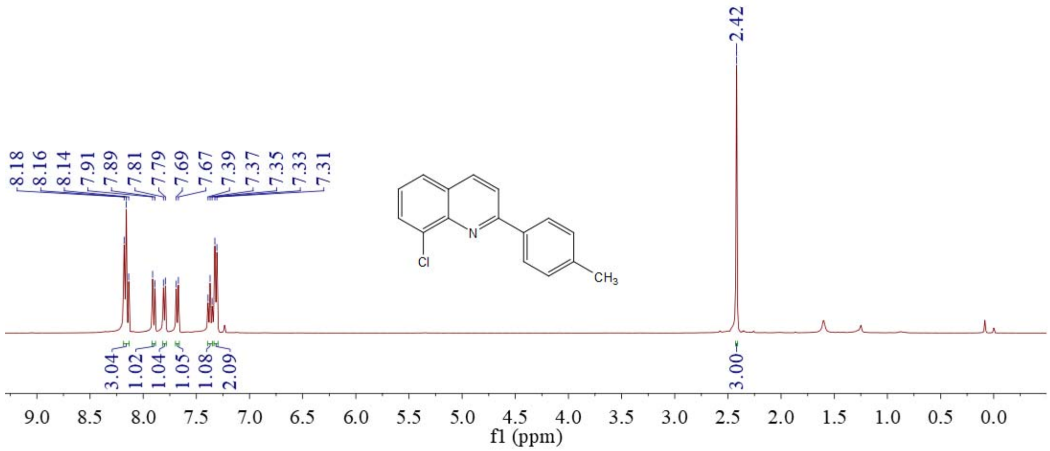

Supplementary Figure 88. ^{1}H-NMR (400 MHz, $CDCl_3$) spectrum of **C39**

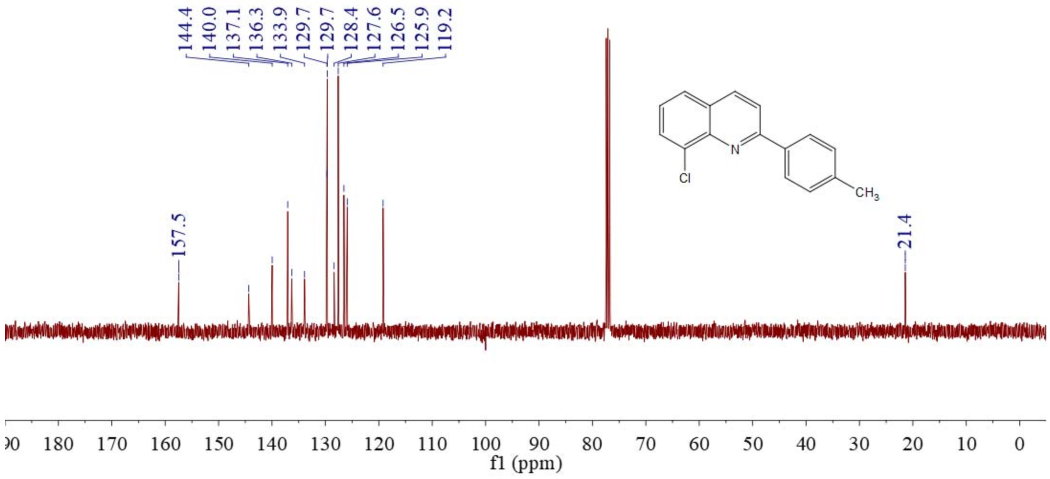

Supplementary Figure 89. ^{13}C-NMR (101 MHz, $CDCl_3$) spectrum of **C39**

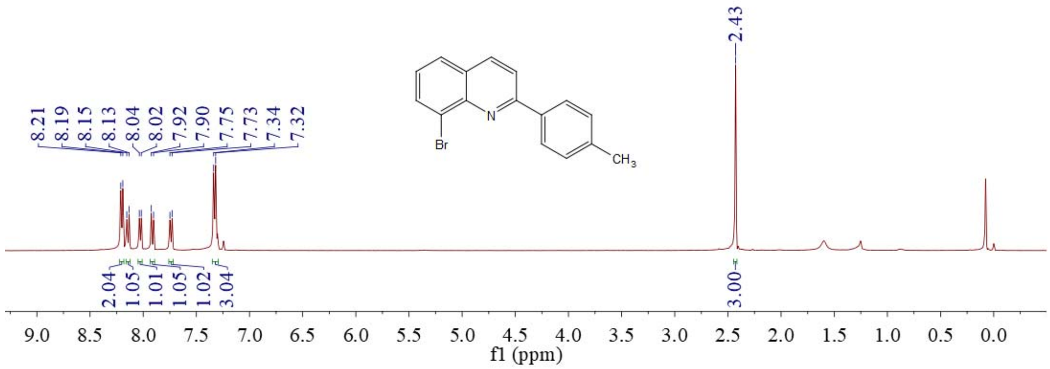

Supplementary Figure 90. ^{1}H-NMR (400 MHz, $CDCl_3$) spectrum of **C40**

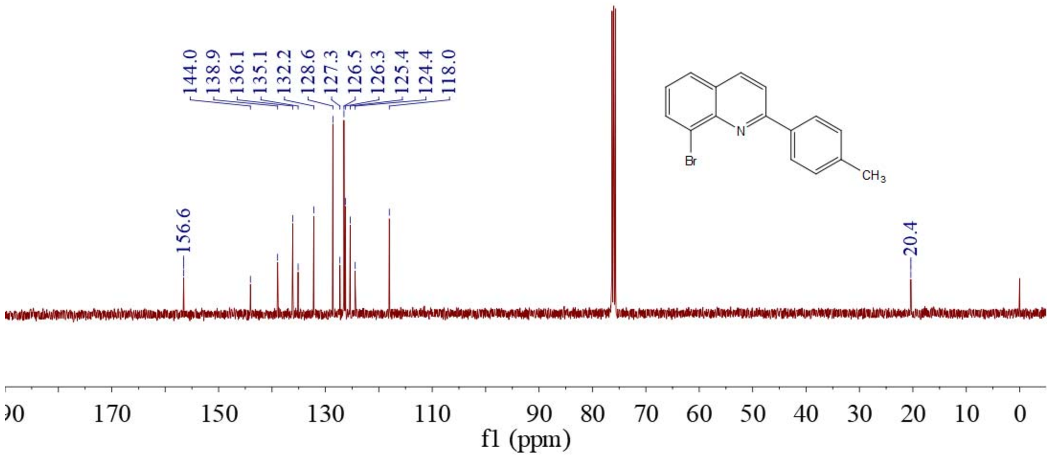

Supplementary Figure 91. ^{13}C-NMR (101 MHz, $CDCl_3$) spectrum of **C40**

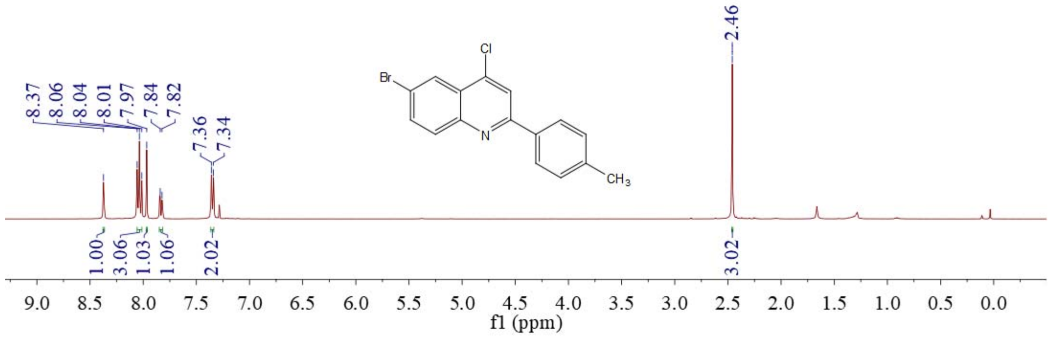

Supplementary Figure 92. ^{1}H-NMR (400 MHz, $CDCl_3$) spectrum of **C41**

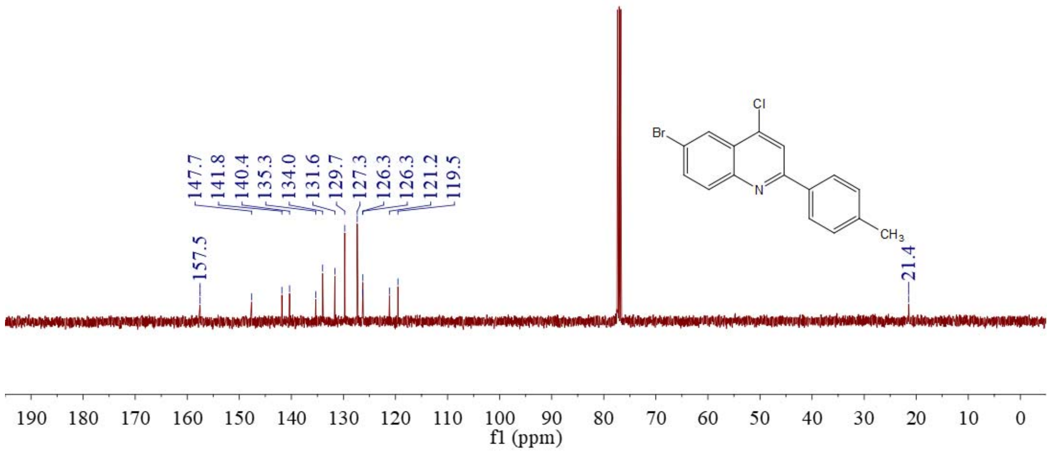

Supplementary Figure93. ^{13}C-NMR (101 MHz, $CDCl_3$) spectrum of **C41**

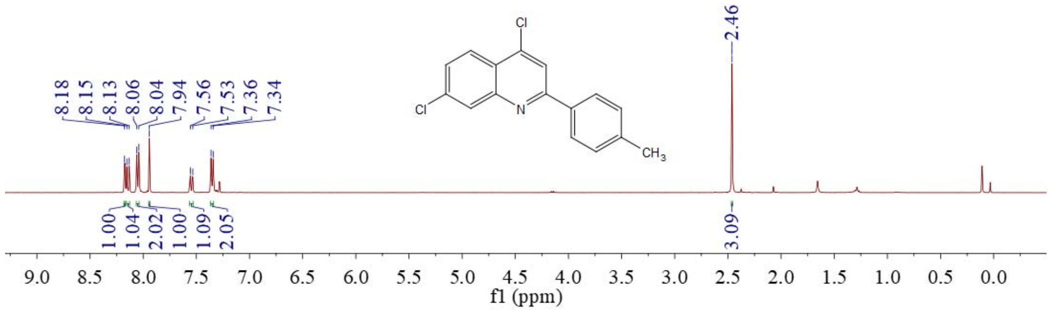

Supplementary Figure 94. ^{1}H-NMR (400 MHz, $CDCl_3$) spectrum of **C42**

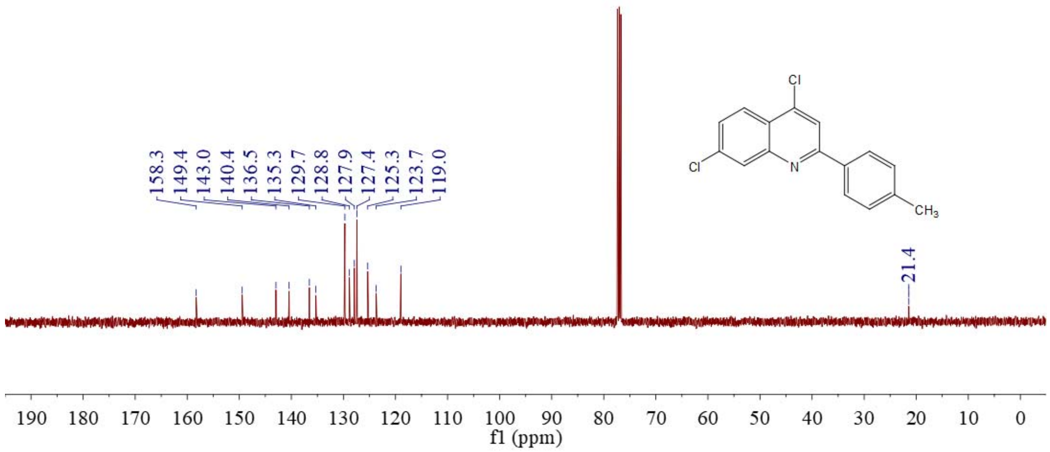

Supplementary Figure 95. ^{1}H-NMR (400 MHz, $CDCl_3$) spectrum of **C42**

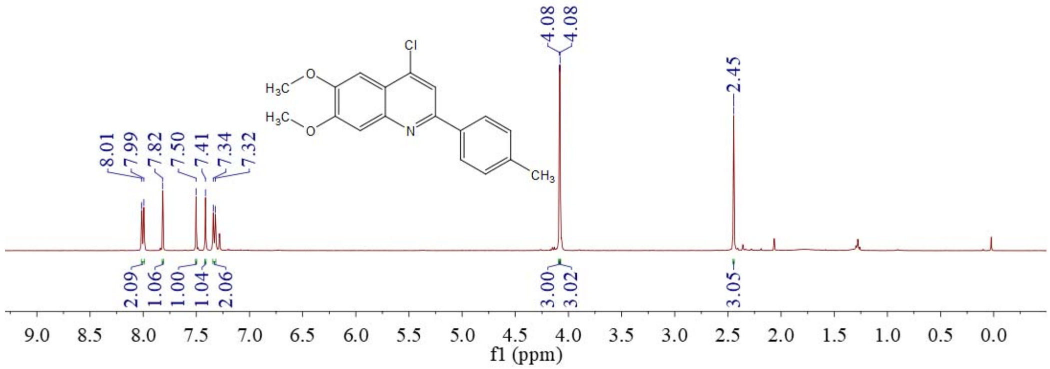

Supplementary Figure 96. ^{1}H-NMR (400 MHz, $CDCl_3$) spectrum of **C43**

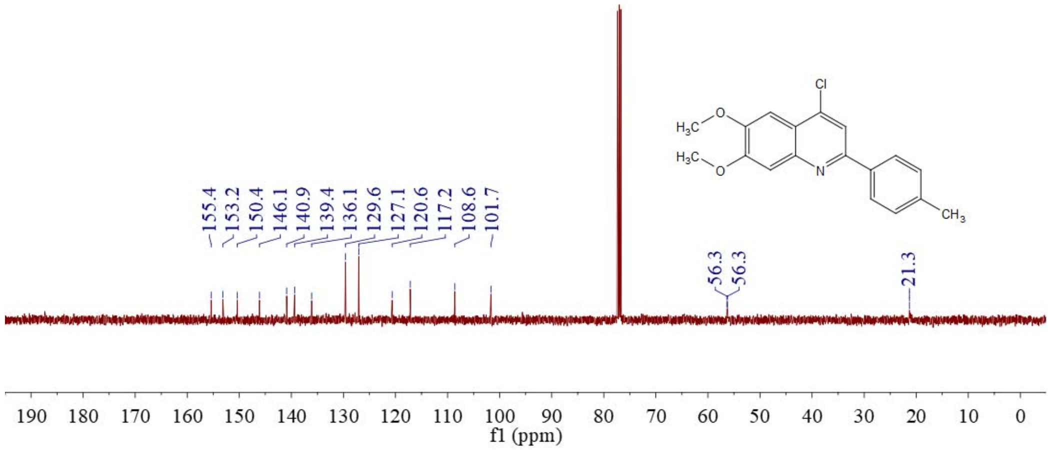

Supplementary Figure 97. ^{1}H-NMR (400 MHz, $CDCl_3$) spectrum of **C43**

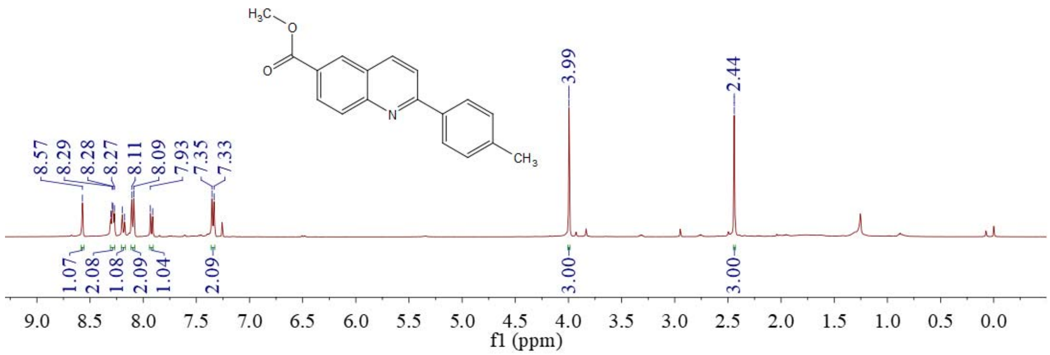

Supplementary Figure 98. ^{1}H-NMR (400 MHz, $CDCl_3$) spectrum of **C44**

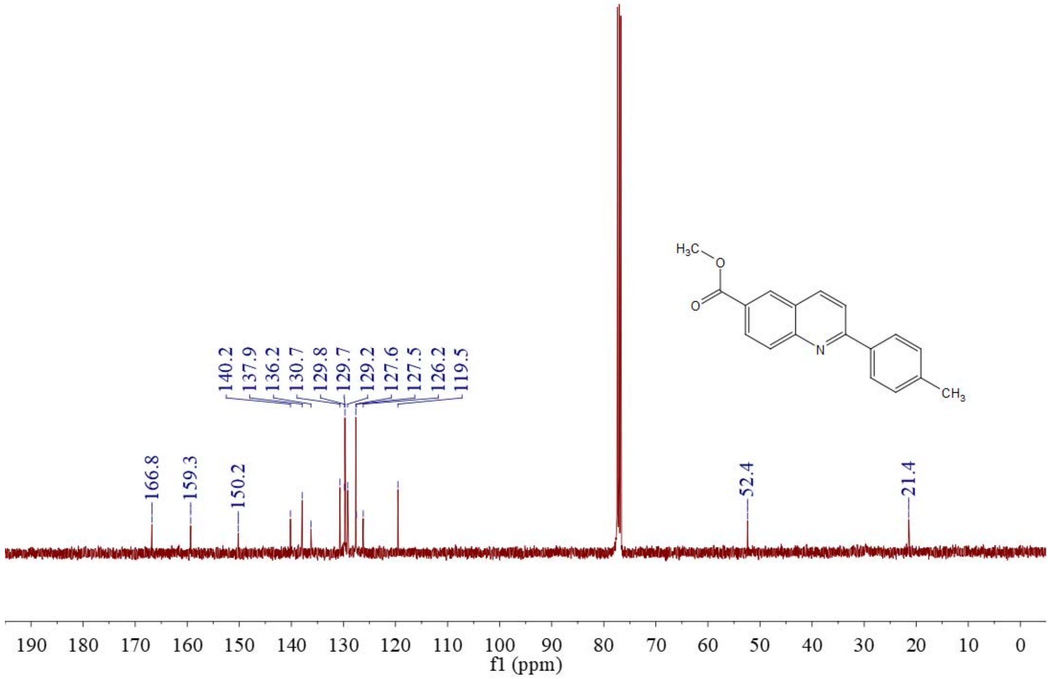

Supplementary Figure 99. ^{13}C-NMR (101 MHz, $CDCl_3$) spectrum of **C44**

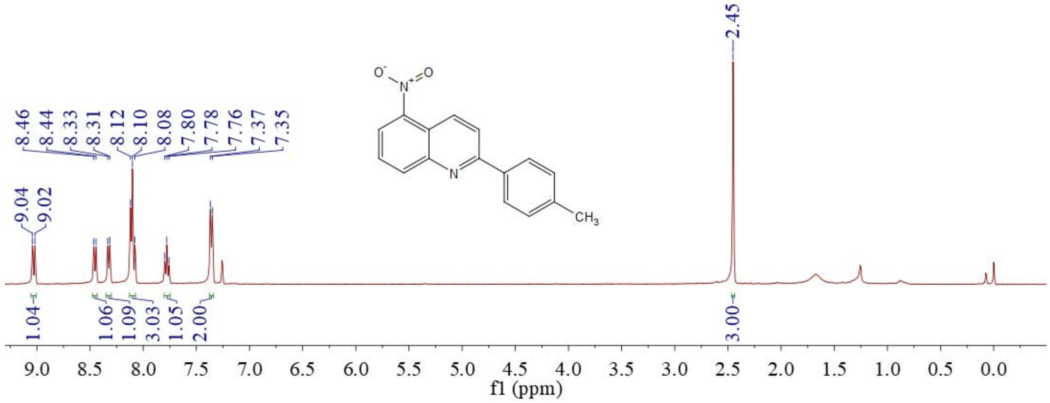

Supplementary Figure 100. ^{1}H-NMR (400 MHz, $CDCl_3$) spectrum of **C45**

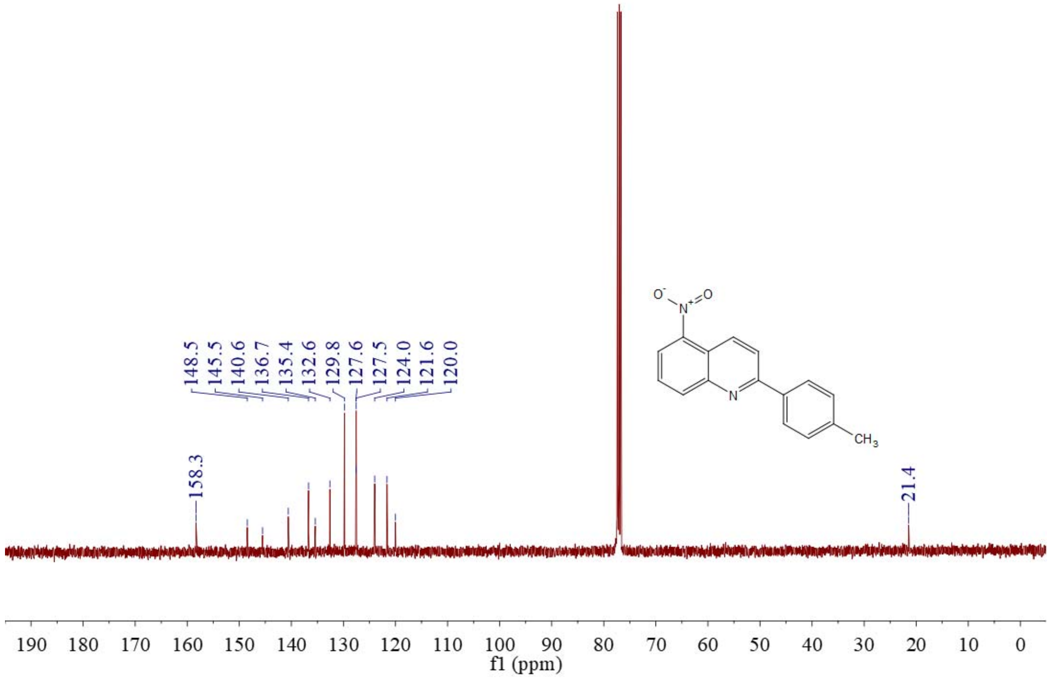

Supplementary Figure 101. ^{13}C-NMR (101 MHz, $CDCl_3$) spectrum of **C45**

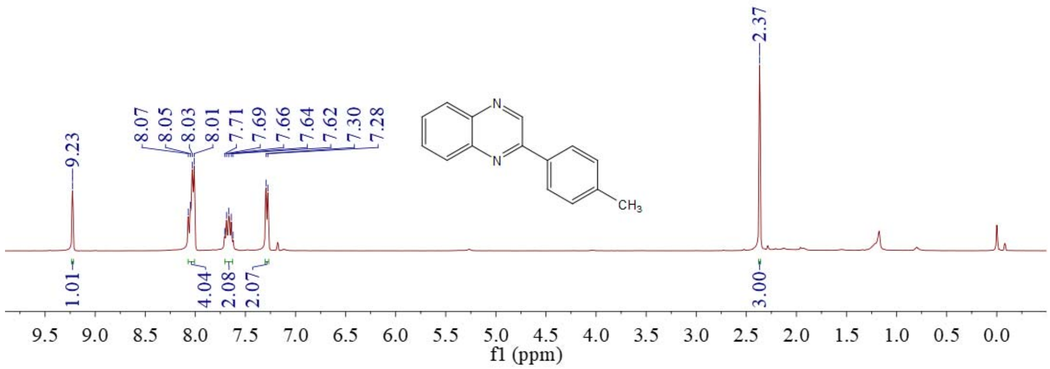

Supplementary Figure 102. ^{1}H-NMR (400 MHz, $CDCl_3$) spectrum of **C46**

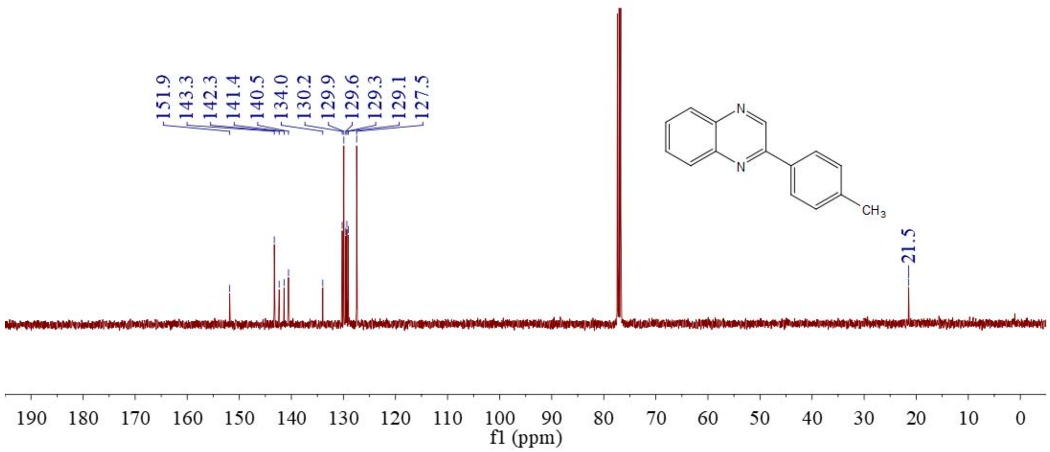

Supplementary Figure 103. ^{13}C-NMR (101 MHz, $CDCl_3$) spectrum of **C46**

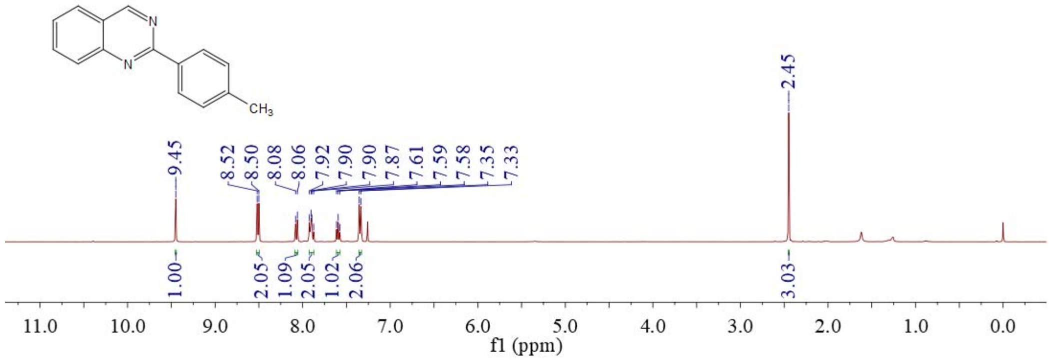

Supplementary Figure 104. ^{1}H-NMR (400 MHz, $CDCl_3$) spectrum of **C47**

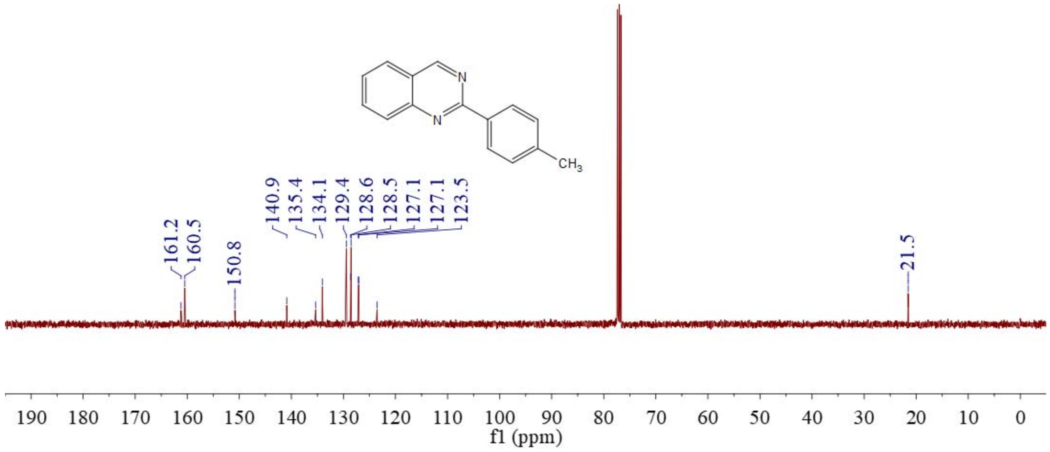

Supplementary Figure 105. ^{13}C-NMR (101 MHz, $CDCl_3$) spectrum of **C47**

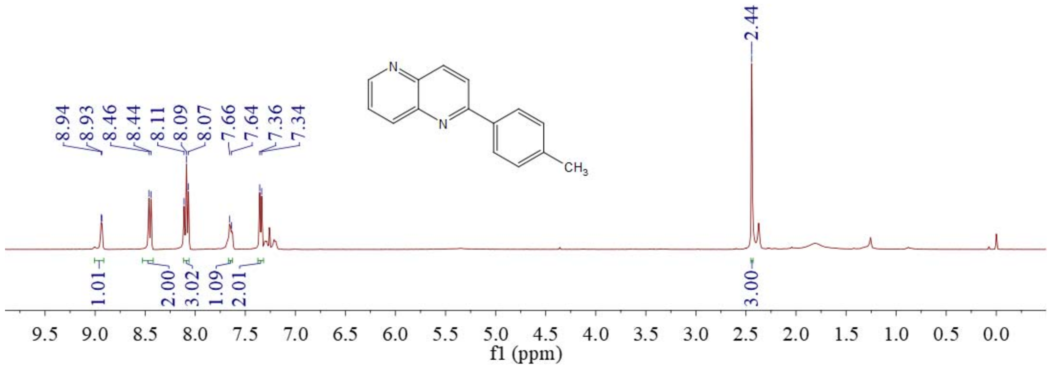

Supplementary Figure 106. ^{1}H-NMR (400 MHz, $CDCl_3$) spectrum of **C48**

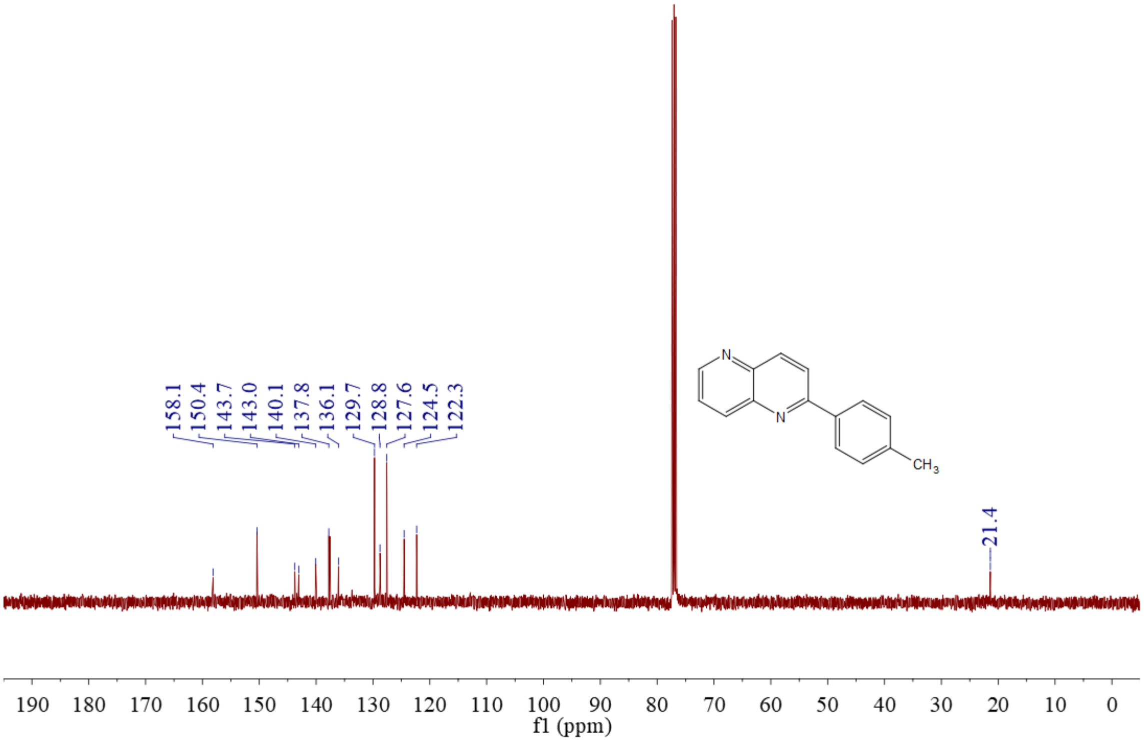

Supplementary Figure 107. ^{13}C-NMR (101 MHz, $CDCl_3$) spectrum of **C48**

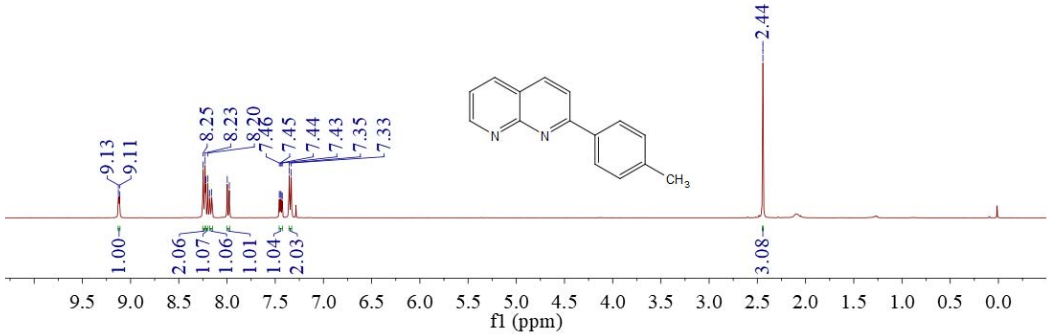

Supplementary Figure 108. ^{1}H-NMR (400 MHz, $CDCl_3$) spectrum of **C49**

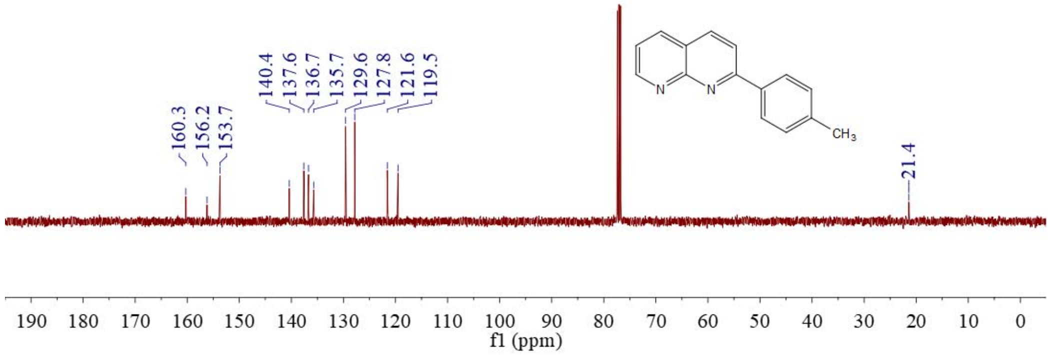

Supplementary Figure 109. ^{13}C-NMR (101 MHz, $CDCl_3$) spectrum of **C49**

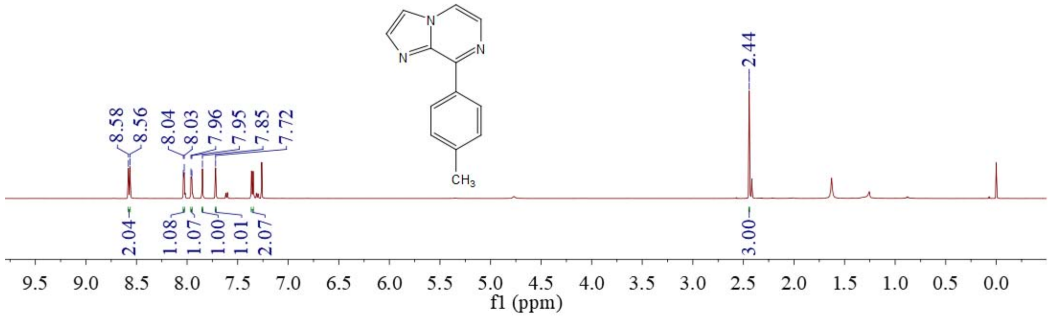

Supplementary Figure 110. ^{1}H-NMR (500 MHz, $CDCl_3$) spectrum of **C50**

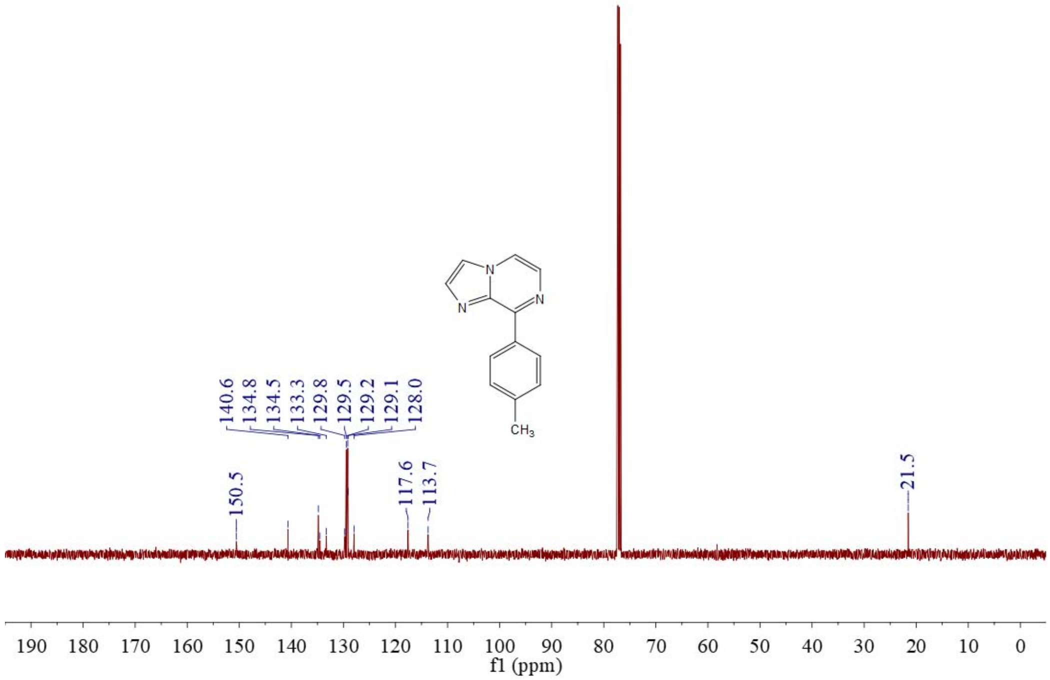

Supplementary Figure 111. ^{13}C-NMR (126 MHz, $CDCl_3$) spectrum of **C50**

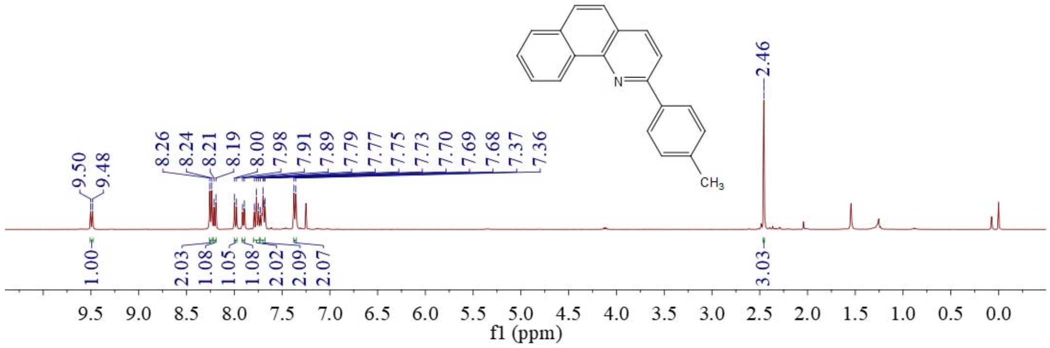

Supplementary Figure 112. ^{1}H-NMR (400 MHz, $CDCl_3$) spectrum of **C51**

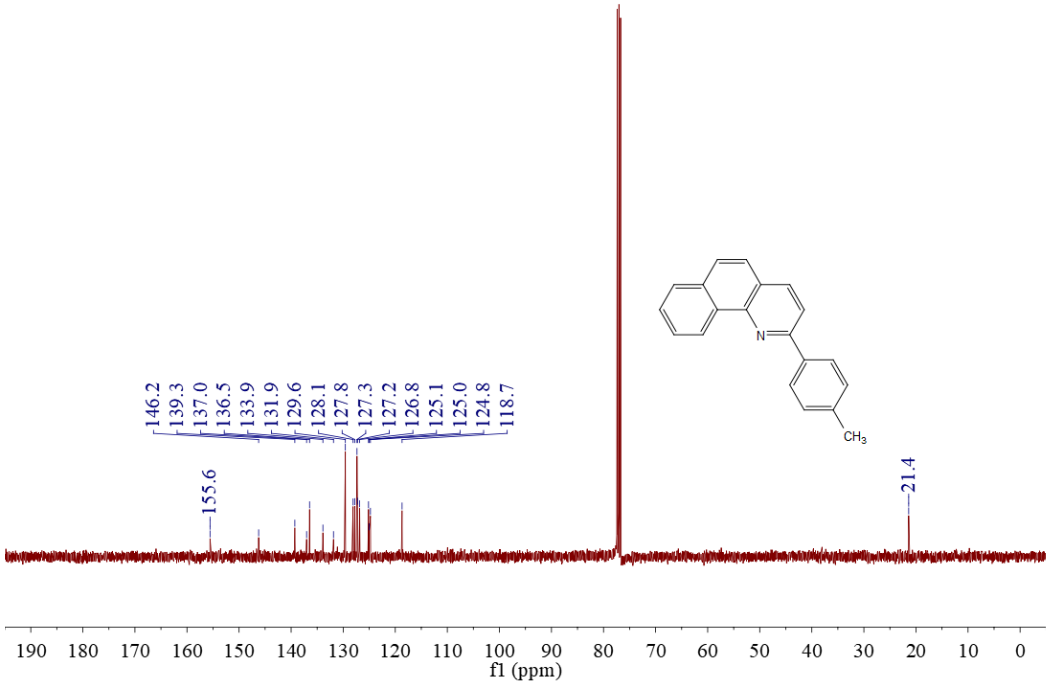

Supplementary Figure 113. ^{13}C-NMR (101 MHz, $CDCl_3$) spectrum of **C51**

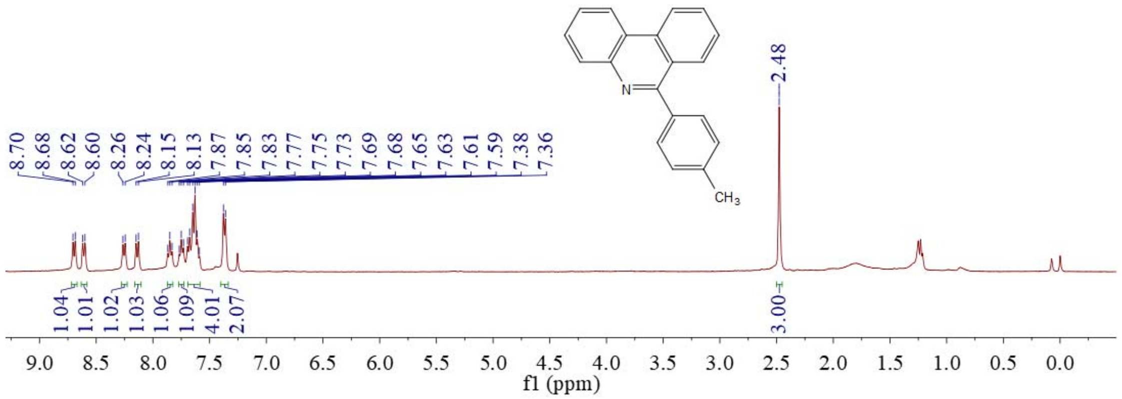

Supplementary Figure 114. ^{1}H-NMR (400 MHz, $CDCl_3$) spectrum of **C52**

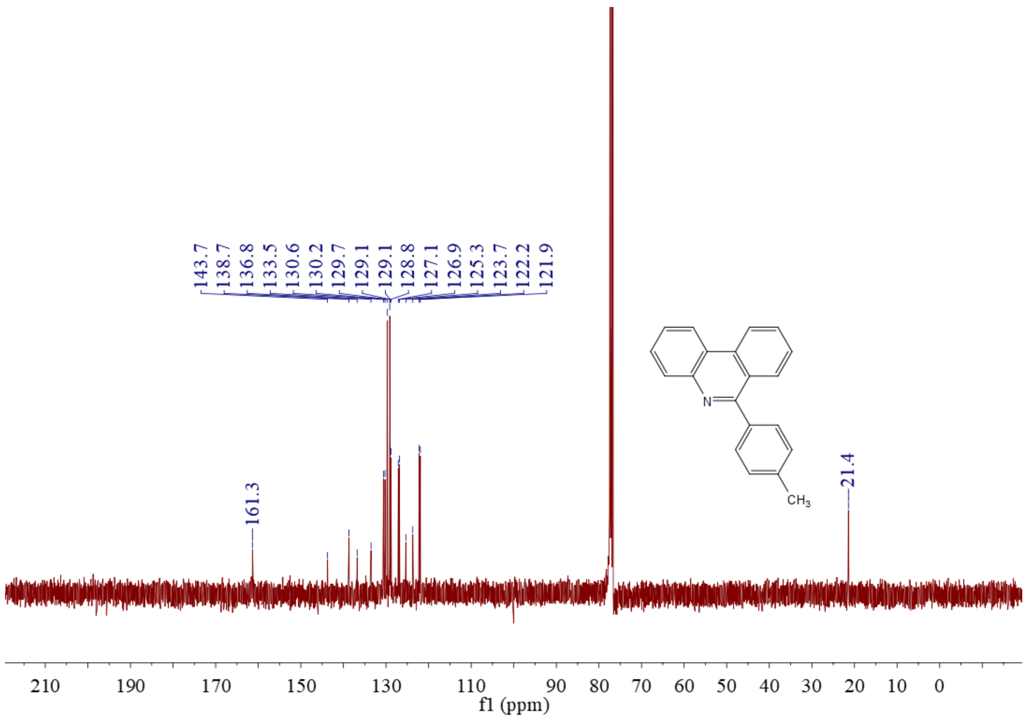

Supplementary Figure 115. ^{13}C-NMR (101 MHz, $CDCl_3$) spectrum of C52

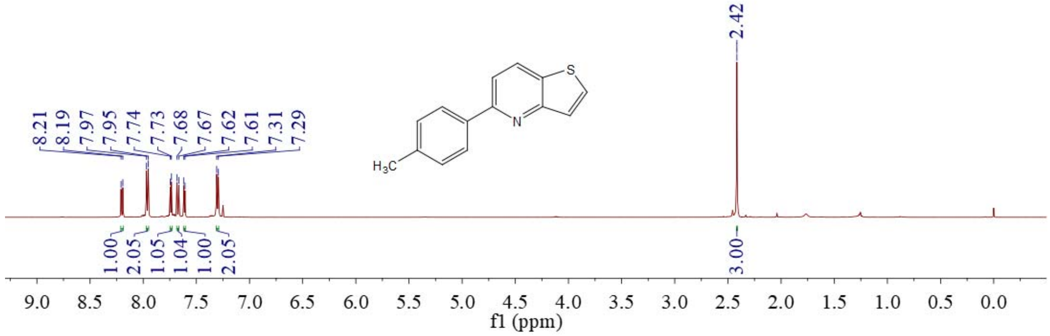

Supplementary Figure 116. ^{1}H-NMR (500 MHz, $CDCl_3$) spectrum of **C53**

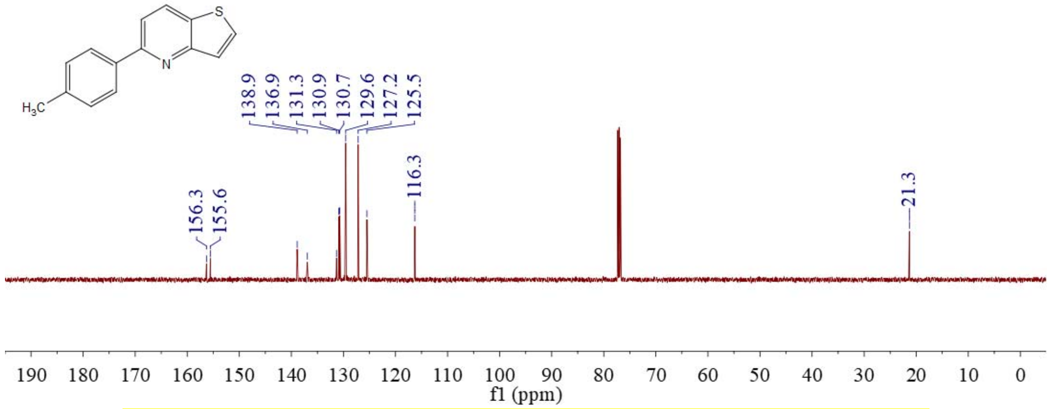

Supplementary Figure 117. ^{13}C-NMR (126 MHz, $CDCl_3$) spectrum of **C53**

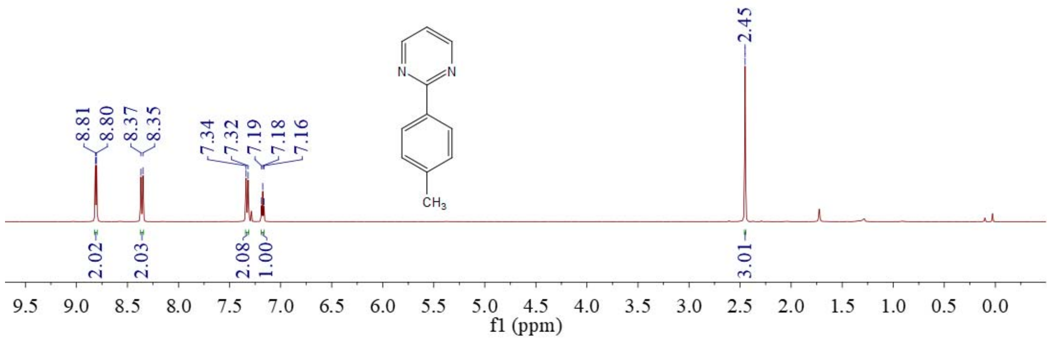

Supplementary Figure 118. ^{1}H-NMR (400 MHz, $CDCl_3$) spectrum of **C54**

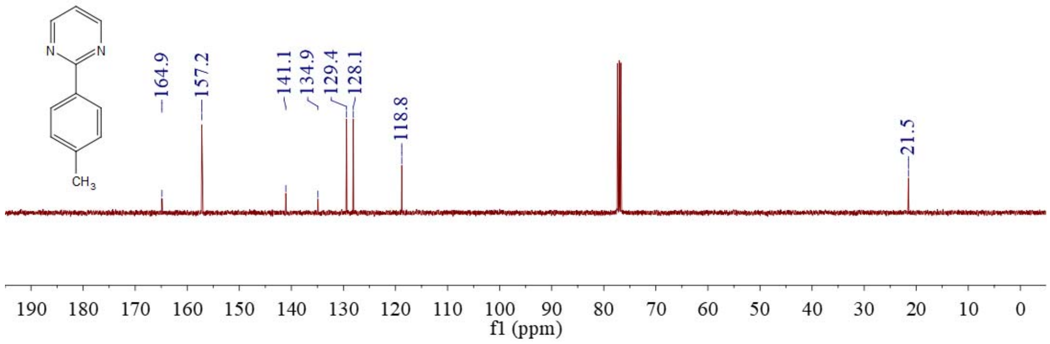

Supplementary Figure 119. ^{13}C-NMR (101 MHz, $CDCl_3$) spectrum of **C54**

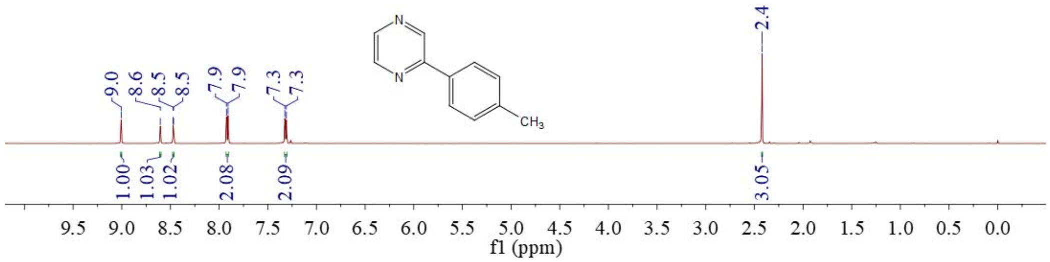

Supplementary Figure 120. ^{1}H-NMR (500 MHz, $CDCl_3$) spectrum of **C55**

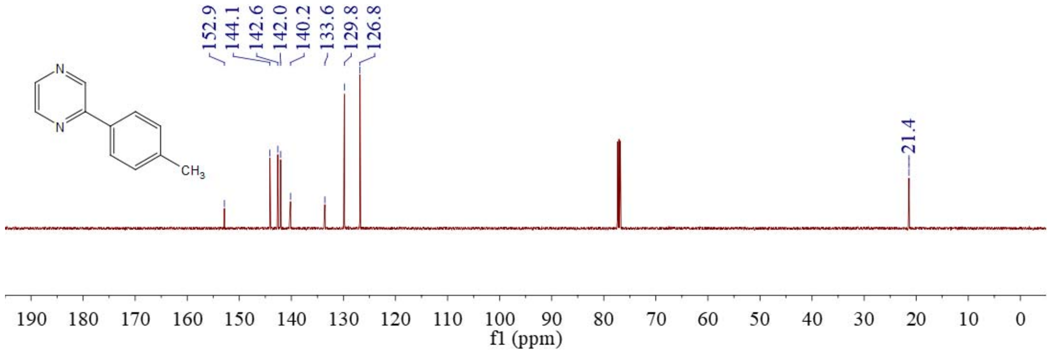

Supplementary Figure 121. ^{13}C-NMR (126 MHz, $CDCl_3$) spectrum of **C55**

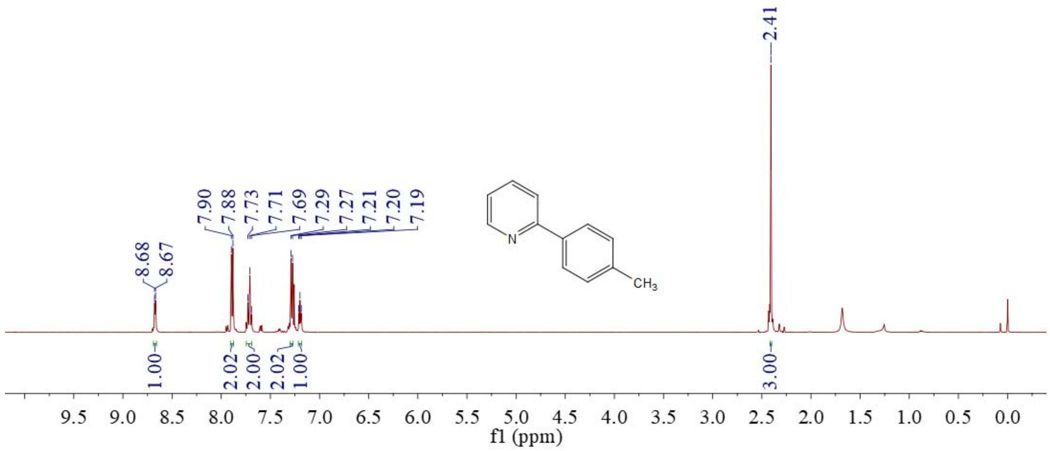

Supplementary Figure 122. ^{1}H-NMR (500 MHz, $CDCl_3$) spectrum of **C56**

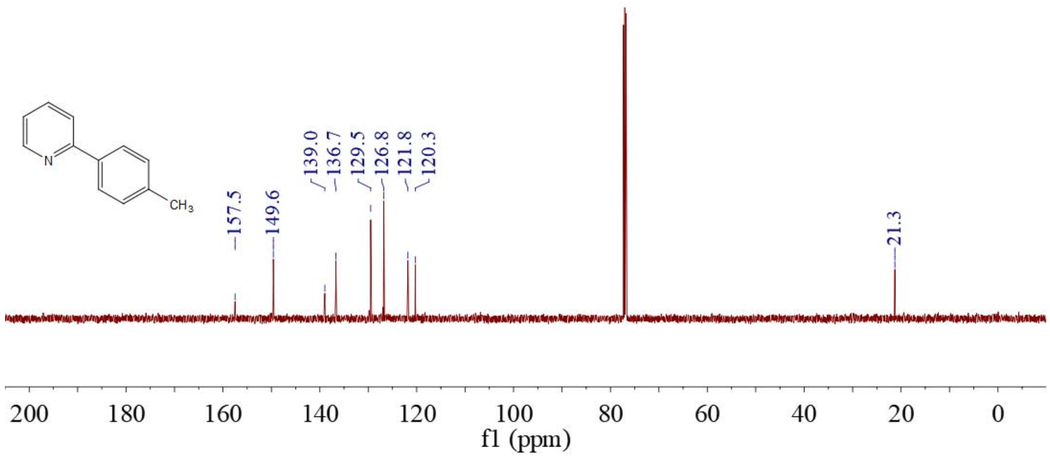

Supplementary Figure 123. ^{13}C-NMR (126 MHz, $CDCl_3$) spectrum of **C56**

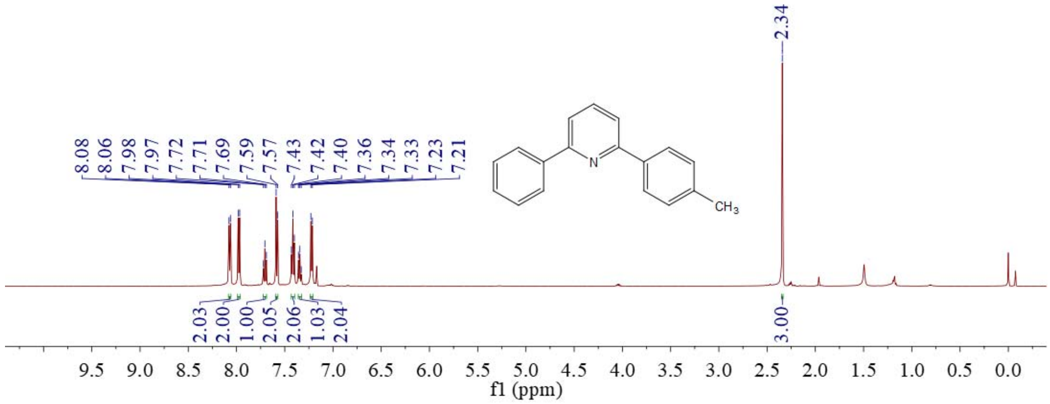

Supplementary Figure 124. ^{1}H-NMR (500 MHz, $CDCl_3$) spectrum of **C57**

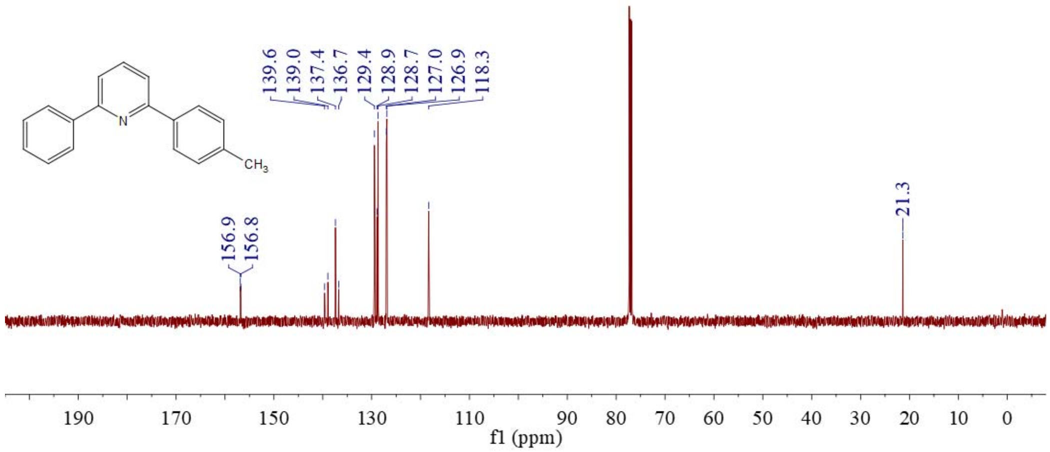

Supplementary Figure 125. ^{13}C-NMR (126 MHz, $CDCl_3$) spectrum of **C57**

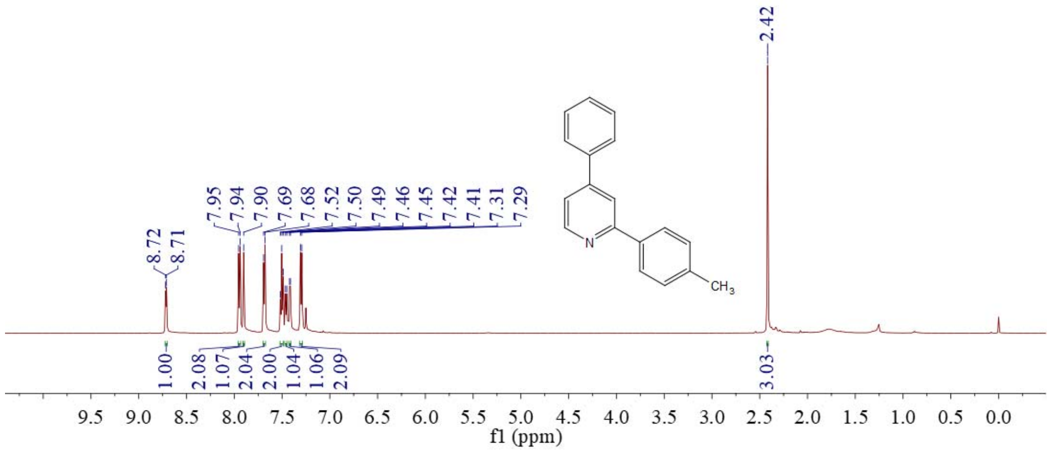

Supplementary Figure 126. ^{1}H-NMR (500 MHz, $CDCl_3$) spectrum of **C58**

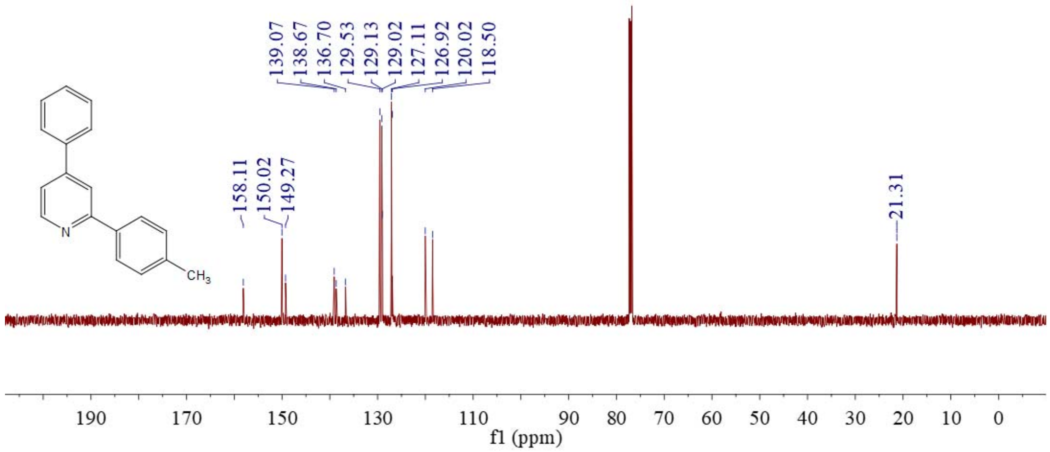

Supplementary Figure 127. ^{13}C-NMR (126 MHz, $CDCl_3$) spectrum of **C58**

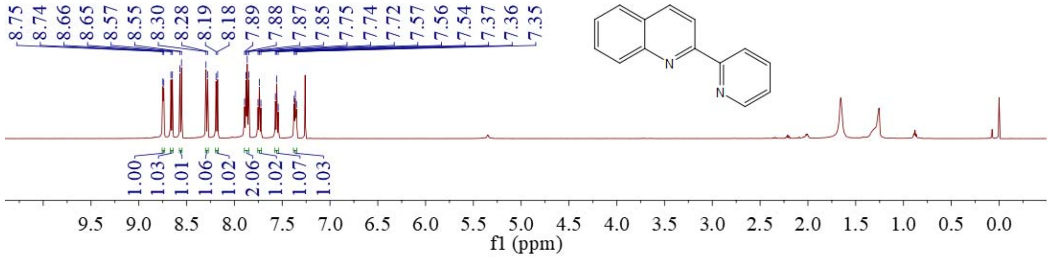

Supplementary Figure 128. ^{1}H-NMR (500 MHz, $CDCl_3$) spectrum of **C59**

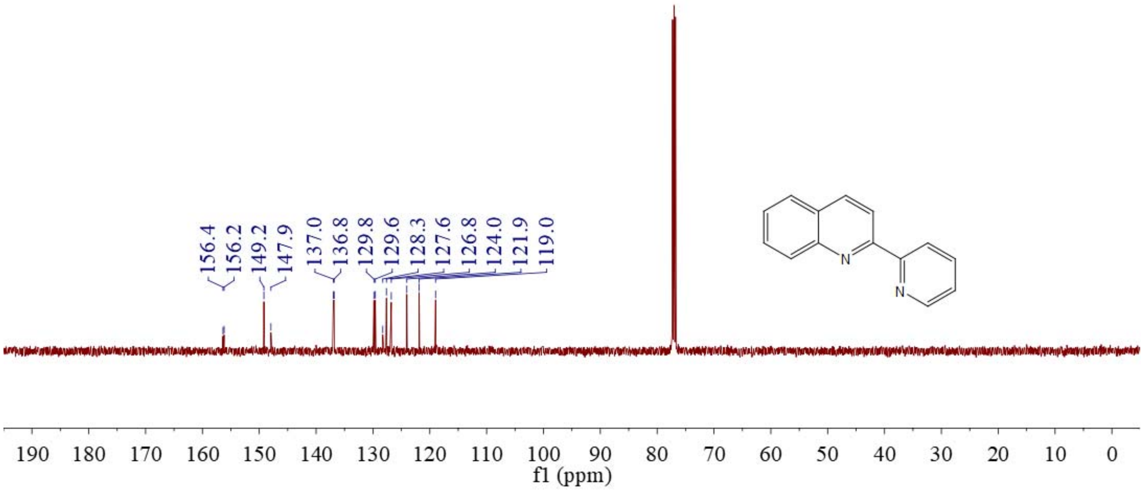

Supplementary Figure 129. ^{13}C-NMR (126 MHz, $CDCl_3$) spectrum of **C59**

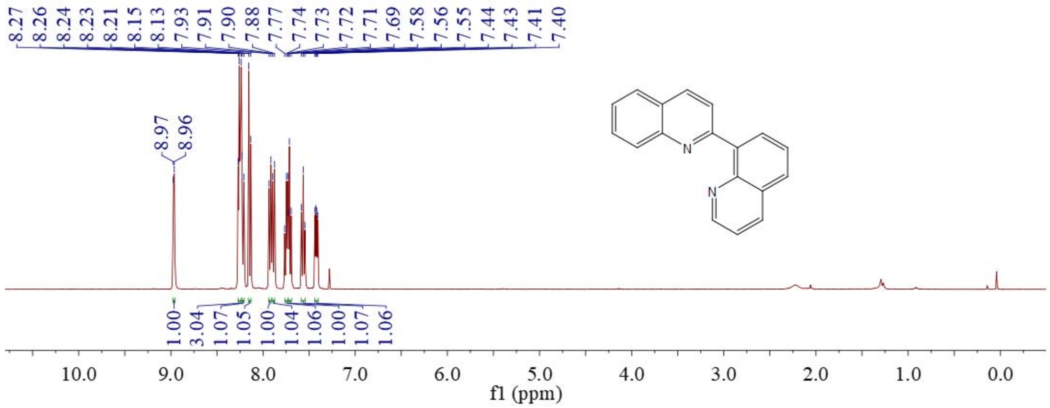

Supplementary Figure 130. ^{1}H-NMR (400 MHz, $CDCl_3$) spectrum of **C60**

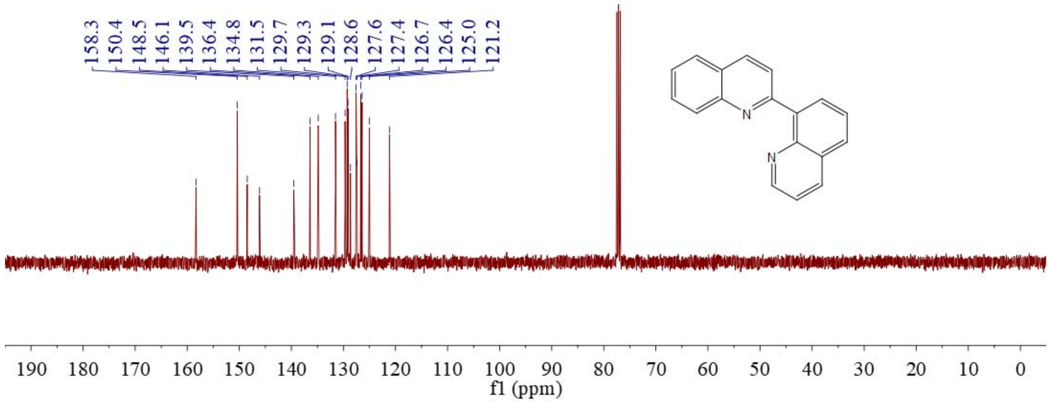

Supplementary Figure 131. ^{13}C-NMR (101 MHz, $CDCl_3$) spectrum of **C60**

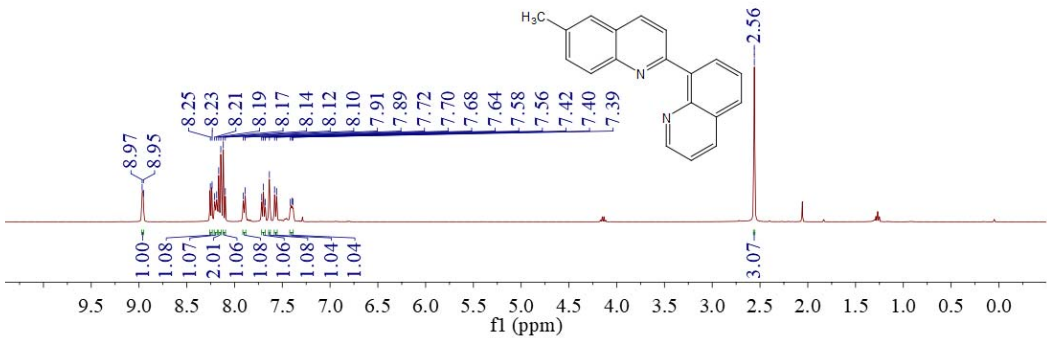

Supplementary Figure 132. ^{1}H-NMR (400 MHz, $CDCl_3$) spectrum of **C61**

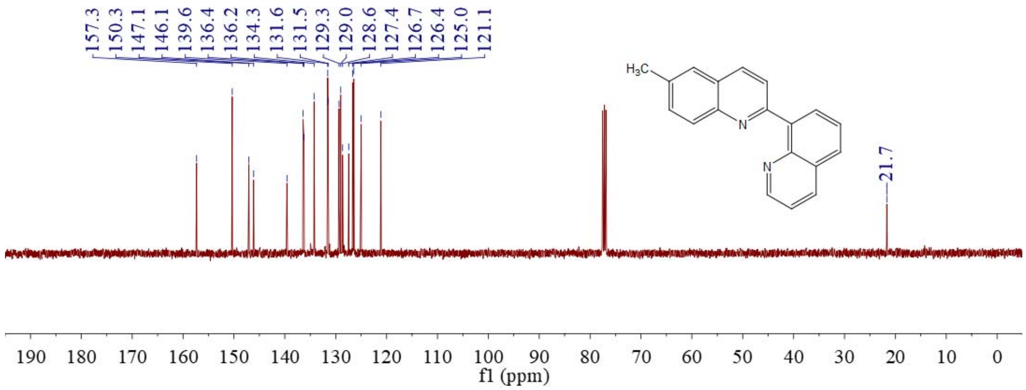

Supplementary Figure 133. ^{13}C-NMR (101 MHz, $CDCl_3$) spectrum of **C61**

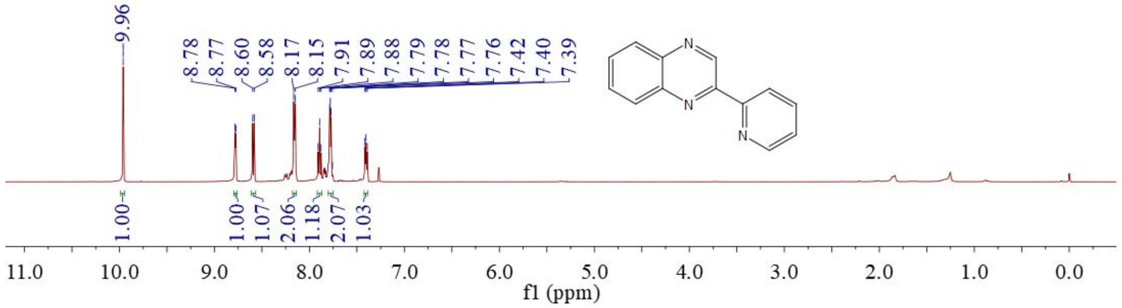

Supplementary Figure 134. ^{1}H-NMR (500 MHz, $CDCl_3$) spectrum of **C62**

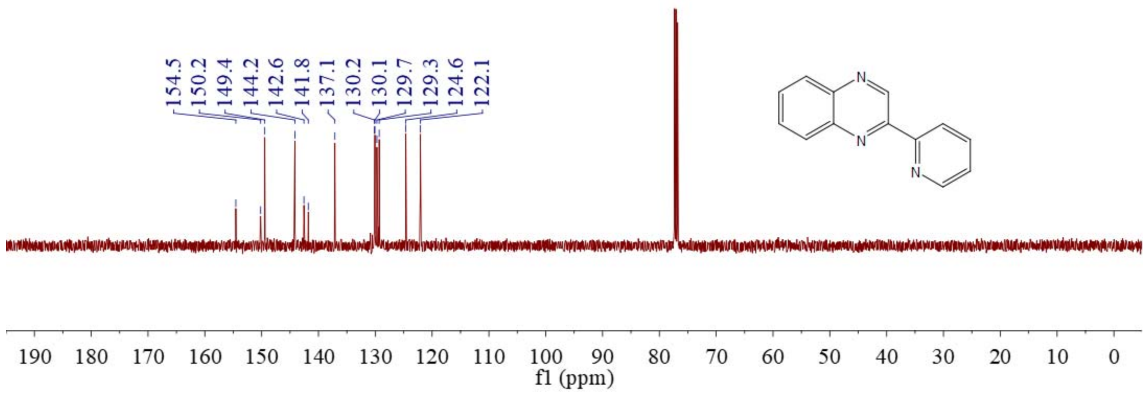

Supplementary Figure 135. ^{13}C-NMR (126 MHz, $CDCl_3$) spectrum of **C62**

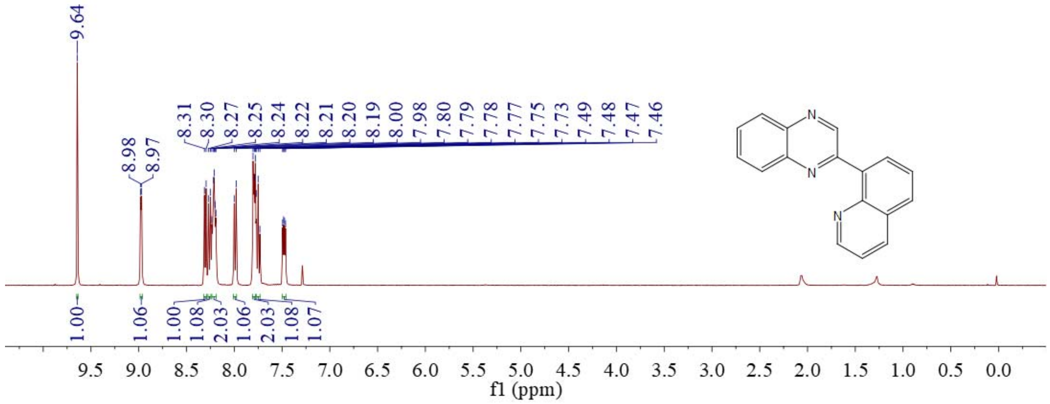

Supplementary Figure 136. ^{1}H-NMR (400 MHz, $CDCl_3$) spectrum of **C63**

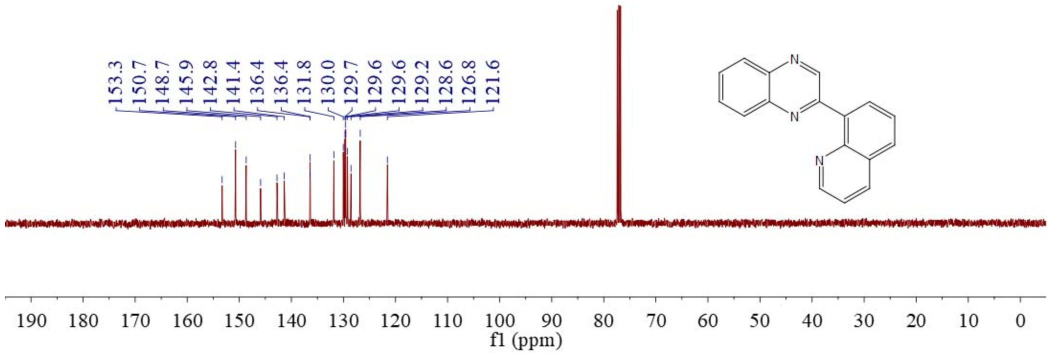

Supplementary Figure 137. ^{13}C-NMR (101 MHz, $CDCl_3$) spectrum of **C63**

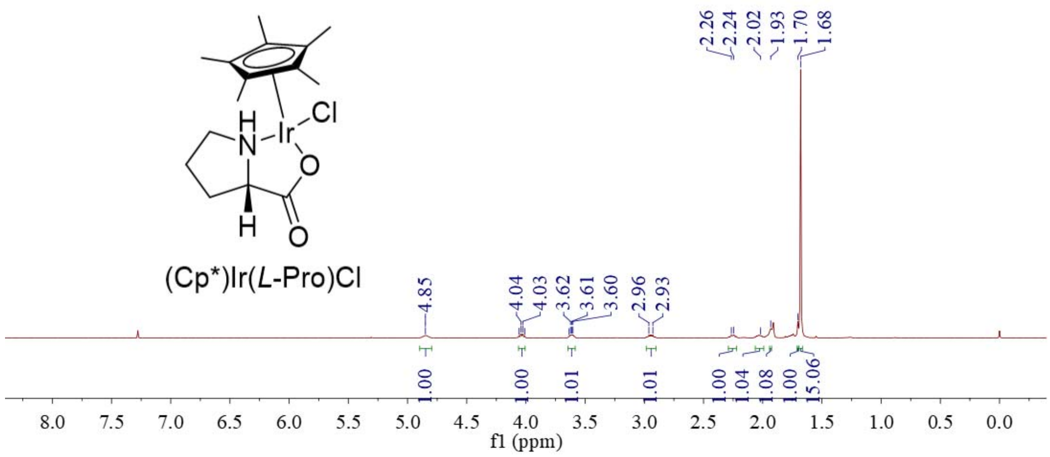

Supplementary Figure 138. ^{1}H-NMR (500 MHz, $CDCl_3$) spectrum of **(Cp*)Ir(*L*-Pro)Cl**

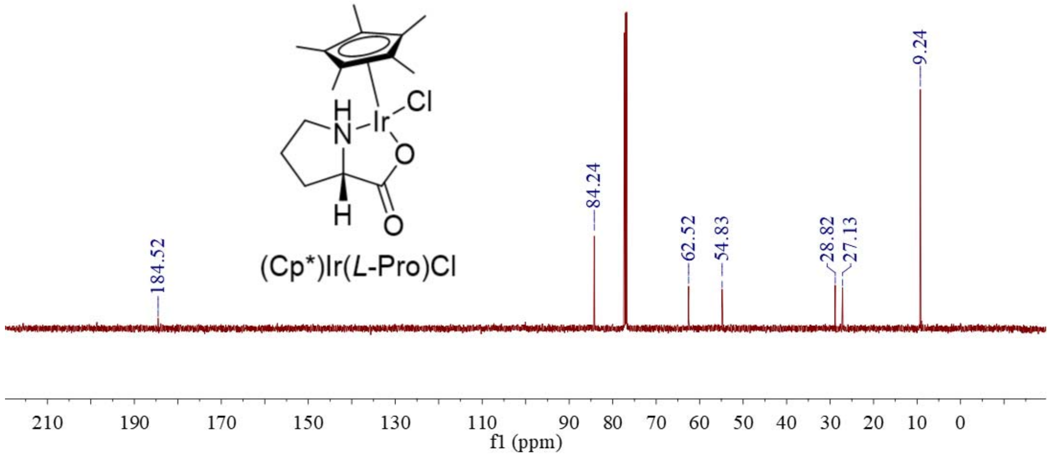

Supplementary Figure 139. ^{13}C-NMR (126 MHz, $CDCl_3$) spectrum of **(Cp*)Ir(*L*-Pro)Cl**

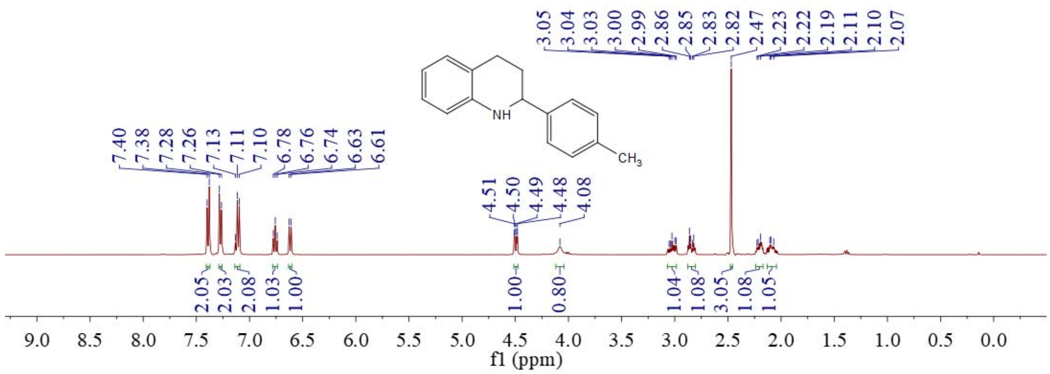

Supplementary Figure 140. ^{1}H-NMR (400 MHz, $CDCl_3$) spectrum of **C1'**

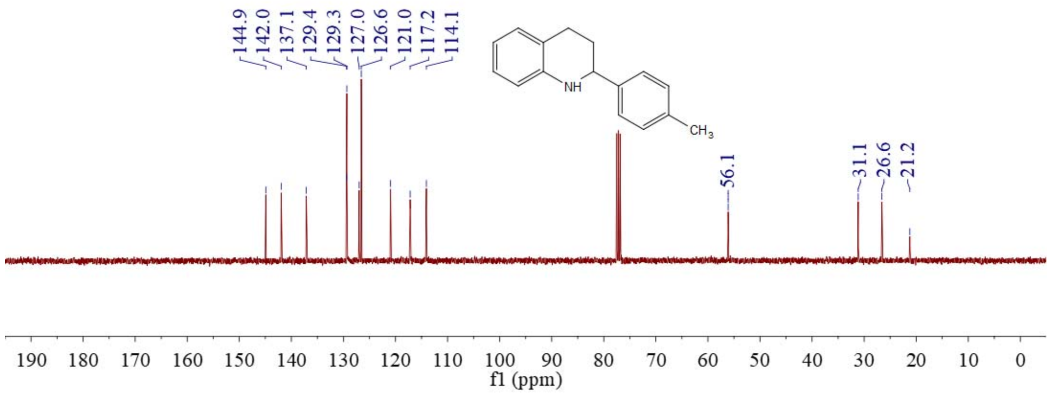

Supplementary Figure 141. ^{13}C-NMR (101 MHz, $CDCl_3$) spectrum of **C1'**

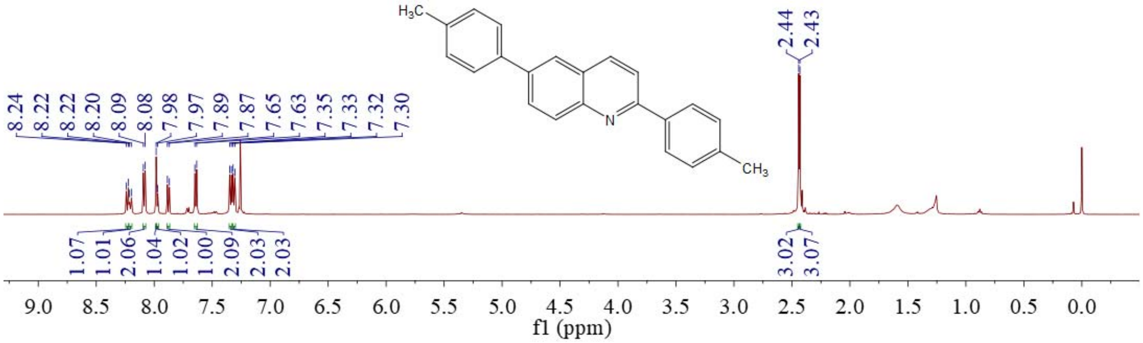

Supplementary Figure 142. ^{1}H-NMR (400 MHz, $CDCl_3$) spectrum of **C37'**

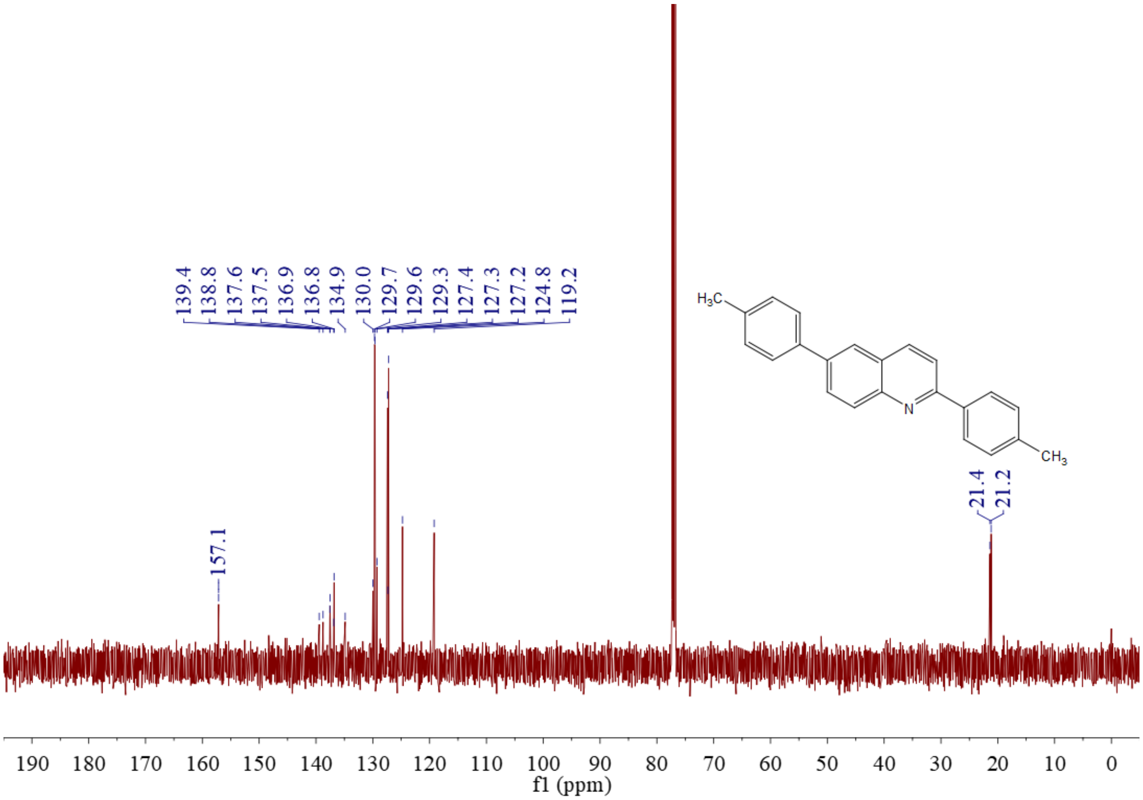

Supplementary Figure 143. ^{13}C-NMR (101 MHz, $CDCl_3$) spectrum of **C37'**

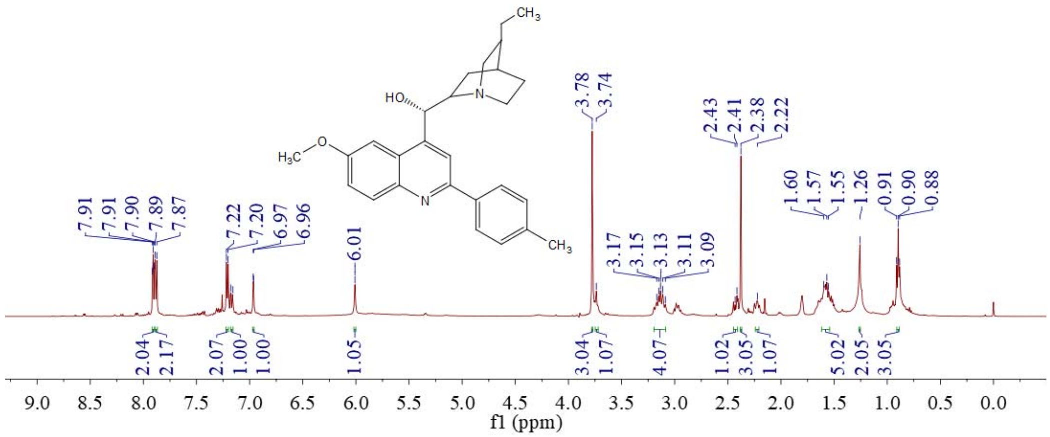

Supplementary Figure 144. ^{1}H-NMR (500 MHz, $CDCl_3$) spectrum of ***p*-tolyl-hydroquinidine hybrid**

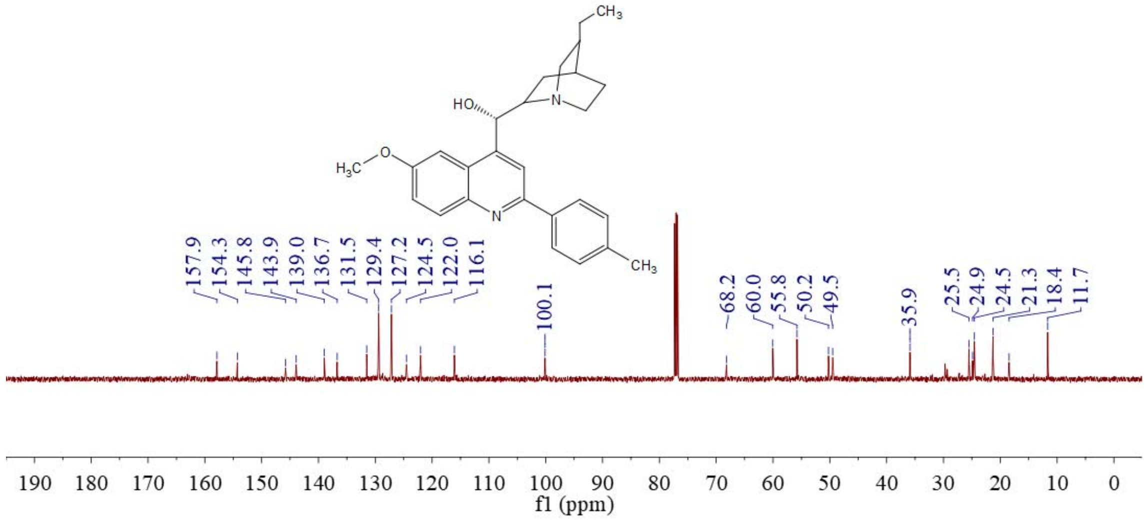

Supplementary Figure 145. ^{13}C-NMR (126 MHz, $CDCl_3$) spectrum of ***p*-tolyl-hydroquinidine hybrid**

Supplementary references

1. Yuan, Y., Jiang, M. B., Wang, T., Xiong, Y. K., Li, J., Guo, H. J., & Lei, A. W. Synergy of anodic oxidation and cathodic reduction leads to electrochemical deoxygenative C2 arylation of quinoline N-oxides. *Chem. Commun.* **55**, 11091-11094 (2019).
2. Ronellenfitsch, M., Wadepohl, H., & Enders, M. Chromium aryl complexes with N-donor ligands as catalyst precursors for selective ethylene trimerization. *Organometallics* **33**, 5758-5766 (2014).
3. Zhan, Z. Z., Ma, H. J., Cui, X. F., Jiang, P. B., Pu, J. H., Zhang, Y. X., & Huang, G. S. Selective synthesis of (*1*H-benzo[*d*]imidazol-2-yl)(phenyl)methanone and quinoxaline from aromatic aldehyde and *o*-phenylenediamine. *Org. Biomol. Chem.* **17**, 5148-5152 (**2019**).
4. Nallagangula, M., Sujatha, C., Bhat, V. T., & Namitharan, K. A nanoscale iron catalyst for heterogeneous direct N- and C-alkylations of anilines and ketones using alcohols under hydrogen autotransfer conditions. *Chem. Commun.* **55**, 8490-8493 (2019).
5. Azizi, K., Akrami, S., & Madsen, R. Manganese (III) porphyrin-catalyzed dehydrogenation of alcohols to form imines, tertiary amines and quinolines. *Chem. Eur. J.* **25**, 6439-6446 (2019).
6. Yao, W., Ge, C. Y., Zhang, Y. L., Xia, X. F., Wang, L., & Wang, D. W. Synthesis of 2-arylisoindoline derivatives catalyzed by reusable 1,2,4-triazole iridium on mesoporous silica through a cascade borrowing hydrogen strategy. *Chem. Eur. J.* **25**, 16099-16105 (2019).
7. Liu, C., Rao, X. F., Song, X. L., Qiu, J. S., & Jin, Z. L. Palladium-catalyzed ligand-free and aqueous Suzuki reaction for the construction of (hetero)aryl-substituted triphenylamine derivatives. *RSC Adv.* **3**, 526-531 (2013).
8. Wang, P., Yang, Z. L., Wang, Z. W., Xu, C. Y., Huang, L., Wang, S. C., Zhang, H., & Lei, A. W. Electrochemical arylation of electron-deficient arenes through reductive activation. *Angew. Chem. Int. Ed.* **58**, 15747-15751 (2019).
9. Kuriyama, M., Matsuo, S., Shinozawa, M., & Onomura, O. Ether-imidazolium carbenes for Suzuki-Miyaura cross-coupling of heteroaryl chlorides with aryl/heteroarylboron reagents. *Org. Lett.* **15**, 2716-2719 (2013).
